# Molecular Networking and On-Tissue Chemical Derivatization for Enhanced Identification and Visualization of Steroid Glycosides by MALDI Mass Spectrometry Imaging

**DOI:** 10.1021/acs.analchem.2c02694

**Published:** 2022-11-08

**Authors:** Domenic Dreisbach, Sven Heiles, Dhaka R. Bhandari, Georg Petschenka, Bernhard Spengler

**Affiliations:** † Institute for Inorganic and Analytical Chemistry, Justus Liebig University Giessen, Heinrich-Buff-Ring 17, 35392 Giessen, Germany; ‡ Leibniz Institute for Analytical Sciences, ISAS−e.V., Otto-Hahn-Straße 6b, 44139 Dortmund, Germany; § Institute of Phytomedicine, University of Hohenheim, Otto-Sander-Straße 5, 70599 Stuttgart, Germany; ∥ Lipidomics, Faculty of Chemistry, University of Duisburg-Essen, Universitätsstraße 5, 45141 Essen, Germany

## Abstract

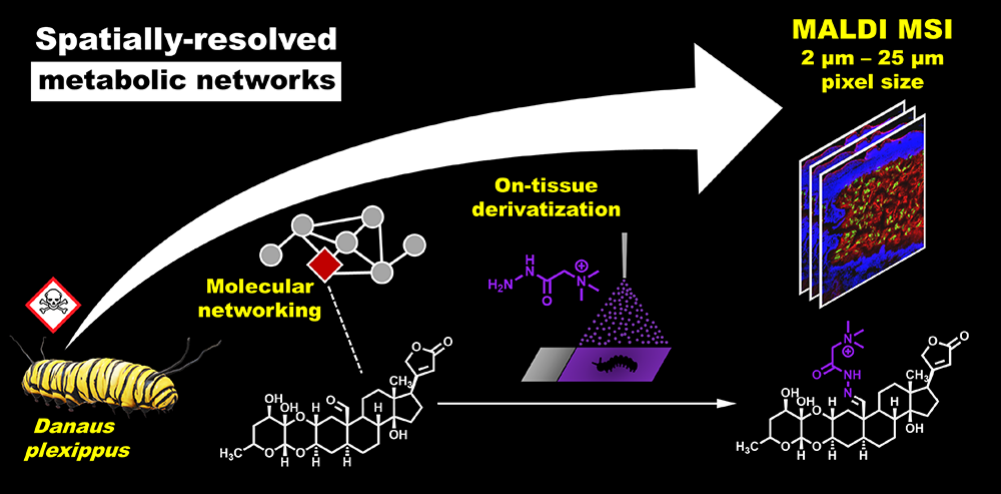

Spatial metabolomics describes the spatially resolved analysis of interconnected pathways, biochemical reactions, and transport processes of small molecules in the spatial context of tissues and cells. However, a broad range of metabolite classes (e.g., steroids) show low intrinsic ionization efficiencies in mass spectrometry imaging (MSI) experiments, thus restricting the spatial characterization of metabolic networks. Additionally, decomposing complex metabolite networks into chemical compound classes and molecular annotations remains a major bottleneck due to the absence of repository-scaled databases. Here, we describe a multimodal mass-spectrometry-based method combining computational metabolome mining tools and high-resolution on-tissue chemical derivatization (OTCD) MSI for the spatially resolved analysis of metabolic networks at the low micrometer scale. Applied to plant toxin sequestration in *Danaus plexippus* as a model system, we first utilized liquid chromatography (LC)–MS-based molecular networking in combination with artificial intelligence (AI)-driven chemical characterization to facilitate the structural elucidation and molecular identification of 32 different steroidal glycosides for the host-plant *Asclepias curassavica*. These comprehensive metabolite annotations guided the subsequent matrix-assisted laser desorption/ionization mass spectrometry imaging (MALDI MSI) analysis of cardiac-glycoside sequestration in *D. plexippus*. We developed a spatial-context-preserving OTCD protocol, which improved cardiac glycoside ion yields by at least 1 order of magnitude compared to results with untreated samples. To illustrate the potential of this method, we visualized previously inaccessible (sub)cellular distributions (2 and 5 μm pixel size) of steroidal glycosides in *D. plexippus*, thereby providing a novel insight into the sequestration of toxic metabolites and guiding future metabolomics research of other complex sample systems.

## Introduction

Metabolic networks describe interconnected pathways of biochemical reactions and transport processes of low-molecular-weight chemical species (metabolic intermediates, hormones, signaling molecules, secondary metabolites) within living organisms.^1−3^ The processes within metabolic networks can be temporally and spatially organized.^4^ In this context, the interest and ever-growing need to spatially characterize biological phenomena *in situ* have grown rapidly, which stimulated the development of enabling technologies. In particular, mass spectrometry imaging (MSI) methods have emerged as one of the fastest-growing mass spectrometry (MS) fields over the past decade.^5,6^ MSI provides for nontargeted spatially resolved analysis of molecular species. Not only the analytes of interest but also hundreds of other chemical species can be detected, identified, and visualized simultaneously, thereby aiming to link molecular structures to biological functions and origin.^7,8^ Among the different MSI methods, MALDI MSI is the predominant bioanalytical tool in chemistry, biology, and medicine, and recent technical advances have considerably improved the performance characteristics regarding molecular coverage, sensitivity, and spatial resolution. For instance, Kompauer *et al.* combined a coaxial ion source geometry (MS-inlet and laser beam path coaxially aligned to the sample-surface normal) with a custom-made long-working-distance objective lens, allowing the visualization of lipid, metabolite, and peptide distributions in complex biological samples at atmospheric pressure with an effective lateral resolution of 1.4 μm.^9^ However, sensitivity is a significant barrier for visualizing metabolic networks via MALDI MSI.^10^ The problem of generally low MALDI ionization efficiencies (ion yields down to 10^–6^ for some analyte classes^11,12^) is exacerbated by the decreasing amount of ablated material in high-resolution MSI. Multiple approaches to increase the MALDI ion yield have been reported, including optimized MALDI laser wavelength^13,14^ and laser-induced post-ionization (MALDI-2).^15^ For example, Niehaus *et al.* developed an ion source for transmission-mode MALDI-2 MSI, demonstrating improved analytical sensitivity by several orders of magnitude for phospho- and glycolipids with pixel sizes of 1 μm.^16^ However, this approach requires novel and complex instrumentation, and the limited availability of commercial MALDI-2 MSI instruments prevents broader applicability.^17,18^

As a powerful alternative, on-tissue chemical derivatization (OTCD) of target analytes with precharged moieties can counteract isobaric matrix interferences, ion suppression, and low intrinsic ionization efficiencies.^19^ Introduced in 2013 by Cobice *et al*., hydrazine-forming reagents have been used to target ketone-containing substrates and products of the glucocorticoid amplifying enzyme 11β-HSD1 in rat adrenal gland and mouse brain.^20^ Afterward, various studies utilized OTCD-MSI to gain additional or previously inaccessible insight into spatial distributions and molecular structures in the field of biological and medical research.^21−25^ Despite all of these studies that demonstrate the potential of selectively enhancing ion yields, OTCD methods can be limited by spatial artifacts and analyte washing effects, thus preventing the visualization of (sub)cellular metabolite distributions.

To comprehensively explore and interpret metabolic networks, the corresponding individual chemical components have to be discovered and identified. However, molecular identification and elucidating chemical structures are mostly restricted to compounds for which mass spectrometric reference data are archived in spectral libraries (e.g., commercially available chemicals).^26−28^ Since its introduction in 2012, molecular networking from the Global Natural Products Social Molecular Networking (GNPS) infrastructure has become a key method to organize and annotate nontargeted LC–MS^1^ and −MS^2^ data.^29−31^ Utilizing spectral similarity (with the assumption of structural similarity), related molecular species are connected, and annotations from spectral library matching can be propagated through generated molecular networks, thereby pushing the frontier of conventional database search and facilitating the structural elucidation of unknown chemical compounds. Dührkop *et al*. developed the computational method CSI (Compound Structure Identification):FingerID,^32^ which combines fragmentation-tree calculations and machine-learning techniques for *in silico* annotations of MS^2^ spectra based on substantially larger molecular structure databases. This method tackled the major bottleneck of the limited availability of chemical compounds represented in mass spectral libraries. Very recently, the same authors described CANOPUS (class assignment and ontology prediction using mass spectrometry), a computational tool that utilizes a deep neural network to predict chemical compound classes and to perform structural analysis of unknown metabolites using high-resolution MS^2^ data.^33−35^ Therefore, combining these different computational metabolome mining tools could provide a powerful platform to comprehensively explore metabolic networks and identify their respective chemical species. Here, we combined LC–MS-based molecular networking and artificial intelligence (AI)-driven chemical classification with OTCD MALDI MSI for the unbiased spatial-metabolomic characterization of plant toxin sequestration in the monarch butterfly (*Danaus plexippus*) (Figure S1). In this fascinating antagonistic interaction, the monarch butterfly absorbs and accumulates steroidal plant toxins (cardiac glycosides) from milkweed host plants (*Asclepias* spp.) into its own body tissues to obtain a chemical defense against predators.^36−38^

First, we generated a metabolomic ″in-house″ database of *A. curassavica* consisting of 32 steroidal glycosides. Next, these annotations were utilized to guide the spatially resolved MSI-based analysis of cardiac glycoside sequestration in monarch caterpillar tissues and cells. We mitigated the problem of low intrinsic ionization efficiencies by the selective chemical tagging of carbonyl-containing cardiac glycosides with precharged moieties while retaining spatial information. To illustrate the potential of our methodology, we imaged (sub)cellular distributions of derivatized cardiac glycosides in epithelial cells, Malpighian tubules, and various body tissues in unprecedented detail.

## Experimental Section

### Chemicals

Acetonitrile and water (HiPerSolv) were purchased from VWR International GmbH (Darmstadt, Germany). 2,5-Dihydroxybenzoic acid (DHB) was purchased from Merck (Darmstadt, Germany). Trifluoroacetic acid (TFA) was purchased from AppliChem GmbH (Darmstadt, Germany). Girard’s reagent T (GirT) was purchased from Merck (Darmstadt, Germany). Formic acid (FA) was purchased from Fisher Scientific (Schwerte, Germany).

### Plants and Insects

Samples of *Asclepias curassavica* were obtained from plants cultivated at the Institute of Insect Biotechnology (Justus Liebig University, Giessen, Germany). Caterpillars of *D. plexippus* were raised on *A. curassavica* under controlled conditions at the same institute.

### Sample Preparation for LC–MS

*A. curassavica* leaf samples were harvested, immediately freeze-dried, ground to a fine powder, and subsequently extracted for nontargeted LC–MS^2^ experiments. The detailed experimental procedure is provided in Supplementary Note 1.

### Sample Preparation for OTCD MALDI MSI

The established cryosectioning protocol to obtain longitudinal tissue sections for final instar larvae (Figure S2) of excellent quality regarding morphological preservation is reported elsewhere.^39^ Prior to on-tissue chemical derivatization, tissue sections were brought to room temperature in a desiccator for 45 min to avoid condensation of humidity on the sample surface. Optical microscopic images of tissue sections before and after OTCD, after matrix application, and after MSI analysis and of hematoxylin–eosin (H&E)-stained tissue sections were obtained using a Keyence VHX-5000 digital microscope (Keyence Deutschland GmbH, Neu-Isenburg, Germany) equipped with a VH-Z250R objective lens. A volume of 35 μL of GirT solution (15 mg/mL in MeOH/water 7:3 v/v adding 0.2% TFA) was sprayed onto the tissue section at a flow rate of 7 μL/min using an ultrafine pneumatic sprayer system (SMALDIPrep, TransMIT GmbH, Giessen, Germany). The nebulizing nitrogen gas pressure was 1 bar, and the rotation was set to 500 rpm. After derivatization, samples were kept at room temperature in a desiccator for 2 h without any further incubation. A volume of 100 μL DHB matrix solution (30 mg/mL in MeOH/water 1:1 v/v adding 0.1% TFA) was sprayed onto the tissue section at a flow rate of 5 μL/min using the same ultrafine pneumatic sprayer system. After MSI analysis, tissue sections were washed with ethanol (70%) for 2 min to remove the matrix layer followed by H&E staining for histological classification (see Supplementary Protocol 1).

### Instrumentation for MSI

High-resolution (5 to 25 μm step size) MALDI MSI and MALDI MS^2^ experiments were performed using an autofocusing AP-SMALDI^5^ AF ion source^40^ (TransMIT GmbH, Giessen, Germany) coupled to an orbital trapping mass spectrometer (Q Exactive HF, Thermo Fisher Scientific GmbH, Bremen, Germany). For higher-resolution (2 μm step size) MALDI MSI experiments, a prototype AP-SMALDI AF ion source coupled to a Q Exactive Orbitrap mass spectrometer (Thermo Fisher Scientific GmbH, Bremen, Germany) was used. Detailed experimental parameters are provided in Supplementary Note 2.

### Instrumentation for HPLC–MS

All HPLC–MS experiments were performed using a Dionex UltiMate 3000 HPLC instrument (Thermo Fisher Scientific, Massachusetts, USA) coupled to a Q Exactive HF-X Orbitrap mass spectrometer (Thermo Fisher Scientific, Bremen, Germany). Analytes were separated on a Kinetex C18 reversed-phase column (2.6 μm, 100 × 2.1 mm, Phenomenex, Torrance, USA). Detailed experimental parameters for HPLC–MS/MS analysis are provided in Supplementary Note 3.

### MSI Data Analysis

The Xcalibur Qual Browser (Thermo Fisher Scientific, Massachusetts, USA) was used to display mass spectra. Ion images of selected *m*/*z* values were generated using the MIRION^41^ imaging software (v3.3.64.20, TransMIT GmbH, Giessen, Germany). All MS images were generated without the use of image processing steps such as smoothing or interpolation. Ion images were normalized to the total ion count (TIC) per pixel. The resulting ion images were finally adjusted in brightness for optimal visualization. MSiReader^42^ was used to extract intensity profiles for defined regions of interest. Lipids and metabolites were assigned based on exact mass measurements, LC–MS^2^ experiments, on-tissue MALDI MS^2^, and METASPACE^43^ annotations.

### Molecular Networking and *In Silico* Molecular Characterization

Raw mass spectra were converted to mzXML files using MSConvert (Proteo Wizard, v.3.0.11579). MS^1^ feature extraction and MS^2^ processing were performed using MZmine 2^44^ (see Supplementary Note 4 for detailed information). Feature-based molecular networks (FBMNs) were generated using the FBMN workflow from the GNPS analysis infrastructure (see Supplementary Note 5 for detailed information) and visualized with Cytoscape^45^ (v.3.8.0). SIRIUS (v.4.5.3) and the included CSI:FingerID and CANOPUS tools were used for *in silico* characterization of LC–MS^2^ data.

## Results and Discussion

### Molecular Networking Combined with AI-Driven Molecular Characterization Defines the Steroidal Glycoside Composition of *A. curassavica*

**Figure 1:**
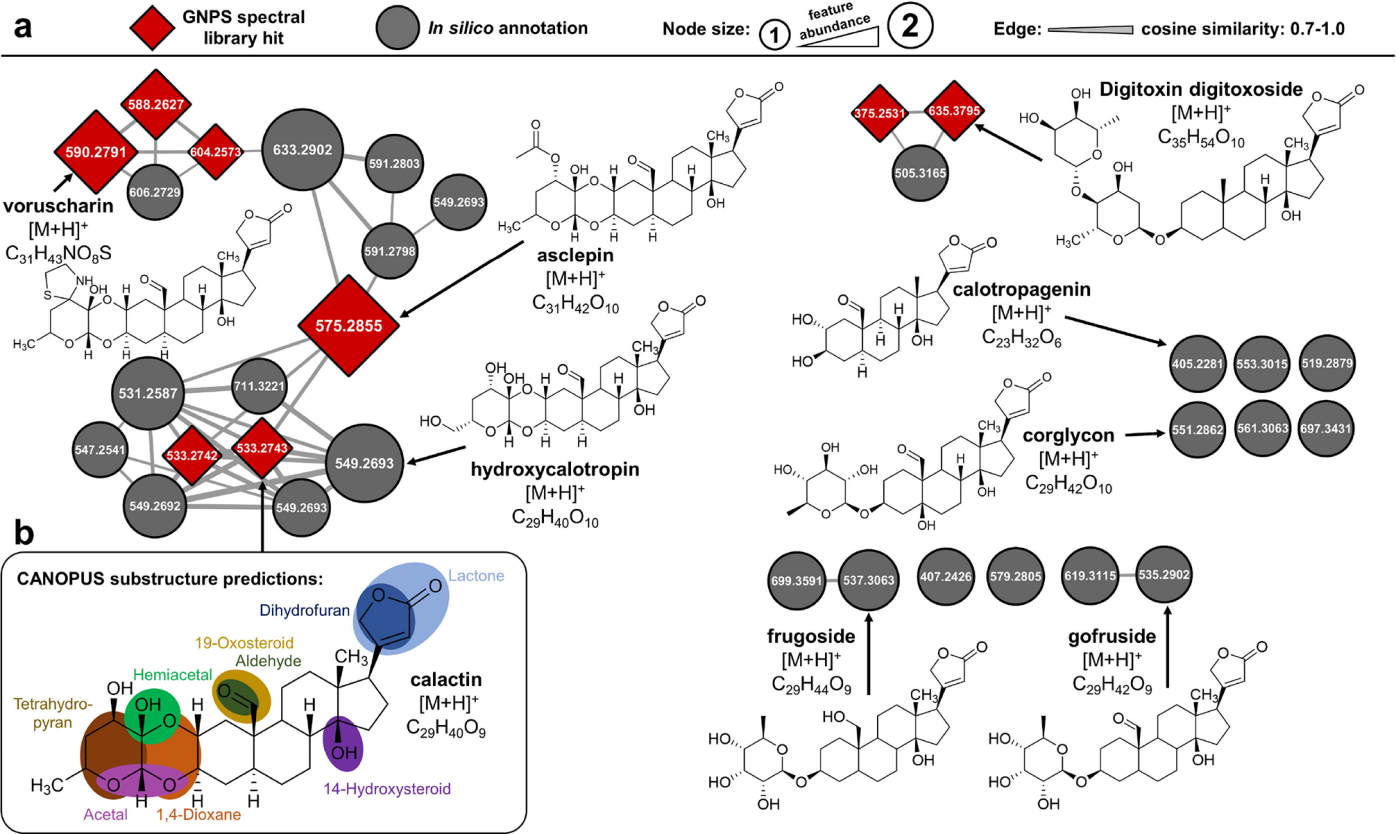
Feature-based molecular networking and *in silico* systematic classification, substructure prediction, and annotation for cardiac glycosides in *A. curassavica*. (a) FBMN results for nontargeted LC–MS^2^ data of *A. curassavica* leaf showing molecular networks related to cardiac glycosides. The node size proportionally represents the LC–MS feature abundance (peak area), and the increased edge thickness corresponds to the higher cosine similarity (0.7 to 1.0). CANOPUS was used for compound classification to discover additional candidates that were not part of the original molecular network. In total, 32 different cardiac glycosides were identified based on 8 GNPS spectral library hits (red nodes) and 24 *in silico* annotations (gray nodes) using SIRIUS (CSI:FingerID). *In silico* annotations were also manually evaluated (Supplementary Data 1). (b) CANOPUS substructure predictions (posterior probability > 75%) for calactin to facilitate structural elucidation and fragmentation pathway analysis.

First, we performed nontargeted LC–MS^2^ experiments of leaf extracts and utilized state-of-the-art molecular networking tools in combination with AI-driven molecular characterization to acquire a metabolomic profile for the host plant *A. curassavica.*Figure S3 shows the comprehensive FBMN results that consisted of 1175 mass spectral nodes organized into 89 independent molecular families. In total, we obtained 145 spectral library hits (red nodes), allowing the classification of the corresponding molecular networks. Figure 1a shows the molecular network for cardiac glycosides, which are potent inhibitors for Na^+^/K^+^-ATPase, a cation carrier ubiquitously expressed in animal cells. Utilizing AI-driven compound class prediction, 12 additional LC–MS^2^ features were classified as cardiac glycosides (Figure 1a, bottom right), which were not part of the original molecular network due to different fragments in the MS^2^ spectra (Supplementary Note 3). In total, 32 different cardiac glycosides were identified based on 8 GNPS spectral library hits (red nodes) and 24 *in silico* annotations (gray nodes) (Table S1). To evaluate *in silico* annotations, we manually investigated the respective fragmentation spectra (Supplementary Data 1). Compared to the most recent studies^46,47^ regarding toxic steroidal glycoside composition in *A. curassavica*, we found 20 additional cardiac glycosides that were also absent from mass spectral libraries. Computational substructure predictions (as depicted in Figure 1b for calotropin) facilitated the structural elucidation of annotated cardiac glycosides. Whereas all detected and classified cardiac glycosides are 19-oxosteroids (besides digitoxigenin (*m*/*z* 375.2531) and two derivatives (Figure 1a), the molecular network exclusively contains cardiac glycoside with an aldehyde group. Therefore, cardiac glycosides having a hydroxyl function at C19 (frugoside, C_29_H_44_O_9_ at *m*/*z* 537.3063; antiaroside B, C_35_H_54_O_14_ at *m*/*z* 699.3591; strophanthidol, C_23_H_34_O_6_ at *m*/*z* 407.2426) were not part of the cardiac glycoside network due to different characteristic fragments (Figure S4 and Supplementary Note 5). Despite having an aldehyde group at C19, gofruside (*m*/*z* 535.2902, C_29_H_42_O_9_) and corglycone (*m*/*z* 551.2860, C_29_H_42_O_10_) were also excluded from the molecular network due to ether-bond-linkage between the aglycone and glycoside unit (instead of 1,4-dioxane linkage), and the precursor calotropagenin (*m*/*z* 405.2281, C_23_H_32_O_6_) was excluded due to the absence of a glycoside unit. Importantly, FBMN resolved several isomers for *m*/*z* 533.2741 (C_29_H_40_O_9_), *m*/*z* 549.2693 (C_29_H_40_O_10_), and *m*/*z* 591.2803 (C_31_H_42_O_11_) that have similar MS^2^ spectra but distinct retention times and thus would have remained hidden in classical molecular networking. The molecular network can be divided into subnetworks depending on different chemical subgroups (thiazolidine/thiazoline, acetyloxy, and hydroxy/ketone group) in the glycoside unit, which is represented by the spectral library annotations for voruscharin, asclepin, and calotropin in Figure 1a. In addition, FBMN enabled quantitative analysis by using the LC–MS feature abundance (peak area) showing that asclepin (C_31_H_42_O_10_ at *m*/*z* 575.2855), 16α-acetoxyasclepin (C_33_H_44_O_12_ at *m*/*z* 633.2902), uscharidin (C_29_H_38_O_9_ at *m*/*z* 531.2587), and voruscharin (C_31_H_43_NO_8_S at *m*/*z* 590.2791) are the most abundant cardiac glycosides in *A. curassavica*.

### Visualizing Metabolic Networks of Cardiac Glycoside Sequestration in *D. plexippus*

**Figure 2:**
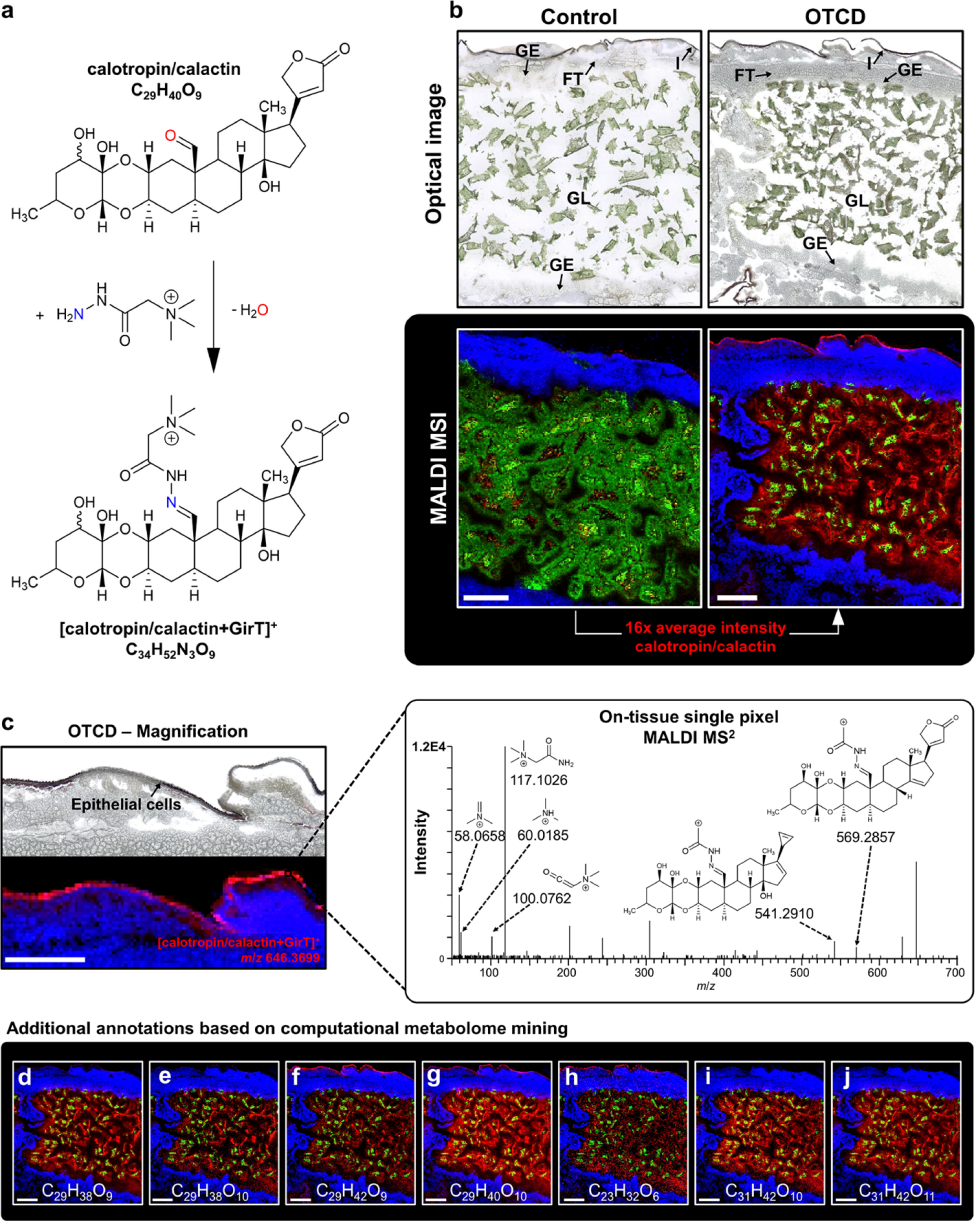
High-resolution MALDI MSI (25 μm step size) for chemically derivatized cardiac glycosides in longitudinal *D. plexippus* sections. (a) Schematic of the reaction between calotropin/calactin and the GirT reagent. (b) Optical images showing the analyzed area for conventional MALDI (left) and OTCD MALDI MSI (right). The corresponding RGB overlay images were normalized to the same intensity scale and show the spatial distribution of calotropin/calactin in red as [M + K]^+^ at *m*/*z* 571.2306 for control (left) and [M + GirT]^+^ at *m*/*z* 646.3699 for OTCD (right), pheophytin a as [M + K]^+^ at *m*/*z* 909.5291 in green, and PS(36:3) as [M + K]^+^ at *m*/*z* 810.5046 in blue. (c) Magnified view for the integument area of the OTCD experiment highlighting the accumulation of the toxin in the epithelial cells of the integument. Corresponding OTCD MALDI MS^2^ spectrum of calotropin/calactin as [M + GirT]^+^ at *m*/*z* 646.3699 acquired from a single pixel at the *D. plexippus* integument. (d–j) RGB overlay images showing additional derivatized cardiac glycosides in red. (d) Uscharidin ([M + GirT]^+^, *m*/*z* 644.3541), (e) hydroxyuscharidin ([M + GirT]^+^, *m*/*z* 660.3489), (f) gofrugoside ([M + GirT]^+^, *m*/*z* 648.3828), (g) calotoxin/hydroxycalactin/hydroxycalotropin ([M + GirT]^+^, *m*/*z* 662.3648), (h) calotropagenin ([M + GirT]^+^, *m*/*z* 518.3233), (i) asclepin ([M + GirT]^+^, *m*/*z* 688.3806), and (j) hydroxyasclepin ([M + GirT]^+^, *m*/*z* 704.3756). Scale bars: (b, d–j) 1 mm and (c) 500 μm.

Next, we utilized our metabolomic ″in-house″ database to guide *in situ* visualization of metabolic networks related to cardiac glycoside sequestration in fifth instar longitudinal *D. plexippus* caterpillar sections. However, previous studies showed that detecting and visualizing steroidal compounds using MSI coupled with soft ionization techniques are exceptionally difficult due to poor ion yields and the presence of interfering molecules in the low mass-to-charge-number range (*m*/*z* < 500).^20−25^ Therefore, we used Girard’s reagent T (GirT) to enhance detection sensitivity while retaining spatial information. We developed an ultrafine OTCD method by utilizing a high reagent concentration combined with a low total spray volume and flow rate that was followed by incubation without increased humidity and optimized matrix application (Figures S5–S9 and Supplementary Note 6 for details regarding method development). Girard’s reagent T reacted with carbonyl-containing cardiac glycosides, resulting in hydrazone formation and a positively charged triethylamine function (Figure 2a). To analyze and demonstrate the ion signal boost provided by the covalent charge-tagging approach, we also performed MSI experiments without OTCD, but with identical experimental parameters, of the adjacent section.

The optical images (Figure 2b) display the analyzed area consisting of the gut lumen (GL) that contains the *A. curassavica* plant material and is surrounded by the gut epithelium (GE), fat tissue (FT), and integument (I). The corresponding MSI results for three selected ion signals are shown in red–green–blue overlay (RGB) images obtained with a 25 μm step size (Figure 2b). The red color channel highlights the spatial distribution of the toxic cardiac glycoside isomers calotropin/calactin ([M + K]^+^ and [M + GirT]^+^, respectively). The green color channel represents pheophytin a ([M + K]^+^), which is characterized as a chlorophyll A molecule without the central Mg^2+^ cation and serves as a marker for the plant material. The spatial distribution of PS(36:3) ([M + K]^+^) is shown in blue, highlighting the gut epithelium and fat tissue. In general, for OTCD, no additional adducts (H^+^, Na^+^, K^+^) of cardiac glycosides were detected, and derivatized ions were exclusively detected as GirT-carrying ions. For comparison, both RGB images were normalized to the same intensity scale, thereby demonstrating that the average signal intensity of calotropin/calactin was increased by 16-fold ([M + GirT]^+^ relative to the dominant non-OTCD adduct [M + K]^+^, see Figure S10 for box plots), also increasing the pixel coverage to 72% (35% for the control). Hence, OTCD MALDI MSI significantly improved the extracted biological information and vividly revealed that steroidal glycosides are extracted from the *A. curassavica* plant material, absorbed into the gut epithelium, and stored in the integument of the caterpillar. Importantly, derivatized species preserved their fidelity and spatial integrity, therefore allowing us to spatially resolve the fine distribution of accumulated cardiac glycosides in the single layer of epithelial cells in the integument, as shown in Figure 2c for calotropin/calactin. The corresponding single-pixel mass spectrum (Figure S11) demonstrates that [calotropin/calactin+GirT]^+^ is one of the most abundant signals (ion intensities of ∼1 × 10^5^), thus enabling *in situ* identification by on-tissue single-pixel MALDI MS^2^ (Figure 2c). In total, we detected and annotated 19 derivatized cardiac glycosides in *D. plexippus*, demonstrating that our LC–MS-based molecular networking/AI-classification approach successfully guided and facilitated the spatial molecular characterization using MSI. Our OTCD MALDI MSI results determined that the majority of cardiac glycosides are taken up and stored in the integument of the caterpillar (e.g., calotropin/calactin (Figure 2b), low amounts of uscharidin (Figure 2d) and hydroxyuscharidin (Figure 2e), gofrugoside (Figure 2f), calotoxin/hydroxycalactin/hydroxycalotropin (Figure 2g), and calotropagenin (Figure 2h)). In contrast, asclepin (Figure 2i) and hydroxyasclepin (Figure 2j) belong to the most abundant toxic glycosides in *A. curassavica* (Figure 1a) and were exclusively located in the gut lumen. Thus, our MSI results demonstrate that small incremental changes in the chemical structure directly correlate to the selectivity of plant toxin sequestration in *D. plexippus*.

### Visualizing (Sub)Cellular Distributions of Derivatized Cardiac Glycosides

To elucidate specific molecular events and transport processes involved in cardiac glycoside sequestration, the spatially resolved analysis has to approach (sub)cellular resolution. We performed OTCD-MSI experiments with 5 μm step size of Malpighian tubules, which are multifunctional tissues involved in osmoregulation, renal excretion of nitrogenous waste, and elimination of xenobiotics and metabolic waste from the hemolymph.^48^ We note that the MSI experiments were performed without oversampling, as demonstrated in Figure S12, showing the matrix-coated sample surface after measurement and laser ablation craters with a diameter of ∼3 μm onto the penetrated tissue.

**Figure 3:**
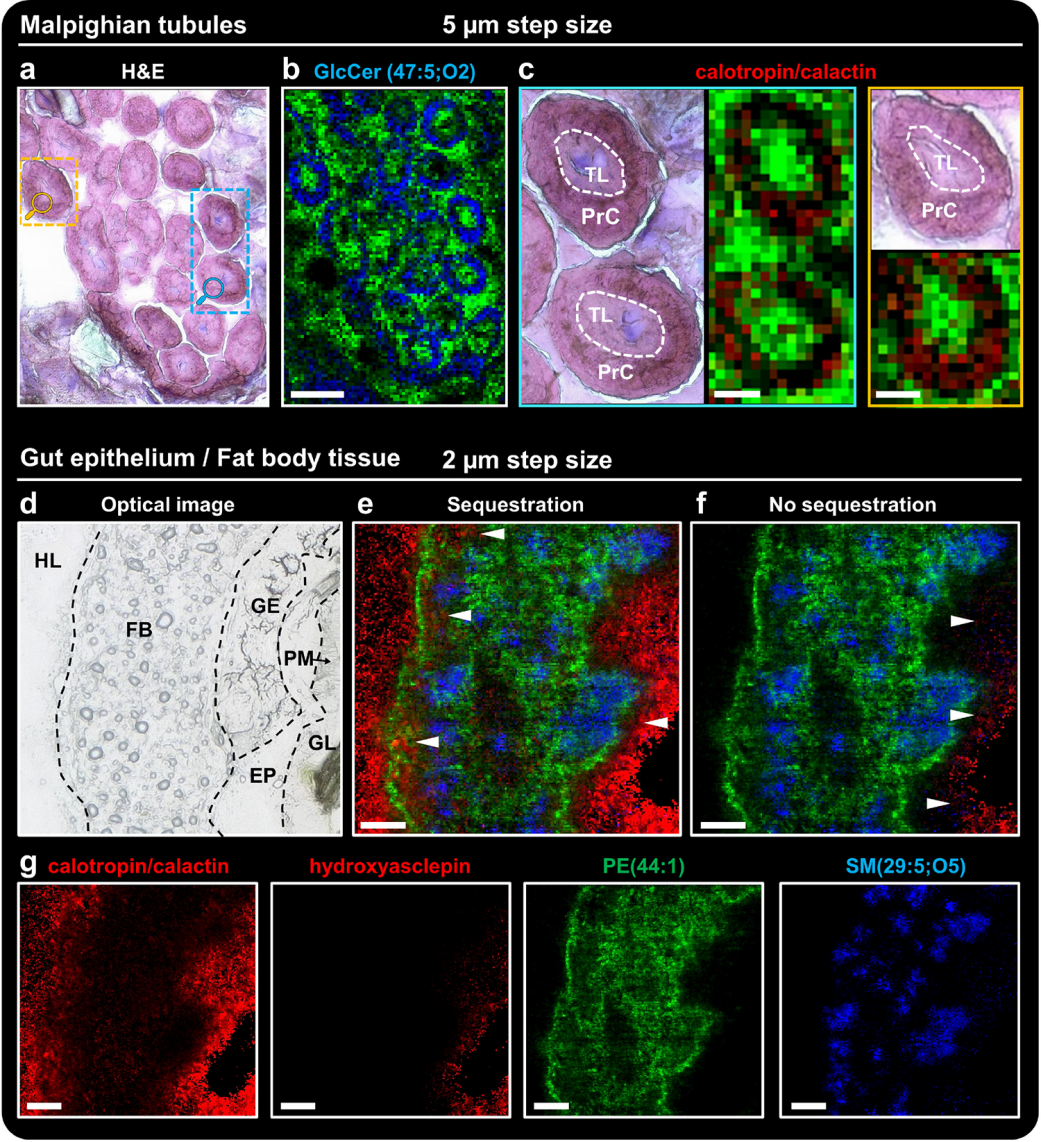
OTCD MALDI MSI of derivatized cardiac glycosides, nucleotides, and lipids at subcellular resolution in various tissue types and cells of *D. plexippus*. (a) Optical image of H&E-stained Malpighian tubules after MSI analysis. (b) Corresponding green–blue overlay image obtained with 5 μm step size showing the spatial distribution of thymidine 3′,5′-hydrogen phosphate ([M + K]^+^, *m*/*z* 343.0092, green) in the tubule lumen and GlcCer(47:5;O2) ([M + H]^+^, *m*/*z* 874.7130, blue) in the principal cell of the tubule. (c) Magnifications of the color-coded areas in (a) displaying the morphology for transverse-sectioned Malpighian tubules (PrC: principal cell; TL: tubule lumen) and their corresponding red–green overlay images obtained with 5 μm step size showing the spatial distribution of calotropin/calactin ([M + GirT]^+^, *m*/*z* 646.3699, red) and thymidine 3′,5′-hydrogen phosphate ([M + K]^+^, *m*/*z* 343.0092, green). (d) Optical image of the analyzed region of interest showing the hemolymph (HL), fat body (FB), gut epithelium (GE), ectoperitrophic space (EP), peritrophic membrane (PM), and gut lumen (GL). (e, f) Corresponding RGB overlay images obtained with 2 μm step size showing the spatial distribution of calotropin/calactin ([M + GirT]^+^, *m*/*z* 646.3699, red) for (e), hydroxyasclepin ([M + GirT]^+^, *m*/*z* 704.3754, red) for (f), and PE(44:1) ([M + H]^+^, *m*/*z* 858.6927, green) and SM(29:5;O5) ([M + Na]^+^, *m*/*z* 695.3991, blue) for (e) and (f). (g) Single ion images for the molecular compounds shown in the RGB overlays. Scale bars: (b) 100 μm, (c) 25 μm, and (e–g) 60 μm.

Figure 3a shows the optical image of H&E-stained Malpighian tubules after MSI analysis (Figure S2 for the whole longitudinal *D. plexippus* larva section and Figure S13 before MSI analysis). The morphology of transverse-sectioned tubules (Figure S14) can be reproduced by the MSI green–blue overlay image (Figure 3b) showing the spatial distribution of thymidine 3′,5′-hydrogen phosphate ([M + K]^+^ at *m*/*z* 343.0092) in green and GlcCer(47:5;O2) ([M + H]^+^ at *m*/*z* 874.7130) in blue. The nucleotide derivative was primarily located in the tubule lumen (TL) and the surrounding tissue, whereas a broad variety of lipids (Figure S15 for additional examples of different lipid classes) were exclusively detected in the principal cell (PrC).

Figure 3c displays detailed MSI results for two defined regions of interest (highlighted in Figure 3a), with the red color channel representing the spatial distribution of derivatized calotropin/calactin ([M + GirT]^+^ at *m*/*z* 646.3699). Interestingly, the cardiac glycoside was exclusively detected in the principal cell and not in the lumen of the tubules. However, the transepithelial fluid secretion to produce urine in the Malpighian tubules includes an osmotic gradient that causes water-soluble xenobiotics and metabolic waste from the hemolymph to diffuse. Thus, our OTCD MALDI MSI results suggest that cardiac glycosides are not part of the transcellular and paracellular excretion pathways and indicate that they may instead be actively transported back to the hemolymph and subsequently stored in the integument. Therefore, toxic glycosides that were already absorbed and transported through the gut epithelium are not excreted, which enhance the efficiency of the sequestration mechanism.

To demonstrate the potential of OTCD MALDI MSI for investigating the spatial organization of metabolic networks with sampling areas below 5 μm^2^, we next analyzed different physiological layers regarding cardiac glycoside uptake with 2 μm step size. We detected and spatially resolved various derivatized cardiac glycosides primarily located in the gut lumen (GL), ectoperitrophic space (EP), fat body (FB), and hemolymph (Figure 3d–g and Figure S16, red color channel). We utilized the distribution of the lipids [PE(44:1) + H]^+^ at *m*/*z* 858.6927 in green as a tissue marker for the gut epithelium and fat body and [SM(29:5;O5) + Na]^+^ at *m*/*z* 695.3991 in blue showing specific enrichments in the gut epithelium and fat body, which would most likely remain hidden with larger step sizes. The low cardiac glycoside abundance in the gut epithelium may suggest fast and efficient transport across the tissue into the hemolymph. However, different ionization efficiencies due to different sample matrix backgrounds (i.e., gut epithelium tissue and gut lumen) have to be considered. In previous studies,^39^ it was not possible to visualize cardiac glycoside distributions in the fat body of the larvae. However, we observed the accumulation of calotropin/calactin, calotoxin/hydroxycalotropin/hydroxycalactin, and the precursor calotropagenin in the fat body with an increased accumulation in the outer layer of the fat tissue (Figure 3e and Figure S16a). Notably, this observation was not made for other derivatized metabolites with similar polarity (e.g., futalosine derivative; Figure S16b), thus suggesting that this specific pattern along with other (sub)cellular cardiac glycoside distributions is not caused by analyte diffusion effects during sample preparation. Figure 3f shows the spatial distribution for hydroxyasclepin, which is not sequestered by *D. plexippus* (as determined in the previous MSI experiment). Instead, hydroxyasclepin, which belongs to one of the most abundant cardiac glycosides in *A. curassavica*, was exclusively detected in the gut lumen and ectoperitrophic space with similar intensity to calotropin/calactin but not located in the gut epithelium tissue (despite being in direct contact). Thus, our MSI data demonstrate that the peritrophic membrane (PM), which was shown to restrict the cardenolide digitoxin to the gut lumen of locusts,^49^ has no function regarding the selectivity of cardiac glycoside sequestration in our system.

## Conclusions

Our workflow combines novel computational methods for *in silico* annotation, classifying chemical compounds and generating molecular networks based on LC–MS bulk analysis with high-resolution OTCD MALDI MSI in a robust way to comprehensively analyze metabolic networks in the spatial context of tissues and cells. Utilizing plant toxin sequestration in *D. plexippus* as a model system, we were able to structurally characterize and identify 32 different steroidal glycosides in the host plant *A. curassavica*. To the best of our knowledge, this is the highest number of detected cardiac glycosides for this milkweed species, thereby demonstrating the enormous potential of computational metabolomics approaches to decompose metabolic networks into compound classes and molecule annotations. However, no available *in silico* molecular fingerprint-based annotation method can distinguish between correct and incorrect annotations, making manual evaluation necessary. Our covalent charge-tagging approach using the GirT reagent substantially improved the sensitivity and enabled the spatial visualization of carbonyl-containing cardiac glycosides in the fat body, gut epithelium, Malpighian tubules, and epidermal integument cells of *D. plexippus* with pixel sizes of 2, 5, and 25 μm. In this context, the optimized OTCD sample preparation protocol preserved spatial integrity to ensure that the effective lateral resolution was defined by the laser spot size rather than the analyte diffusion radius. Although primarily demonstrated here for carbonyl-containing steroidal glycosides, many functional groups (phenols,^50,51^ thiols,^52^ amines,^53,54^ carboxylic acids,^55^ phosphate monoesters,^56^ and alkenes^57,58^) can be targeted with different OTCD reagents to achieve increased ion yields and to force specific fragmentation patterns. Thus, our generic workflow represents a customizable and expandable method and can readily be applied to a wide range of spatially resolved small-molecule studies in the fields of chemistry, biology, and medicine.

## Supporting Information Available

More detailed descriptions regarding experimental parameters and data analysis (Supplementary Notes 1–6); overview for all annotated cardiac glycosides (Table S1); experimental design of this study (Figure S1); optical image of final instar longitudinal *D. plexippus* section (Figure S2); comprehensive FBMN results (Figure S3); LC–MS^2^ spectra for calotropin and frugoside highlighting different fragmentation pathways (Figure S4); additional results for OTCD method development (Figures S5–S9); box plots for *in silico* quantification of MSI data (Figure S10), MS spectra for control and OTCD MSI obtained at *D. plexippus* integument (Figure S11); optical images of *D. plexippus* Malpighian tubules before OTCD and matrix application and after MSI and H&E staining (Figures S12 and S13); simplified scheme showing Malpighian tubule morphology (Figure S14); OTCD MALDI MSI results showing subcellular lipid distributions in Malpighian tubules (Figure S15); OTCD MALDI MSI results obtained with 2 μm step size showing various derivatized metabolites (Figure S16); and LC–MS^2^ spectra for all detected cardiac glycosides including fragmentation pathways (Supplementary Data 1) (PDF)
