# Supplementary material for: Molecular Networking and On-Tissue Chemical Derivatization for Enhanced Identification and Visualization of Steroid Glycosides by MALDI Mass Spectrometry Imaging
Source: Anal Chem. 2022 Nov 8;94(46):15971–9. doi: 10.1021/acs.analchem.2c02694 (PMC9685589; doi:10.1021/acs.analchem.2c02694)

Supplementary Information

Molecular Networking and On-Tissue Chemical Derivatization for Enhanced Identification and Visualization of Steroid Glycosides by MALDI Mass Spectrometry Imaging

Domenic Dreisbach[1], Sven Heiles[1,2,3], Dhaka R. Bhandari[1], Georg Petschenka[4], Bernhard Spengler*[1]

[1]Institute for Inorganic and Analytical Chemistry, Justus Liebig University Giessen, Heinrich-Buff-Ring 17, 35392 Giessen, Germany

[2]Leibniz Institute for Analytical Sciences, ISAS – e.V., Otto-Hahn-Straße 6b, 44139 Dortmund, Germany

[3]Lipidomics, Faculty of Chemistry, University of Duisburg-Essen, Universitätsstraße 5, 45141 Essen, Germany

[4]Institute of Phytomedicine, University of Hohenheim, Otto-Sander-Straße 5, 70599 Stuttgart, Germany

* corresponding author: bernhard.spengler@anorg.chemie.uni-giessen.de

Table of Content

**Supplementary Protocol 1.**	**S3**
**Supplementary Note 1.**	**S3**
**Supplementary Note 2.**	**S3**
**Supplementary Note 3.**	**S4**
**Supplementary Note 4.**	**S4**
**Supplementary Note 5.**	**S5**
**Supplementary Note 6.**	**S5**
**Supplementary Table 1.**	**S6**
**Figure S1.**	**S7**
**Figure S2.**	**S8**
**Figure S3.**	**S9**
**Figure S4.**	**S11**
**Figure S5.**	**S12**
**Figure S6.**	**S13**
**Figure S7.**	**S14**
**Figure S8.**	**S15**
**Figure S9.**	**S16**
**Figure S10.**	**S17**
**Figure S11.**	**S18**
**Figure S12.**	**S19**
**Figure S13.**	**S20**
**Figure S14.**	**S21**
**Figure S15.**	**S22**
**Figure S16.**	**S23**
**Supplementary Data 1.**	**S24**

Supplementary Protocol 1: H&E staining protocol

1. Dehydration in ethanol and set to demineralized water

100 % ethanol:	2 min
70% ethanol:	2 min
40% ethanol:	2 min
Demineralized water:	2 min

2. Hematoxylin: 12 min
3. Washing steps

Tap water:	10 min
Demineralized water:	5 min

4. 1% aqueous eosin Y solution: 1 min
5. Differentiation in ethanol

Demineralized water:	2 min
40% ethanol:	2 min
70% ethanol:	2 min
100 % ethanol:	2 min

6. Xylol
7. Eukitt fixation

Supplementary Note 1: Sample preparation for LC-MS

A. curassavica leaf samples were harvested, immediately freeze-dried in liquid nitrogen, ground to a fine powder and subsequently extracted for LC-MS^2 experiments. An amount of 50 mg of dry leaf powder was transferred into 2 mL screw-cap vials (Sarstedt, Nümbrecht, Germany) and after the addition of 900 mg zirconia beads (Roth, Karlsruhe, Germany), samples were homogenized in a Fast-Prep-24 instrument (MP Biomedicals, Germany) for two 45-sec cycles at a speed of 6.5 m/sec. Subsequently, samples were centrifuged at 16,100 rpm and supernatants were transferred into fresh vials. After evaporating samples to dryness under a stream of N_2, dry residues were dissolved in 1 mL methanol by agitation in the Fast-Prep-24 instrument (without the addition of beads). Before LC-MS analysis, samples were filtered via Rotilabo®-syringe filters (nylon, 0.45 µm, Roth, Karlsruhe, Germany) and diluted (1:20) using MeOH.

Supplementary Note 2: Experimental parameters for MSI analysis

All MSI measurements were conducted in the positive-ion mode in mass-to-charge-number (*m*/*z*) range of 250 to 1000 at a mass resolution of 240,000 for Q Exactive HF (120,000 for Q

Exactive) at *m*/*z* 200. Internal lock-mass calibration was performed by using the DHB matrix cluster ion signal at *m*/*z* 716.12461 ($[5DHB{-}4H_2O{+}NH_4]^+$), resulting in a high mass accuracy of $\pm$1 ppm. For desorption/ionization, 50 laser pulses per pixel at a wavelength of 343 nm were focused perpendicularly to the sample surface. The step size of the XYZ sample stage was set to the desired pixel size. The full-pixel mode was used for MALDI MSI experiments conducted with 25 µm step size. In full-pixel mode, the pixel area is ablated by multiple laser pulses by a meandering movement to improve the ion signal intensities of the MSI experiment. The scan speed for all MSI experiments was 1.6 pixel/s. The acceleration voltage was set to 3 kV. The ion injection time was set to 500 ms. The capillary temperature was 250 °C, and the S-lens level was set to 100 arbitrary units.

Supplementary Note 3: Experimental parameters for HPLC-MS analysis

The injection volume was 15 µL, and the column compartment was set to 50 °C. Mobile phase A was water (0.1 % FA). Mobile phase B was acetonitrile (0.1 % FA). A flow rate of 0.5 mL/min applying the following gradient: 0–2 min, 10% B; 2–20 min, 20–70% B; 20–25 min, 70–95% B; 25–30 min, 95% B; 30–35 min, 95–10% B. The mass spectrometer was operated in positive-ion mode in a mass-to-charge-number (*m*/*z*) range of 250 to 1000 at a mass resolution of 240,000 at *m*/*z* 200. Data-dependent acquisition (DDA) for MS^2 spectra was performed. Up to 7 MS^2 spectra per MS^1 survey scan were recorded. The maximum ion injection time for MS^2 scans was set to 100 ms with an AGC target of 1E5 ions and a minimum of 5% AGC. The MS^2 precursor isolation window was set to $\Delta(m/z) = \pm 1$. Normalized collision energy (NCE) of 25 % was used for fragmentation with $z = 1$ as the default charge state. Dynamic precursor exclusion was set to 6s. The following HESI-source parameters were applied: spray voltage (+), 3.5 kV; capillary temperature, 300 °C; sheath gas flow rate, 35 psi; aux gas flowrate, 12 psi; aux gas heater temperature, 150 °C.

Supplementary Note 4: LC-MS data pre-processing using MZmine 2

The mzXML files were imported and following parameters were applied: an initial threshold of 1E6 for MS^1 spectra and 1E4 for MS^2 spectra was used. For feature-detection, the ADAP chromatogram builder was used with a minimum signal intensity and group intensity threshold of 3E6 and with $\pm$ 3 ppm mass tolerance. A minimum mass appearance over 5 consecutive scans was set. The extracted ion chromatograms were deconvoluted using the local minimum search algorithm with a chromatographic threshold of 10%, search minimum in RT range of 0.3 min, minimum relative height of 10%, minimum absolute height of 3E6, minimum ratio of peak top/edge 0.5 and peak duration between 0.02 min and 3 min. For MS^1-MS^2 pairing, a

mass range of 0.01 Da for median m/z centre calculation and RT range of 0.2 min was used. Isotope signals were grouped with m/z tolerance of $\pm$ 3 ppm and RT tolerance of 0.1 min.

Supplementary Note 5: Feature-based molecular networking from the GNPS analysis infrastructure

The .mgf files were uploaded into the GNPS ecosystem and for FBMN following parameters were applied: precursor ion mass tolerance: 0.01 Da, fragment ion mass tolerance: 0.01 Da. The minimum cosine score between a pair of MS2 spectra in order to form an edge in the molecular network was 0.7. The maximum amount of neighbour nodes from a single node was 10. The minimum number of fragment ions that were shared between pairs of related MS^2 spectra was 5. The maximum precursor ion mass difference between two nodes was 600 Da. For spectral library annotations, the MS^2 spectra contained a minimum number of 6 matched fragment ions. Additionally, the minimum cosine score after spectral matching of experimental MS^2 spectra with spectral library MS^2 spectra was 0.7. The molecular networking results were imported into Cytoscape (v.3.8.0). For optimal visualization, H^+-adducts were exclusively shown.

Supplementary Note 6: Method development for high-resolution OTCD MALDI MSI of cardiac glycosides

We optimized the sample preparation protocol to achieve high sensitivity while retaining spatial information. In summary, the solvent composition of methanol/water (7:3) v/v enabled high GirT-concentration (15 mg/mL), which subsequently allowed us to use a low spray volume (35 µL) combined with a low flowrate (7 µl/min) to prevent analyte delocalization and washing effects (as shown in Figure S5 for 50 µL spray volume and 10 µL/min flow rate). Between OTCD and matrix application, the sample tissue was transferred into a desiccator for two hours at room temperature to increase reaction yield (Figure S6) and to prevent spatial artefacts due to hygroscopic properties of the GirT reagent. Any further incubation step that included increased humidity and temperature resulted in analyte delocalization and washing effects (Figure S7) and was thus avoided. Next, OTCD was quenched by matrix application, which also provides chemical preservation and inhibits oxidation of derivatized cardiac glycosides (Figure S6). Using an ultrafine pneumatic spraying protocol for DHB as a matrix with methanol/water (1:1) v/v as solvent showed excellent results regarding homogenous matrix crystallization (crystal sizes $\leq$ 10 µm) for different surface characteristics of *D. plexippus* tissue sections, including fat body tissue, digested plant material and integument (Figure S8 and Figure S9 for comparison with DHB matrix layer without OTCD).

Supplementary Table 1. Overview of the cardiac glycoside composition in *A. curassavica*, determined via LC-MS-based feature-based molecular networking and *in silico* molecular characterization.

m/z (exp.)	mass error (ppm)	sum formula	RT (min)	annotation
375.2530	1.2	$C_{23}H_{34}O_4$	12.91	digitoxigenin
405.2280	1.1	$C_{23}H_{34}O_4$	5.24	calotropagenin
407.2433	1.3	$C_{23}H_{34}O_6$	4.90	strophanthidol
505.3165	1.0	$C_{29}H_{44}O_7$	11.07	digitoxigenin digitoxoside
519.2879	1.1	$C_{29}H_{42}O_8$	12.20	strobosid
531.2587	1.2	$C_{29}H_{38}O_9$	10.42	uscharidin
533.2742	1.5	$C_{29}H_{40}O_9$	8.47	calactin
533.2742	1.5	$C_{29}H_{40}O_9$	9.18	calotropin
535.2902	0.9	$C_{29}H_{42}O_9$	8.26	gofruside
537.3060	0.7	$C_{29}H_{44}O_9$	7.94	frugoside
547.2541	0.8	$C_{29}H_{38}O_{10}$	9.53	hydroxyuscharidin
549.2692	1.0	$C_{29}H_{40}O_{10}$	6.20	calactinic acid
549.2692	1.0	$C_{29}H_{40}O_{10}$	7.24	calotoxin
549.2693	1.0	$C_{29}H_{40}O_{10}$	8.38	hydroxycalactin
549.2693	1.0	$C_{29}H_{40}O_{10}$	9.27	hydroxycalotropin
551.2860	0.6	$C_{29}H_{42}O_{10}$	6.33	corglycon
553.3015	0.7	$C_{29}H_{44}O_{10}$	6.16	periplogenin glycoside
561.3063	0.9	$C_{31}H_{44}O_9$	11.95	gomphoside-acetate
575.2855	0.5	$C_{31}H_{42}O_{10}$	10.70	asclepin
579.2805	1.1	$C_{30}H_{42}O_{11}$	8.14	hydroxycalactinic acid methylester
588.2627	0.8	$C_{31}H_{41}NO_8S$	11.56	uscharin
590.2791	0.5	$C_{31}H_{43}NO_8S$	10.92	voruscharin
591.2798	1.1	$C_{31}H_{42}O_{11}$	8.19	acetoxycalotropin
591.2803	0.9	$C_{31}H_{42}O_{11}$	8.56	hydroxyasclepin
604.2573	1.1	$C_{31}H_{41}NO_9S$	7.76	hydroxyuscharin
606.2729	0.9	$C_{31}H_{43}NO_9S$	10.78	hydroxyvoruscharin
619.3115	0.8	$C_{33}H_{46}O_{11}$	11.37	diacetylgofruside
633.2902	1.0	$C_{33}H_{44}O_{12}$	10.43	acetoxyasclepin
635.3795	0.7	$C_{35}H_{54}O_{10}$	12.01	digitoxin bisdigitoxoside
697.3430	0.9	$C_{35}H_{52}O_{14}$	7.13	cannogenin diglycoside
699.3591	0.6	$C_{35}H_{54}O_{14}$	6.83	antiaroside B
711.3220	0.8	$C_{35}H_{50}O_{16}$	5.91	calotoxin-glycoside

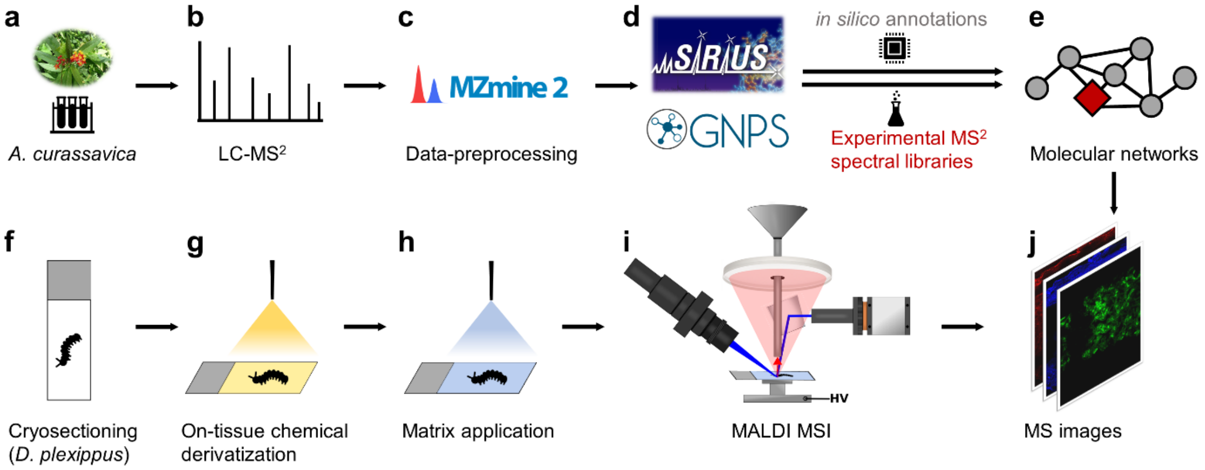

Figure S1. Experimental design for the FBMN-based annotation and structural characterization of cardiac glycosides in *A. curassavica*, followed by *in situ* visualization of metabolic networks related to sequestration in *D. plexippus* using OTCD MALDI MSI. (**a**) Metabolite extraction for *A. curassavica* leaf samples. (**b**) Performing non-targeted LC-MS^2 experiments. (**c**) LC-MS feature detection and data-preprocessing using MZmine 2. (**d**) FBMN workflow from the GNPS ecosystem in combination with SIRIUS for increased annotation rate, compound classification and structural elucidation. (**e**) Visualizing molecular networking results using Cytoscape and generate internal database to facilitate metabolite identification for subsequent MSI experiments. (**f**) Cryo-sectioning of last instar *D. plexippus* larvae. (**g**) Optimized pneumatic spraying protocol for OTCD using GirT reagent. (**h**) Optimized pneumatic spraying protocol for DHB matrix solution. (**i**) Performing high-resolution MALDI MSI experiments. (**j**) Identification of derivatized metabolites and generating the respective MS ion images.

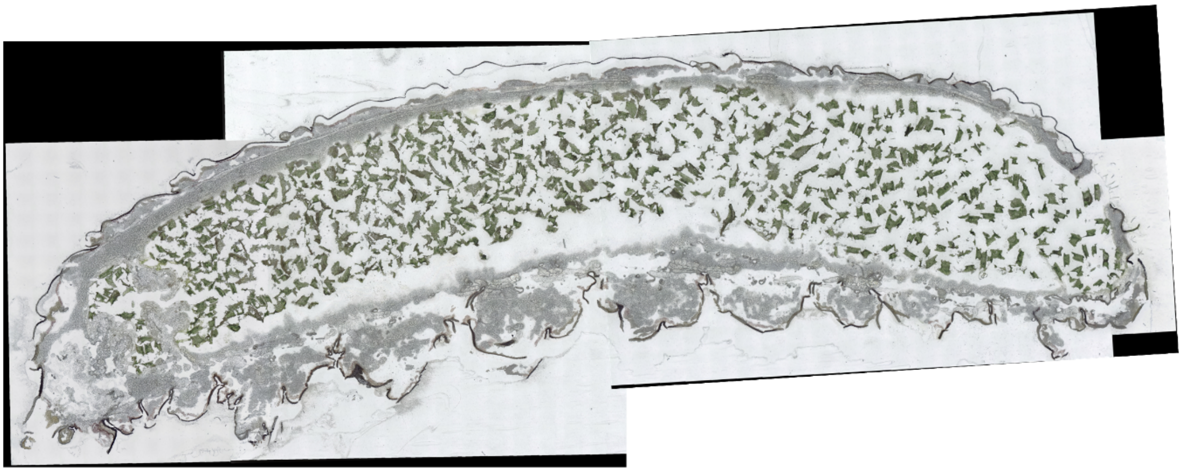

Figure S2. Optical image of longitudinal last instar *D. plexippus* caterpillar section.

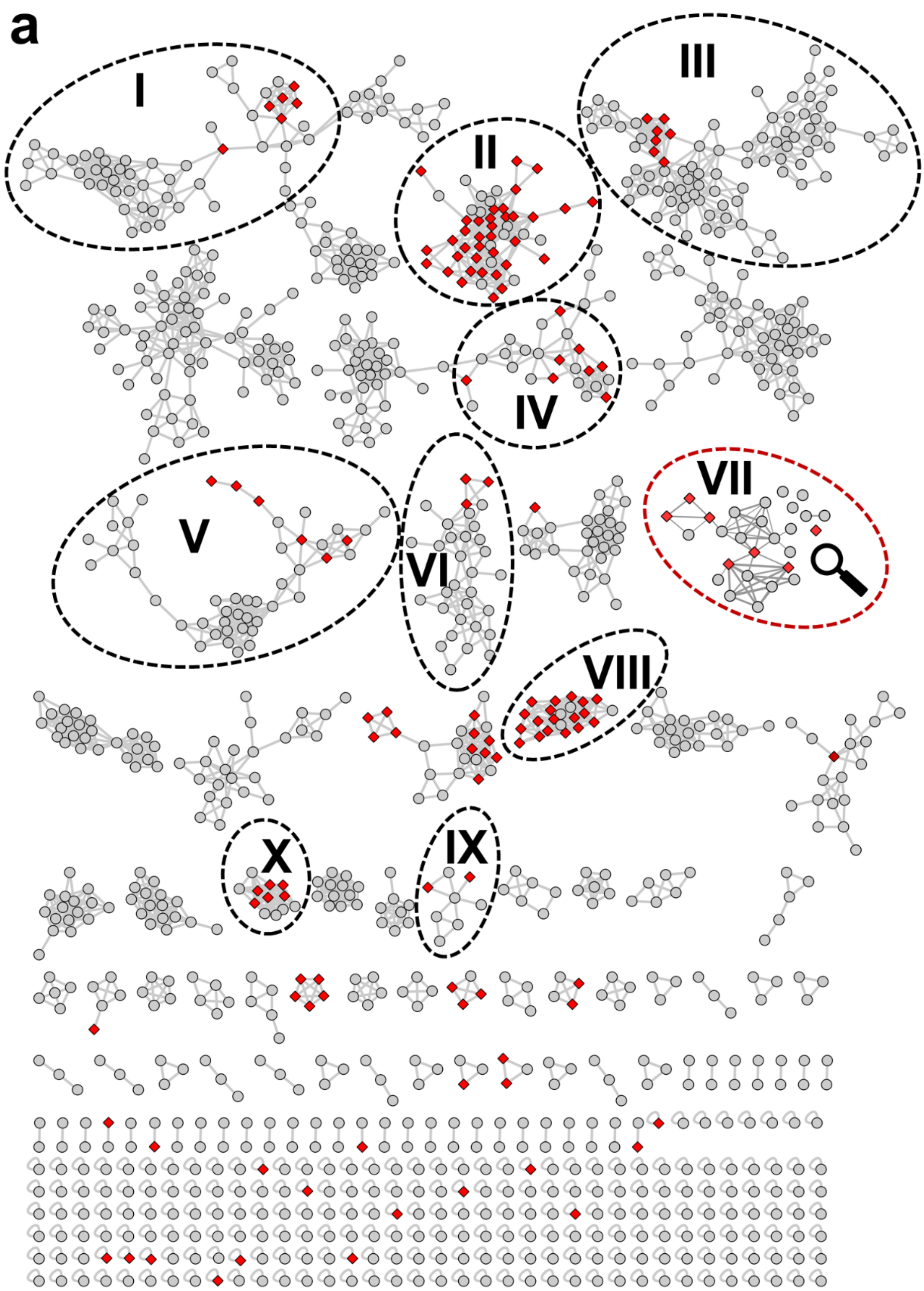

Figure S3. Comprehensive FBMN results consisting of 1175 mass spectral nodes, that were organized into 89 independent molecular families. In total, 145 spectral library hits (red nodes) were obtained allowing to classify the corresponding molecular networks. For instance, the molecular networks I to III (MN I-III) were composed of fatty acids, phosphatidylcholines (PC), sphingomyelins (SM) and arachidonoylthio-PCs; MN IV contains flavonoid glycosides, MN V contains phosphatidylethanolamines (PE), MN VI contains carotenoids, MN VII contains

cardiac glycosides, MN VIII contains flavonoids, MN IX contains polysaccharides and MN X contains isoflavonoids.

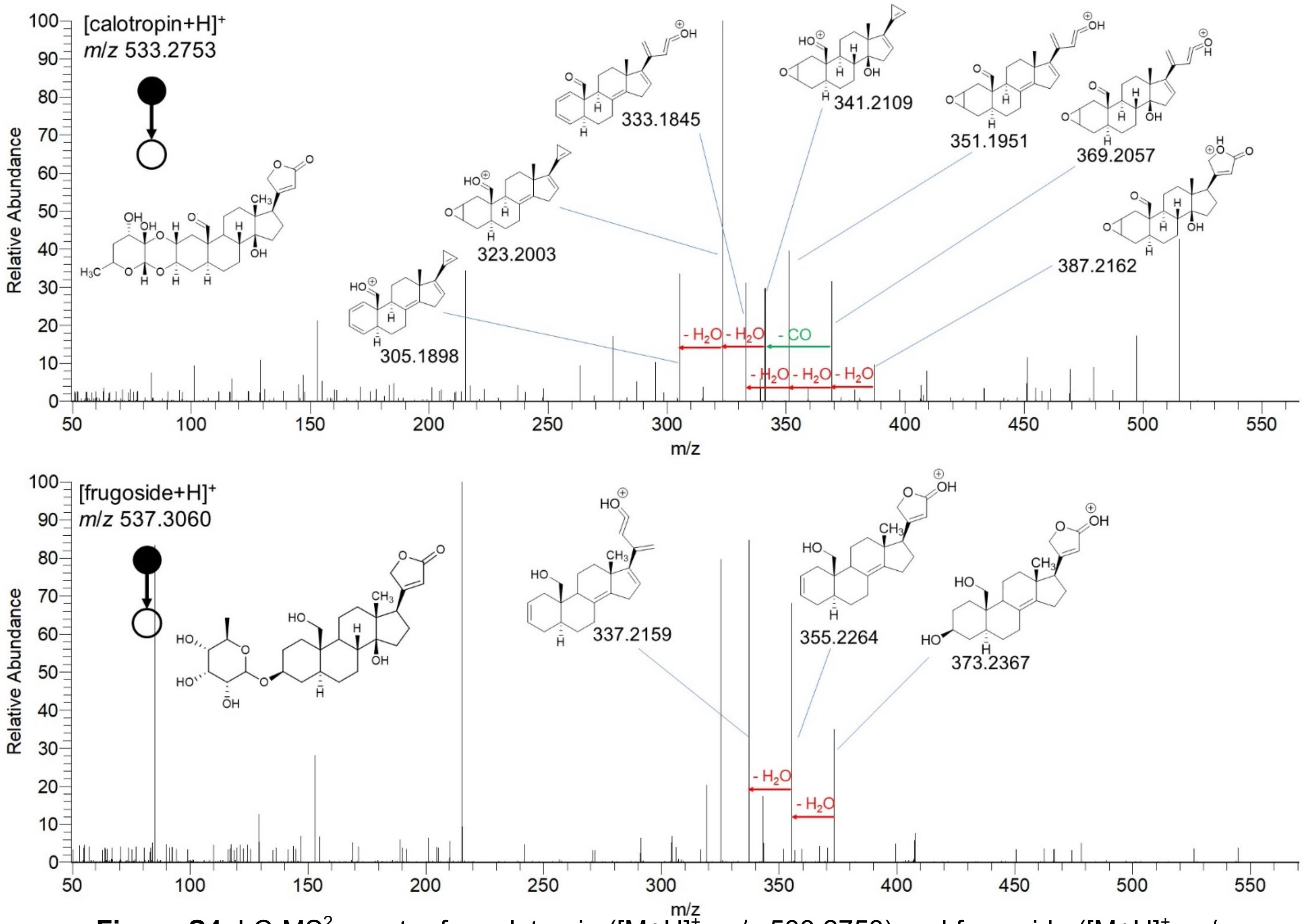

Figure S4. LC-MS[2] spectra for calotropin ($[M+H]^+$, *m*/*z* 533.2753) and frugoside ($[M+H]^+$, *m*/*z* 537.3060) highlighting the differences regarding characteristic fragments. Thus, frugoside was not part of the molecular network, which is based on the characteristic fragmentation pathway of calotropin, calactin and asclepin.

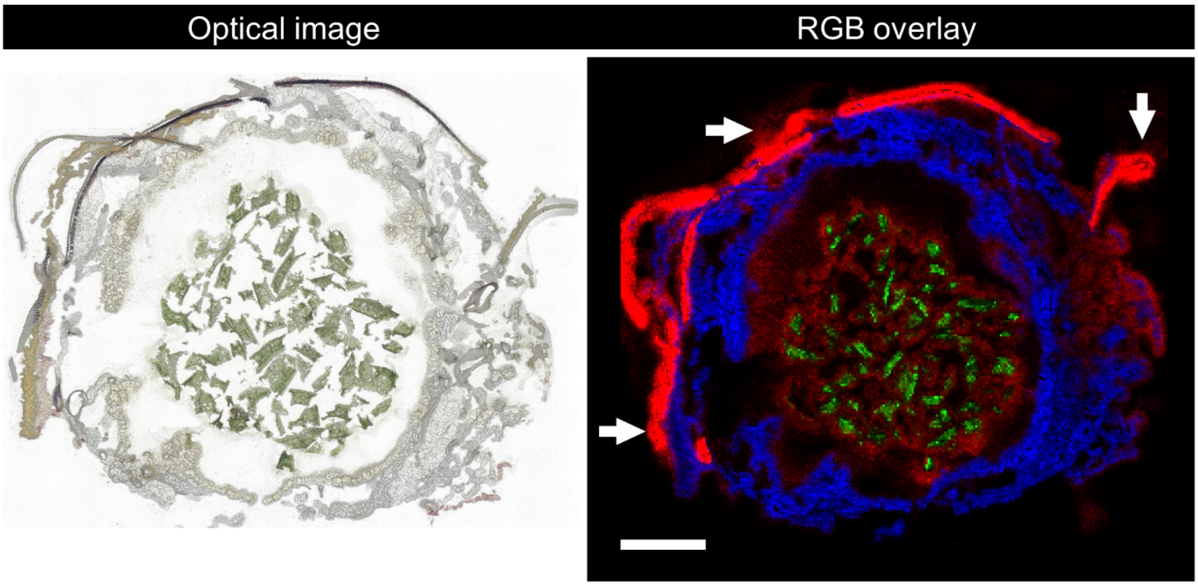

Figure S5. OTCD MALDI MSI of derivatized cardiac glycosides in *D. plexippus*. After GirT-reagent application (10 mg/ml in MeOH/water 7:3 v/v, 50 µl spray volume, 10 µl/min flowrate), the sample was transferred in a desiccator for 2 hours. (**a**) Optical image of transversal *D. plexippus* section before OTCD and matrix application. (**b**) Corresponding RGB overlay image obtained with 25 µm step size showing the spatial distribution of calotropin/calactin in red ($[M+GirT]^+$ at *m*/*z* 646.3699), pheophytin a ($[M+K]^+$ at *m*/*z* 909.5291) in green and PS(18:1/18:2) ($[M+K]^+$ at *m*/*z* 810.5046) in blue. The extensive cardiac glycoside distribution at the integument indicates washing-out effects and analyte delocalization. Scale bar, 1 mm.

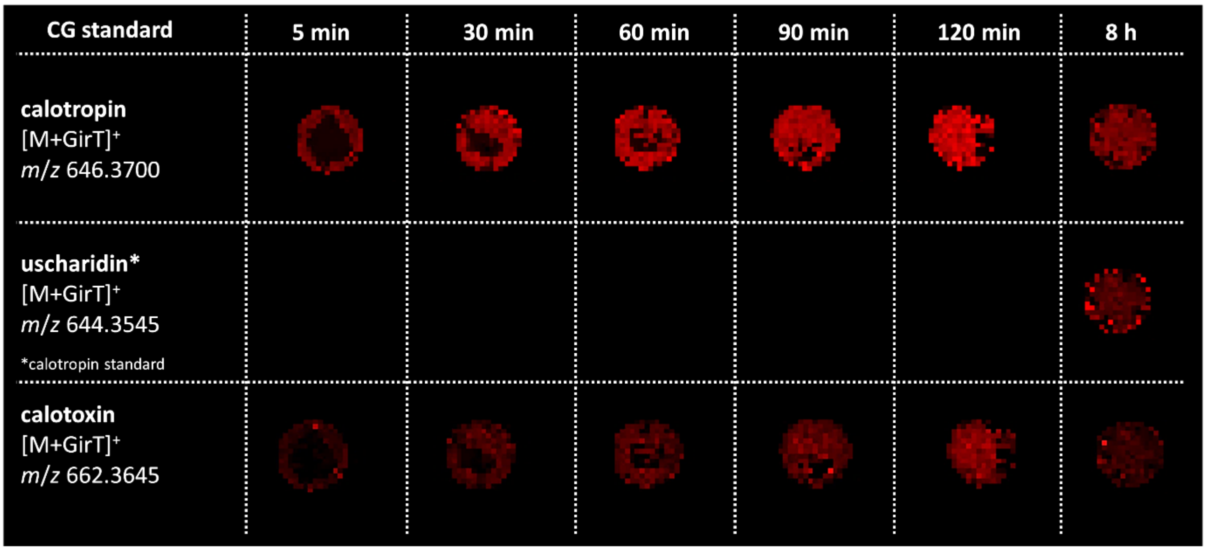

Figure S6. Dried-droplet MALDI MSI experiments of two derivatized cardiac glycoside standards (calotropin and calotoxin). A volume of 1 µL cardiac glycoside standard was spotted into reaction holes of a PTFE-coated glass slide. Subsequently, 1 µL of GirT-reagent (15 mg/mL) was added. After specific time intervals, the reaction was quenched by adding 1 µL of DHB-matrix solution (30 mg/mL). The highest reaction yield was obtained after 120 minutes of incubation. After 8 hours, uscharidin was detected for the calotropin standard measurement indicating oxidation of the respective species. Note that all reaction holes were measured simultaneously after the longest incubation time interval (8 hours). Thus, our data suggests that the chemical reaction is successfully quenched by adding the DHB matrix solution, which subsequently provides chemical preservation and inhibits the oxidation of derivatized cardiac glycosides (see time incubation intervals of ≤ 2 hours).

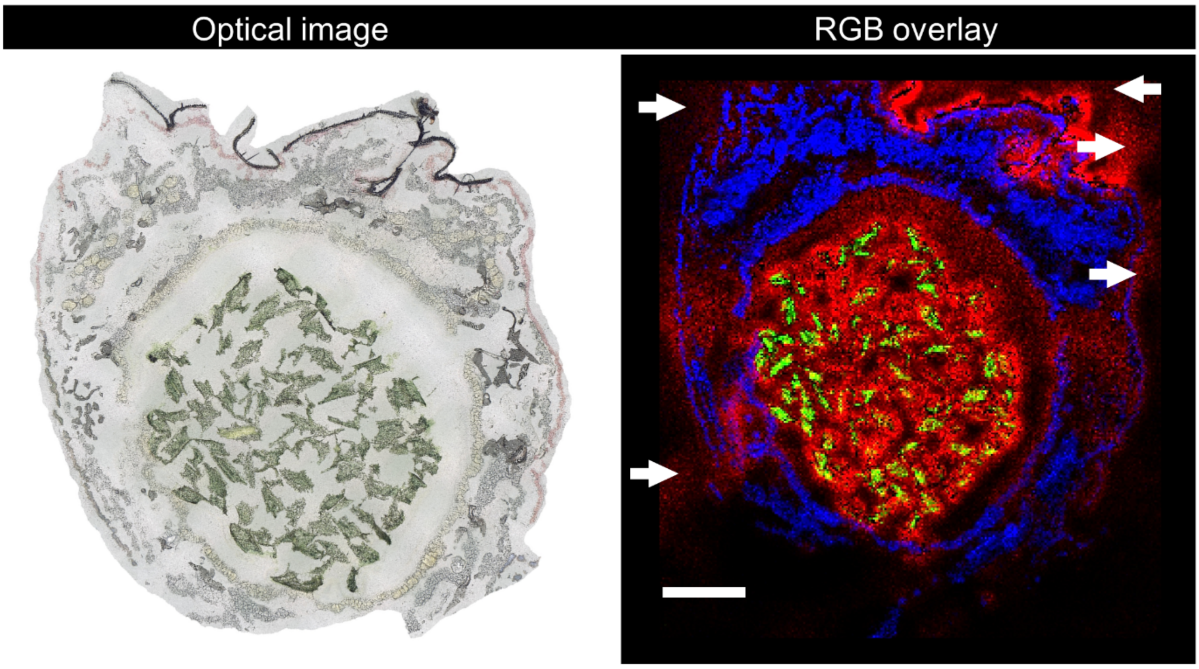

Figure S7. OTCD MALDI MSI of derivatized cardiac glycosides in *D. plexippus*. After GirT-reagent application (10 mg/ml in MeOH/water 7:3 v/v, 50 µL spray volume, 10 µL/min flowrate), the sample was incubated in a cell culture incubator (38°C, 100% relative humidity) for 2 hours. (**a**) Optical image of transversal *D. plexippus* section before OTCD and matrix application. (**b**) Corresponding RGB overlay image obtained with 25 µm step size showing the spatial distribution of calotropin/calactin in red ($[M+GirT]^+$ at *m*/*z* 646.3699), pheophytin a ($[M+K]^+$ at *m*/*z* 909.5291) in green and PS(18:1/18:2) ($[M+K]^+$ at *m*/*z* 810.5046) in blue. The extensive and leaked-out cardiac glycoside distribution at the integument indicates washing-out effects and analyte delocalization. Scale bar, 1 mm.

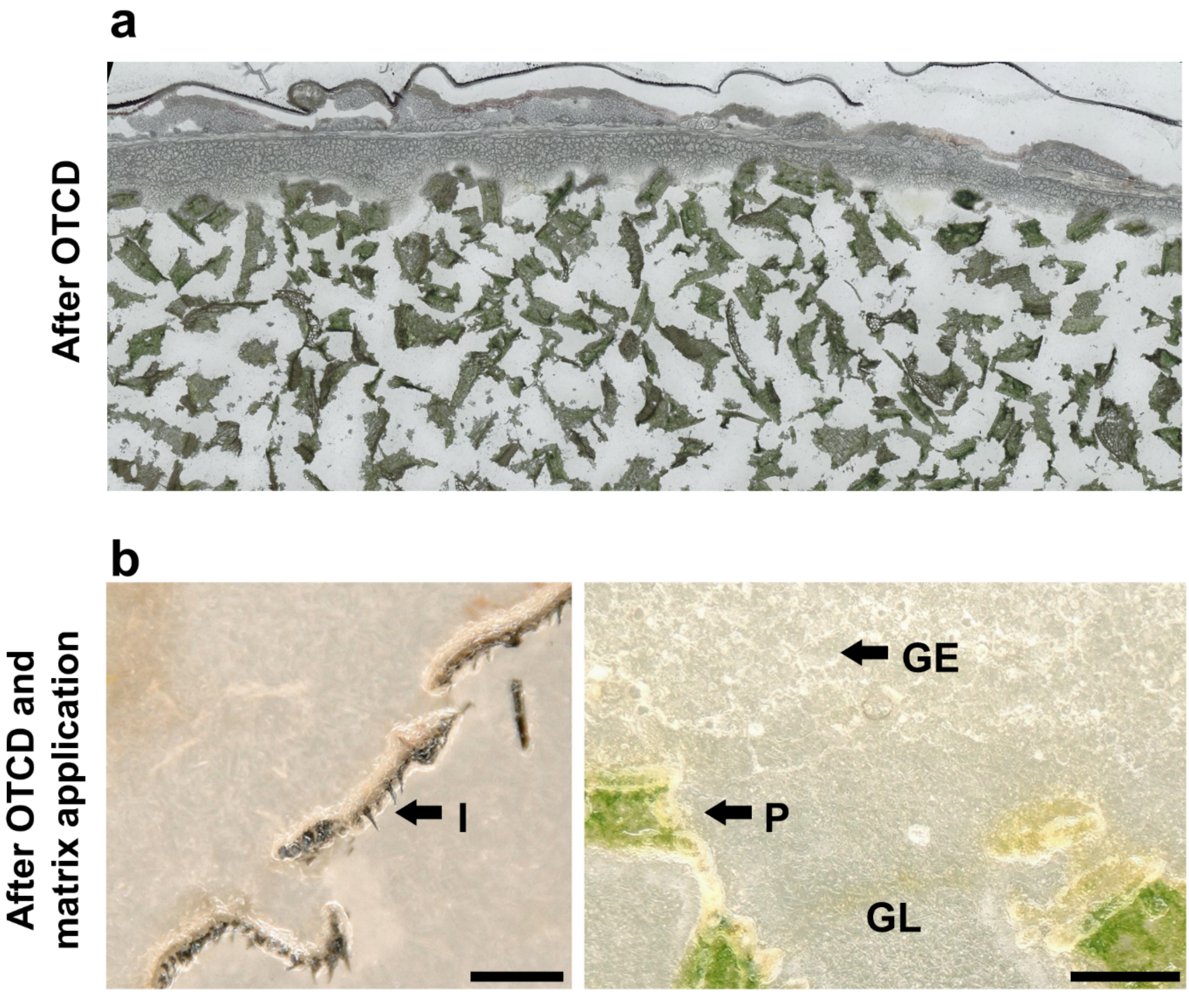

Figure S8. Optical images of longitudinal *D. plexippus* section (**a**) after OTCD and (b) after OTCD and matrix application. The optimized sample preparation protocol for OTCD (15 mg/ml GirT in MeOH/water 7:3 v/v, 35 µL spray volume, 7 µL/min flowrate) and matrix application (30 mg/ml DHB in MeOH/water 1:1 v/v, 100 µL spray volume, 5 µL/min flowrate) provides a homogenous matrix layer with crystal sizes of $\leq$ 10 µm, which is demonstrated for different surface characteristics including integument (I) plant material (P), gut lumen (GL) and gut epithelium (GE). Scale bars, 100 µm.

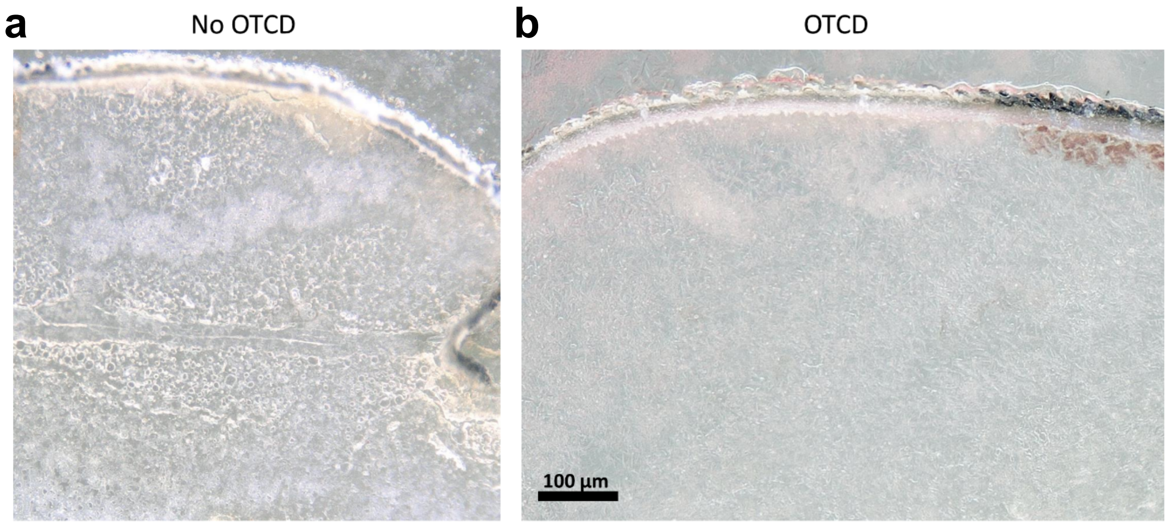

Figure S9. Optical images of longitudinal *D. plexippus* section for matrix layer comparison of (**a**) control experiment (conventional MALDI MSI without OTCD) and (**b**) OTCD MALDI MSI.

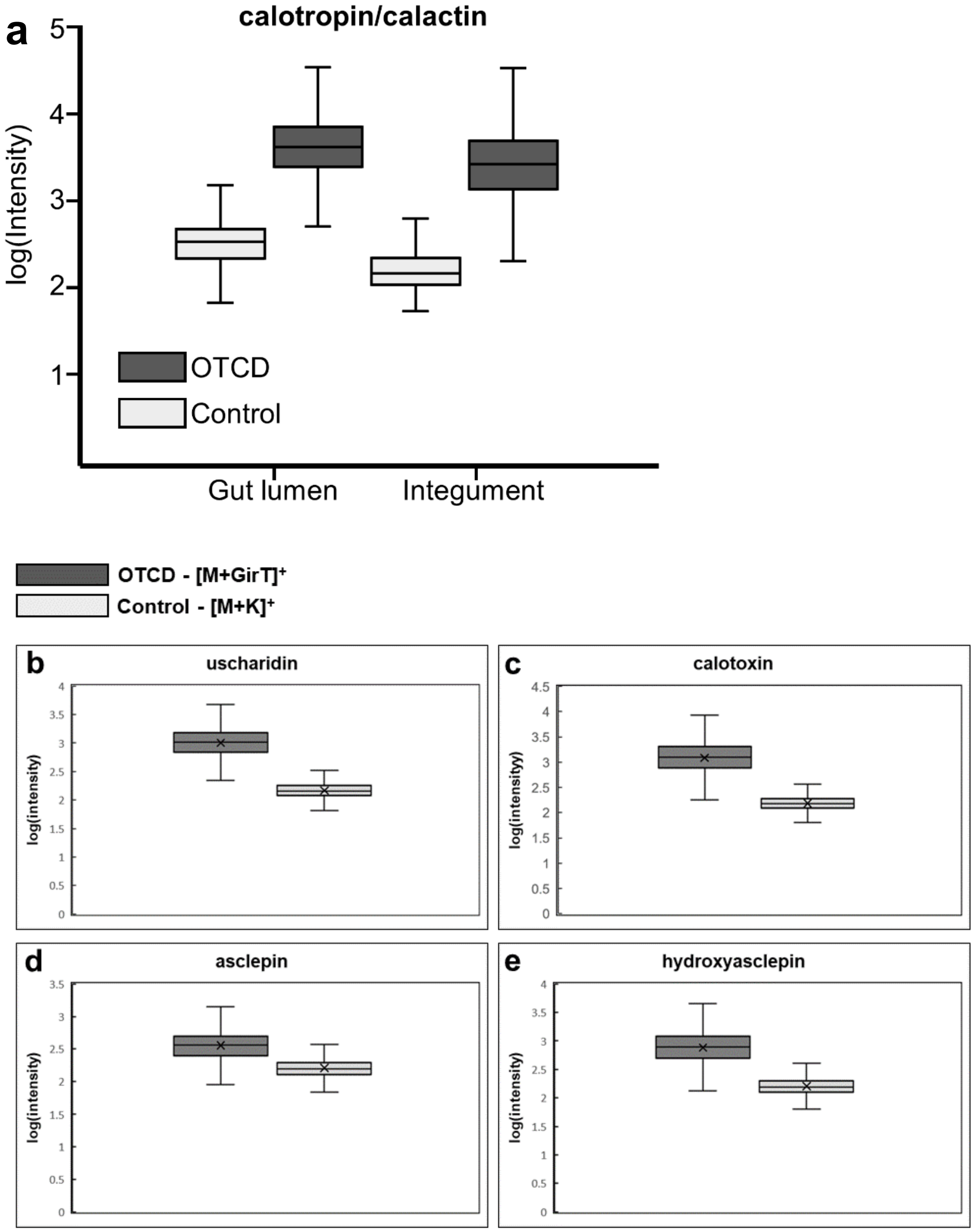

Figure S10. Box plots showing *in silico* extracted ion intensities of cardiac glycosides for OTCD and control MALDI MSI. Utilizing OTCD, cardiac glycoside ion intensities were increased by (**a**) 16-fold for calotropin/calactin (**b**) 13.9-fold for uscharidin, (**c**) 14.3-fold for calotoxin, (**d**) 11.5-fold for asclepin and (**e**) 13.1-fold for hydroxyasclepin.

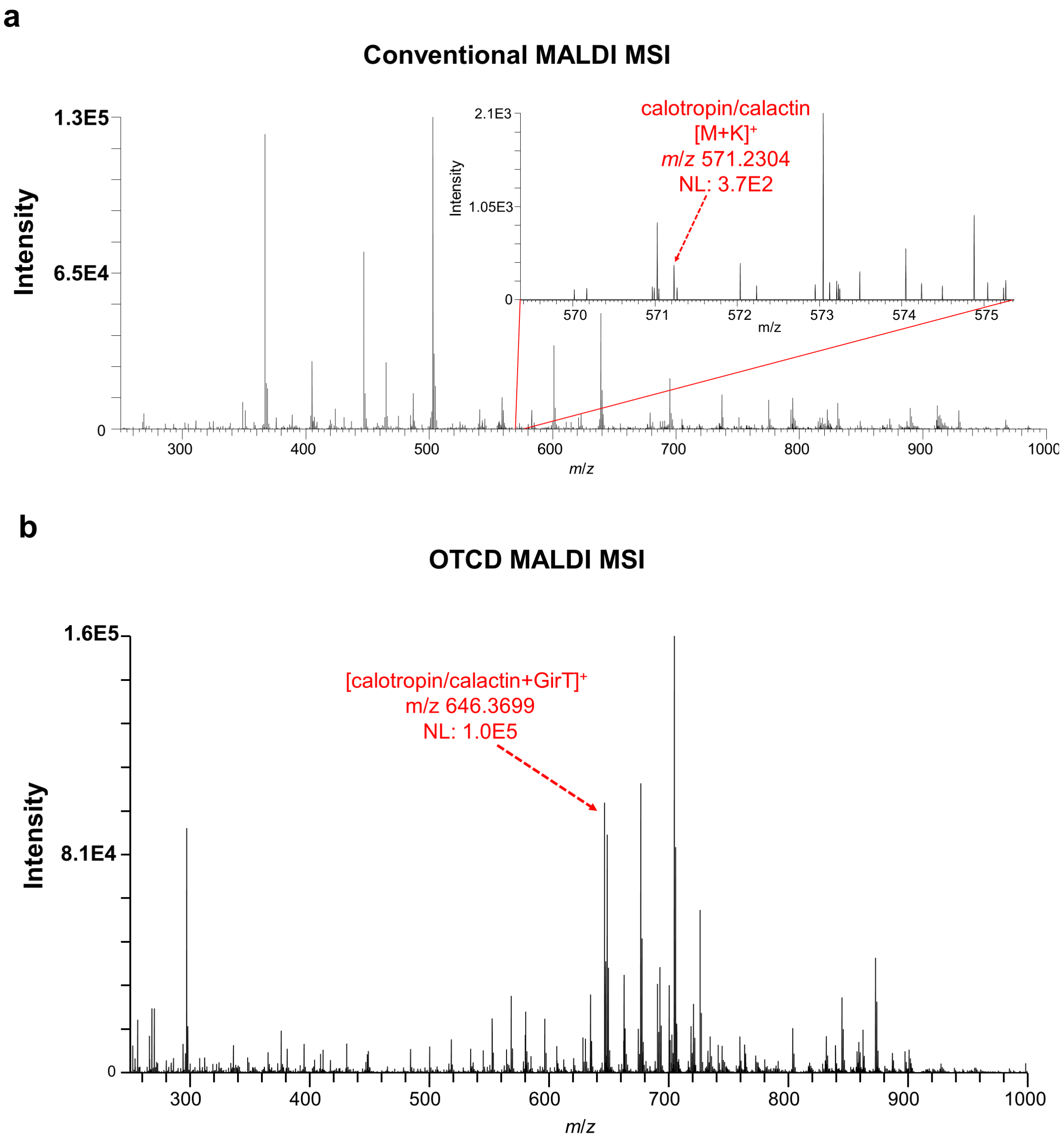

Figure S11. Representative MALDI MSI spectrum for (**a**) conventional MALDI (control) and (**b**) OTCD MALDI MSI (MSI data shown in Figure 2b), acquired from a single pixel at *D. plexippus* integument. For the control experiment, the ion signal of $[M+K]^+$ is the predominant adduct signal of calotropin/calactin with an ion intensity of 3.7E2. For OTCD MALDI MSI, the derivatized calotropin/calactin signal is one of the most abundant signals with an ion intensity of 1.0E5.

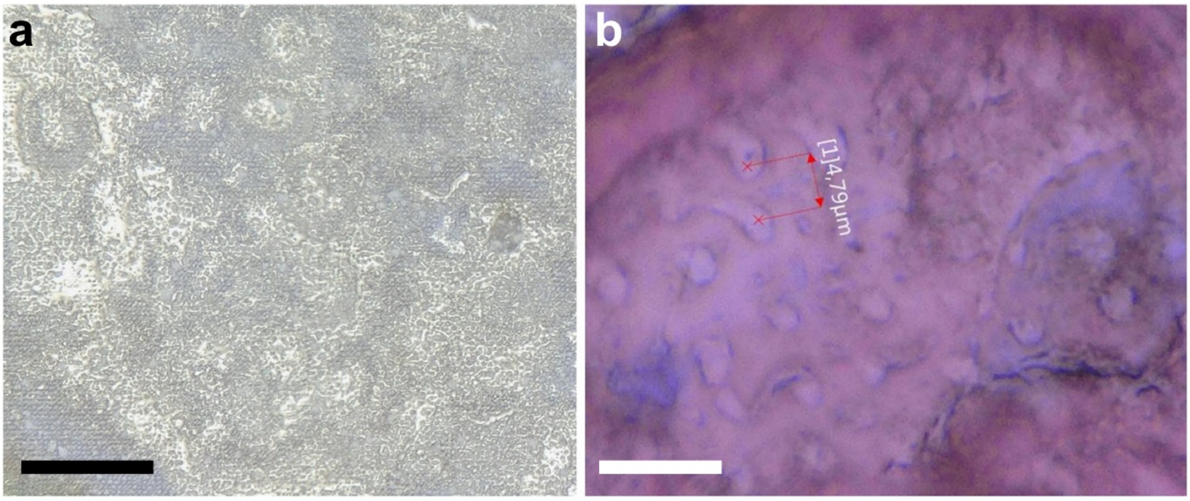

Figure S12. Optical images of Malpighian tubules after OTCD MALDI MSI performed with 5 µm step size using the AP-SMALDI[5] AF ion source. (**a**) Matrix-coated sample surface after measurement showing laser ablation craters of ≤ 5 µm. Thus, MSI analysis was performed without oversampling. (**b**) Magnification of a single H&E-stained Malpighian tubule after measurement, demonstrating 5 µm step size and laser ablation craters of ≤ 5 µm on the penetrated tissue. Scale bars, (a) 100 µm (b) 10 µm.

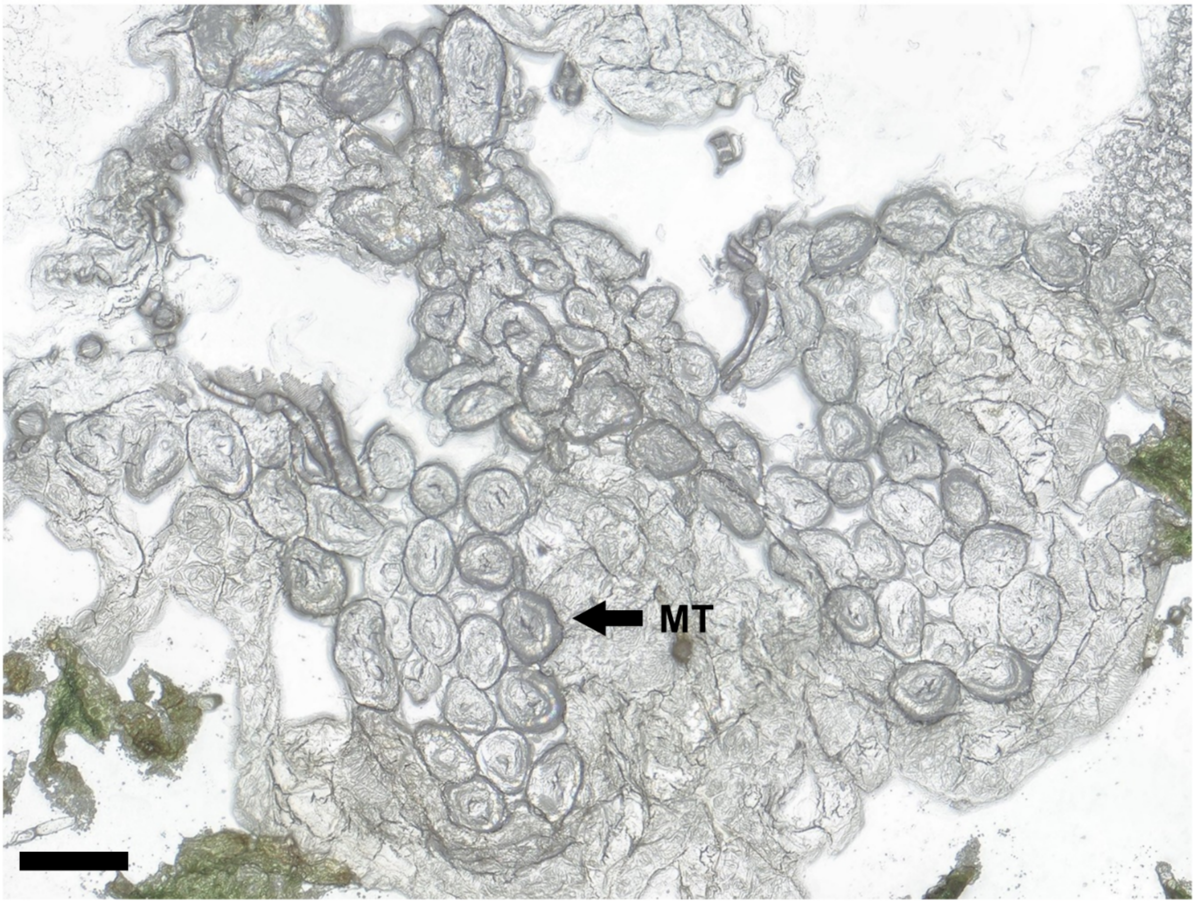

Figure S13. Optical image of longitudinal *D. plexippus* section (before OTCD and matrix application) highlighting transversal-sectioned Malpighian tubules (MT). Scale bar, 100 μm.

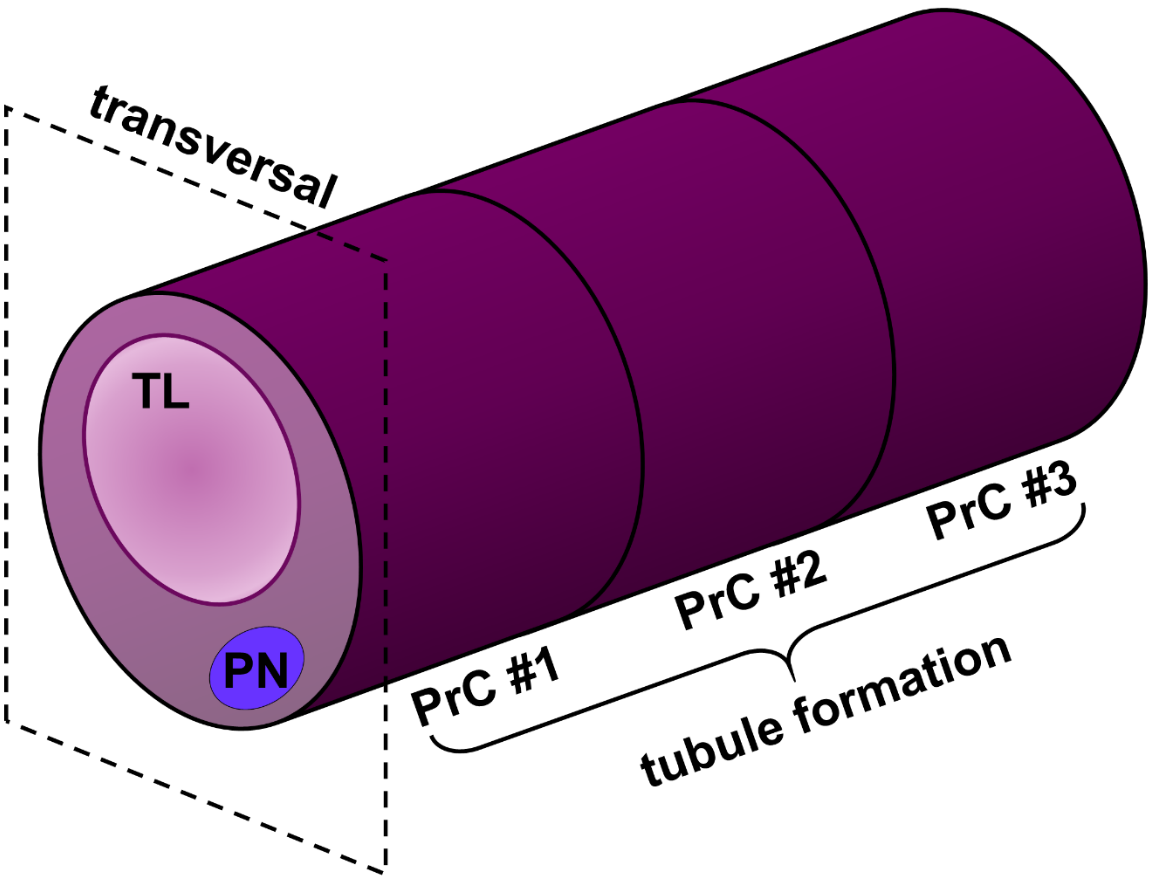

Figure S14. Simplified schematic showing the morphology of *D. plexippus* Malpighian tubules. The principal cells fold upon itself to form a tubule lumen. PrC: principal cell; PN: principal cell nucleus; TL: tubule lumen.

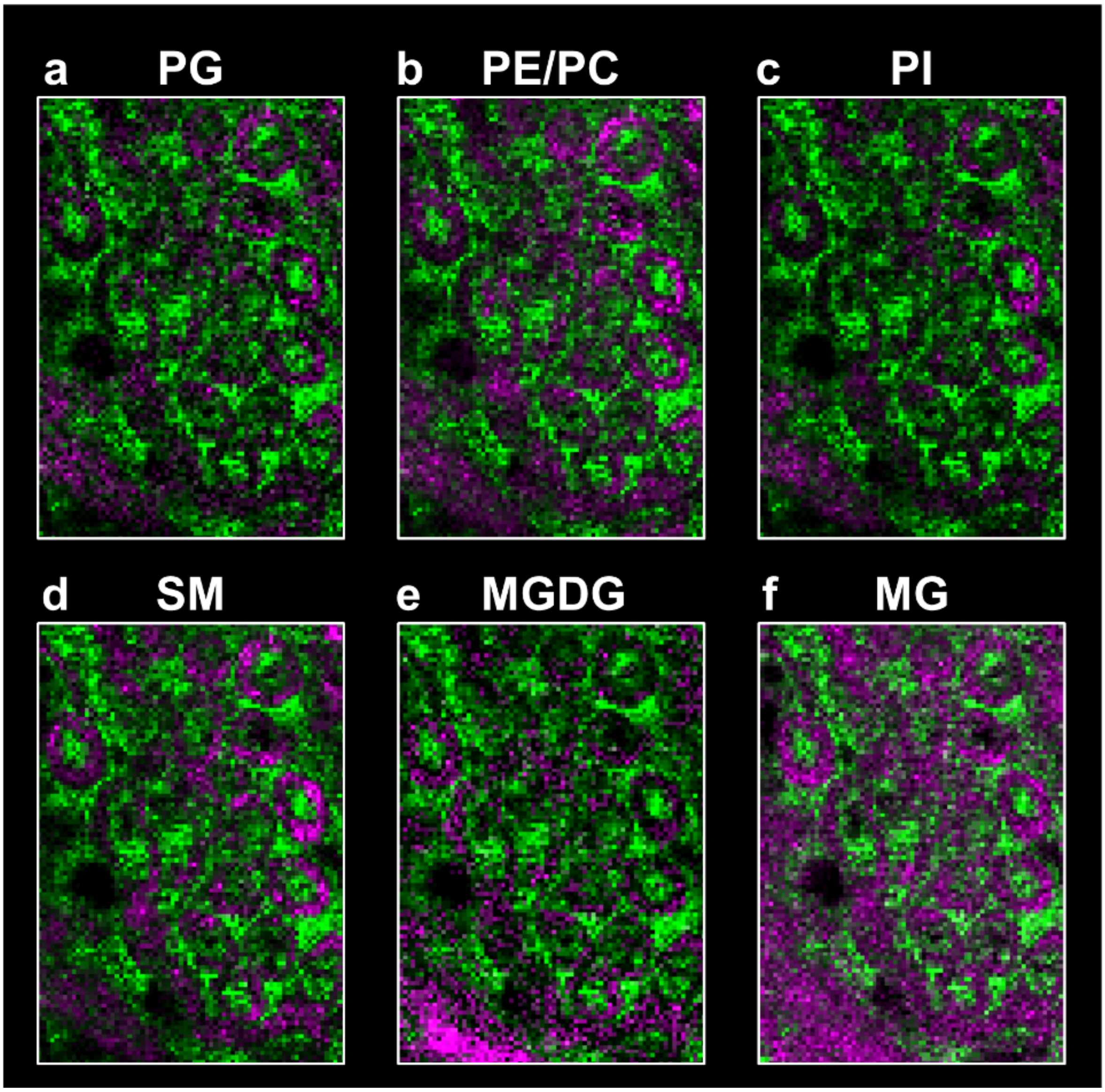

Figure S15. OTCD MALDI MSI of *D. plexippus* Malpighian tubules. Dual ion channel images obtained with 5 μm step size showing the spatial distribution for different lipid classes in pink. (**a**) $[PG(22:3/14:0)+Na]^+$ at *m/z* 795.5146, (**b**) $[PE(P\text{-}18:2/24:6)+Na]^+$ at *m/z* 822.5408 (**c**) $[PI(16:0/26:0)+H]^+$ at *m/z* 951.6896 (**d**) $[SM(16:0/34:6)+Na]^+$ at *m/z* 955.7238 (**e**) $[MGDG(16:2/20:3)+H]^+$ at *m/z* 777.5511 (**f**) $[MG(19:0)+Na]^+$ at *m/z* 395.3132. The nucleotide derivative thymidine 3',5'-hydrogen phosphate ($[M+K]^+$, *m/z* 343.0092) is shown in green.

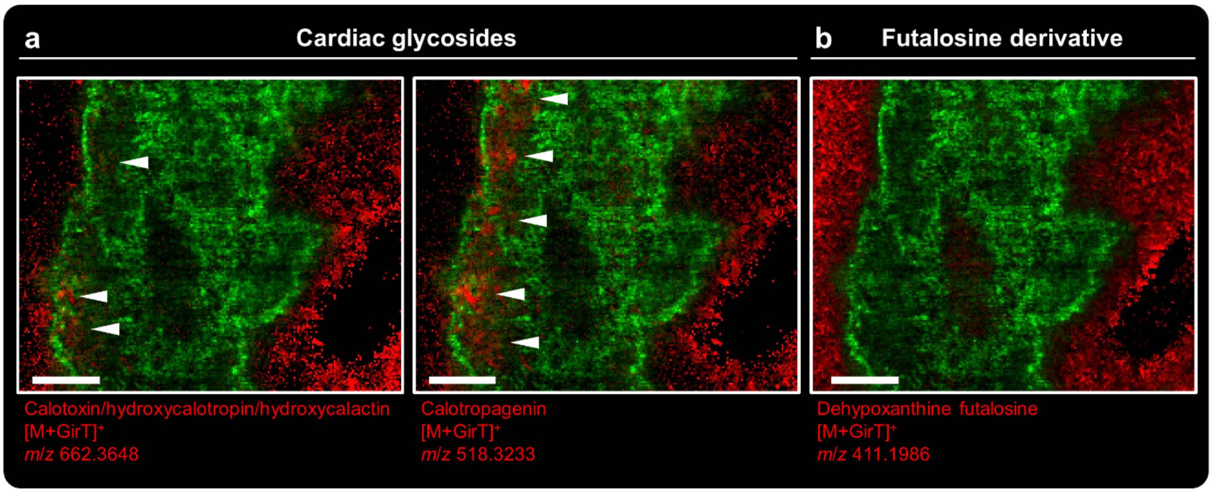

Figure S16. **OTCD MALDI MSI (2 µm step size) of derivatized cardiac glycosides and futalosine derivative uptake in *D. plexippus*.** Red-green overlay images showing the spatial distribution for (**a**) the derivatized isomers calotoxin/hydroxycalotropin/hydroxycalactin ([M+GirT]$^+$, *m*/*z* 662.3648) and calotropagenin ([M+GirT]$^+$, *m*/*z* 518.3233) in red, (**b**) dehypoxanthine futalosine ([M+GirT]+, m/z 411.1986) in red and (**a**,**b**) PE(46:3) ([M+H]$^+$, *m*/*z* 882.6922) in green. In contrast to both cardiac glycosides, the derivatized futalosine species is not accumulated in the outer layer of the fat body tissue of the caterpillar. Scale bar, 80 µm.

Supplementary Data 1: Structure and LC-MS^2 spectra for all detected cardiac glycosides. Red arrows indicate water loss and green arrows indicate carbon monoxide loss.

uzarigenin

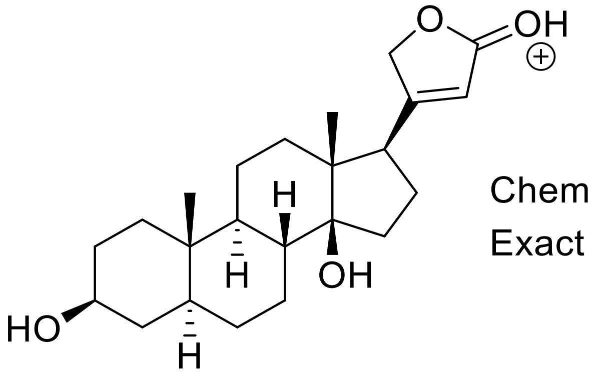

Chemical Formula: $C_{23}H_{35}O_4^+$

Exact Mass: 375.25

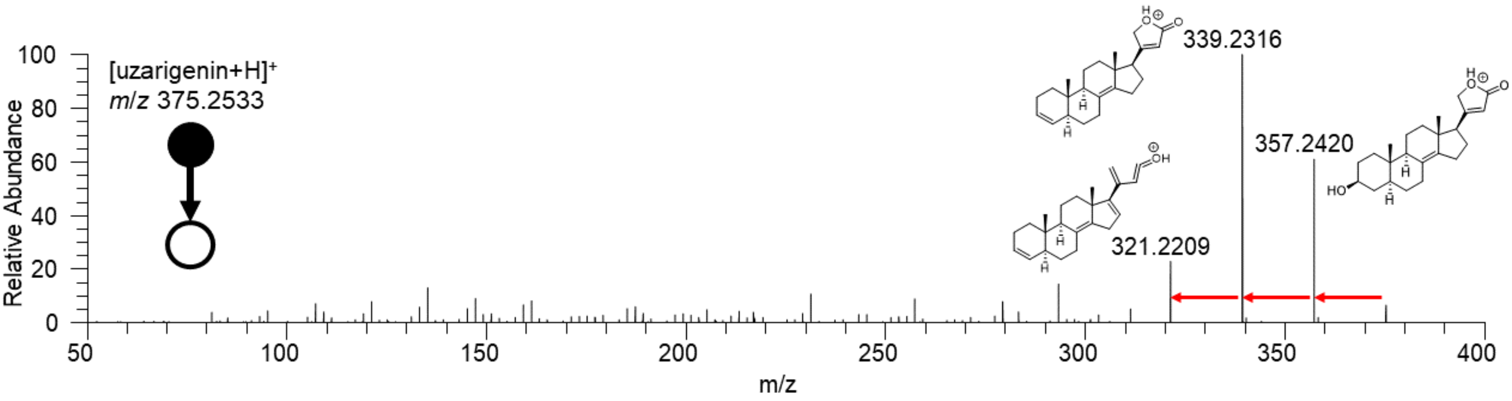

calotropagenin

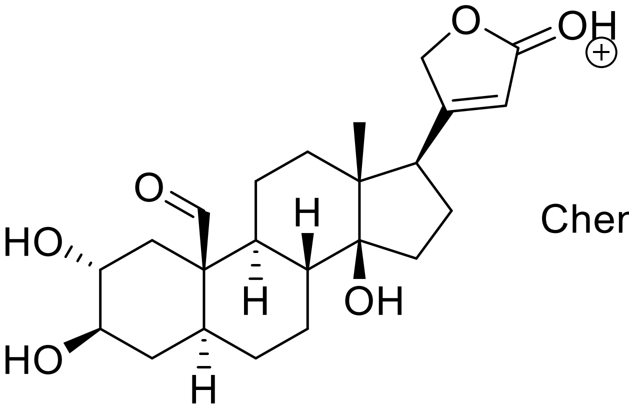

Chemical Formula: $C_{23}H_{33}O_6^+$
Exact Mass: 405.23

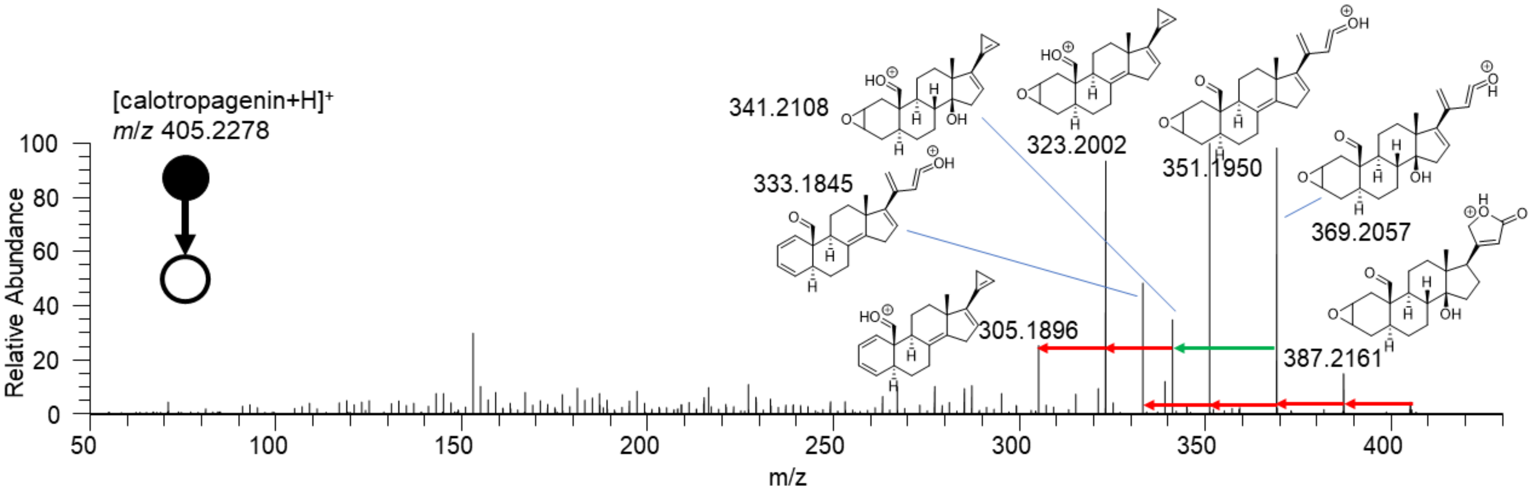

strophanthidol

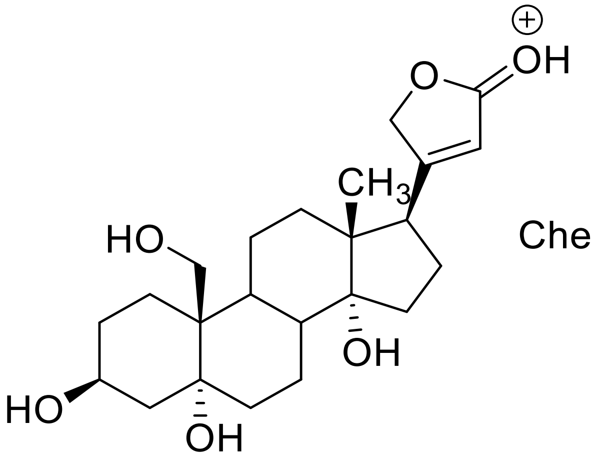

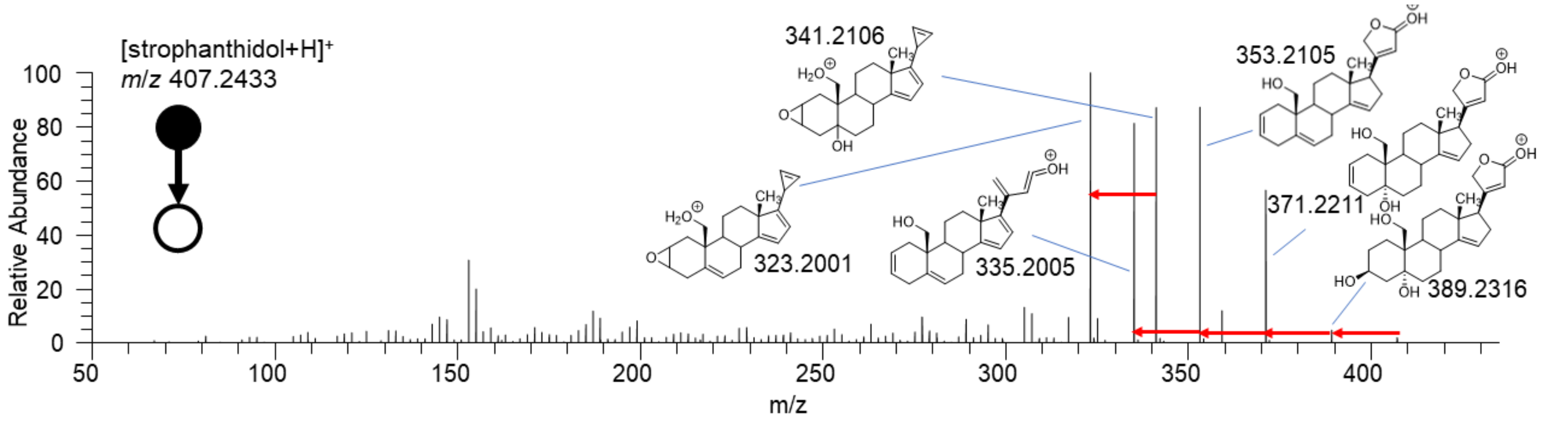

digitoxigenin digitoxoside

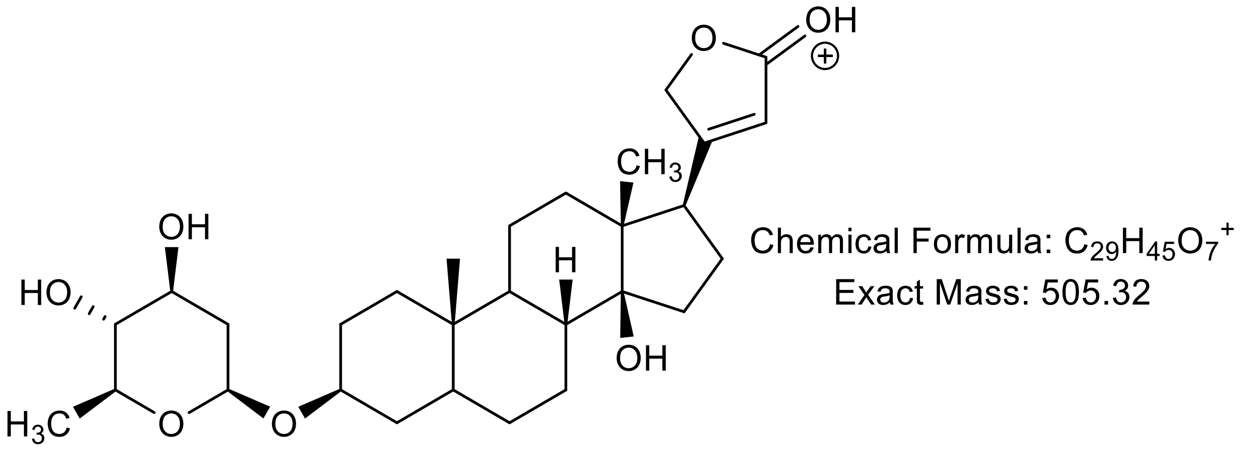

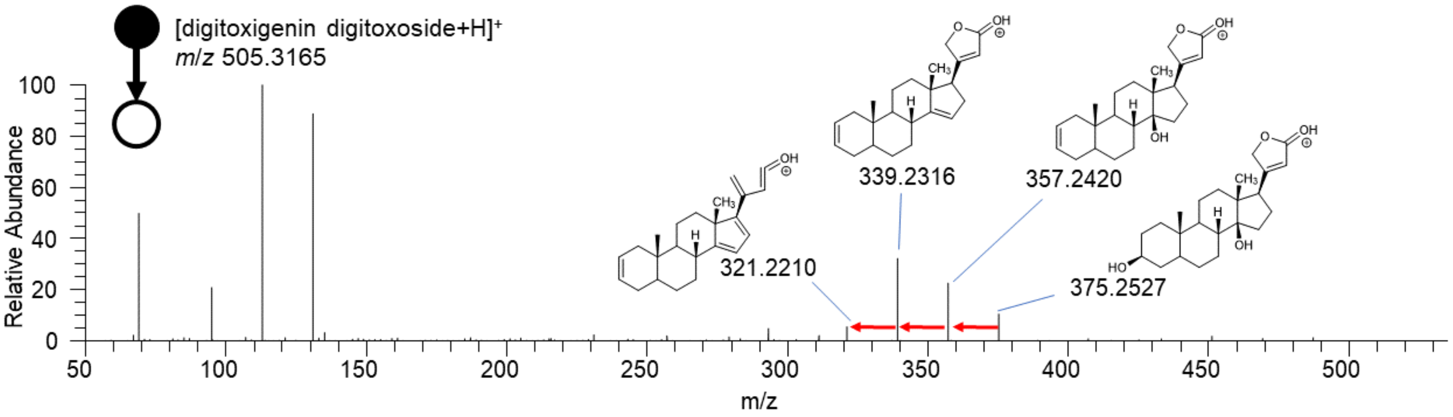

strobosid

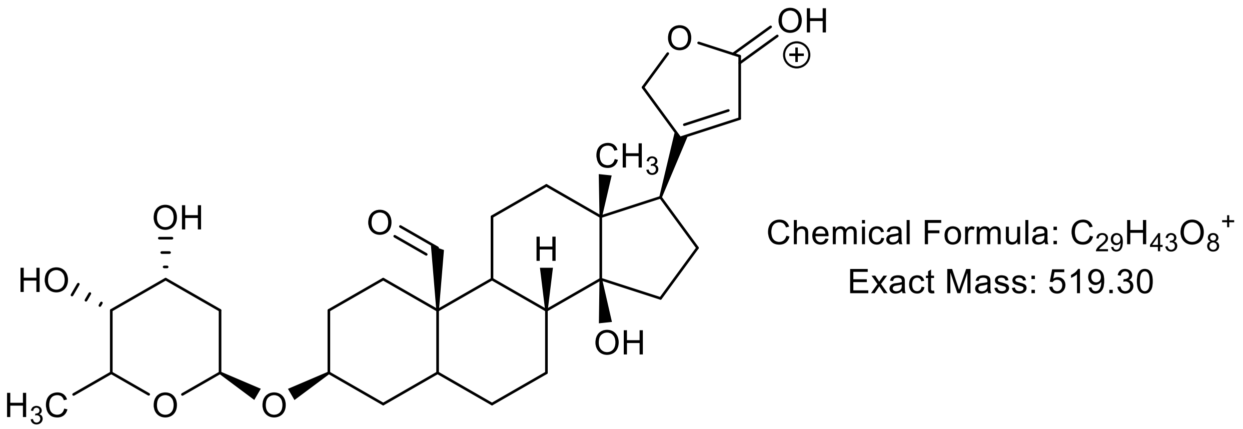

OH
O
CH3
OH
O
HO
H
OH
H3C
O
O
Chemical Formula: C29H43O8+
Exact Mass: 519.30

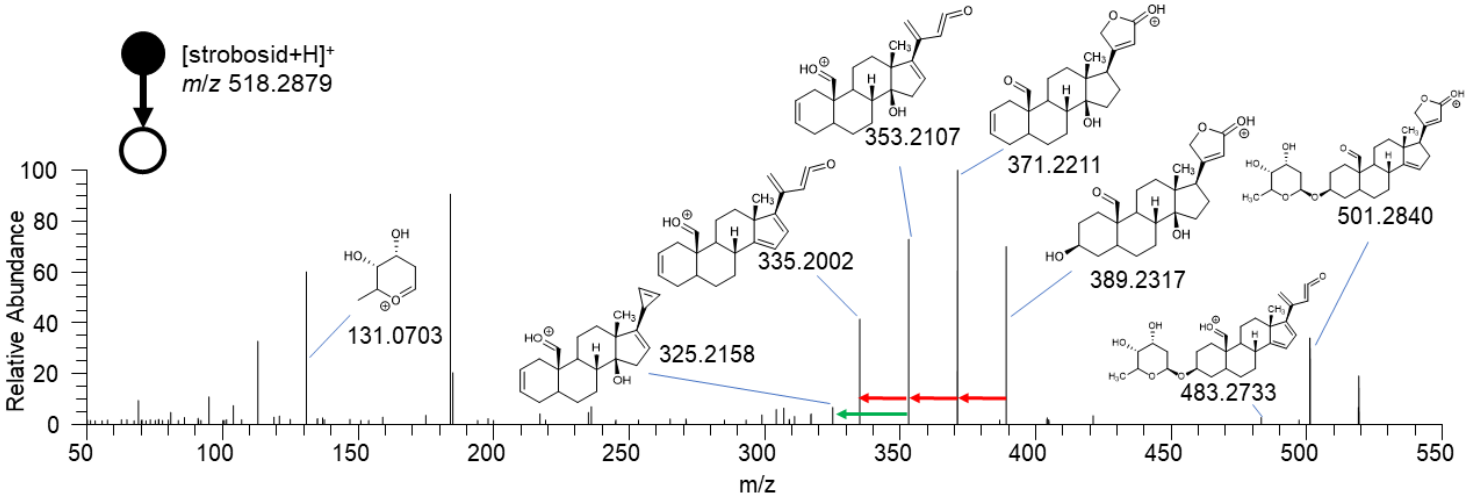

[strobosid+H]+
m/z 518.2879
Relative Abundance
m/z
131.0703
325.2158
335.2002
353.2107
371.2211
389.2317
483.2733
501.2840

uscharidin

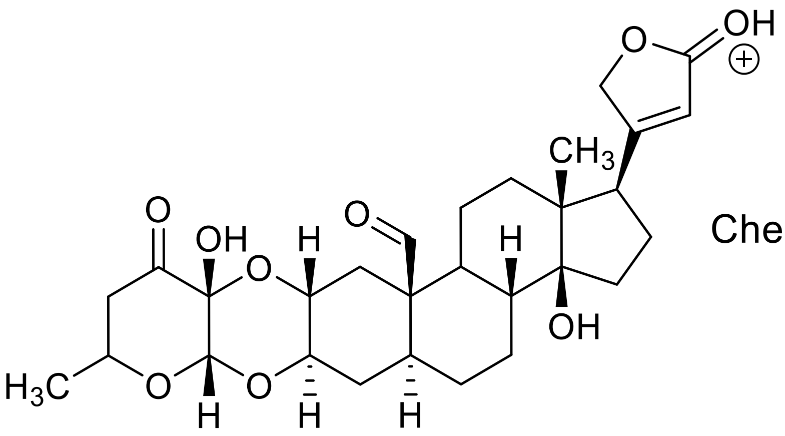

Chemical Formula: $C_{29}H_{39}O_{9}^{+}$

Exact Mass: 531.26

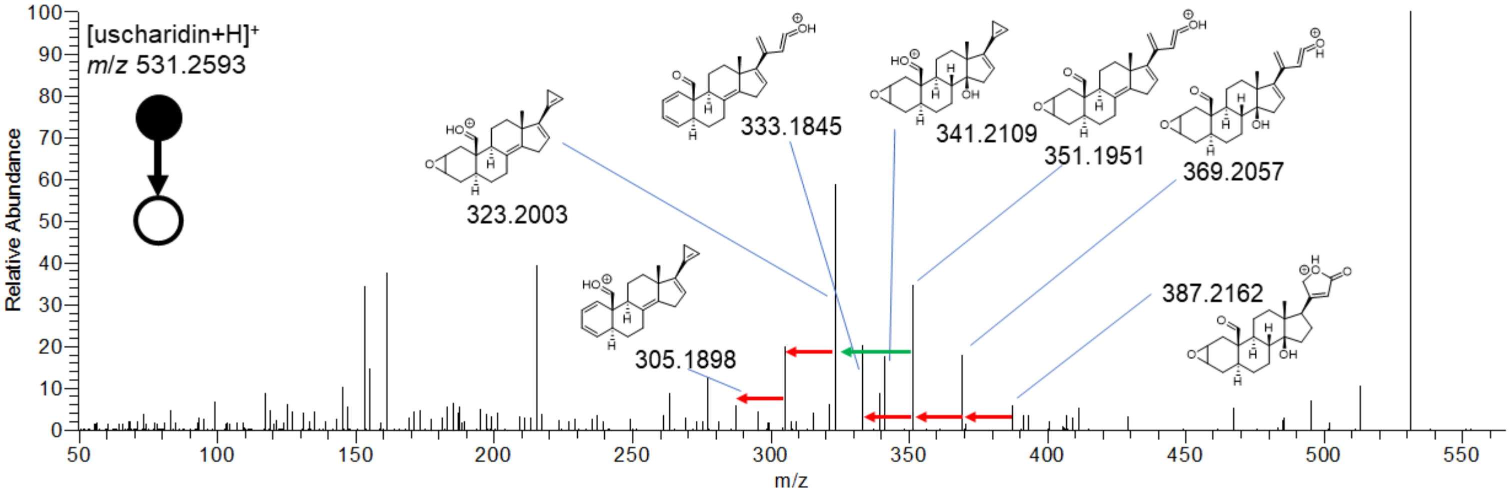

calotropin

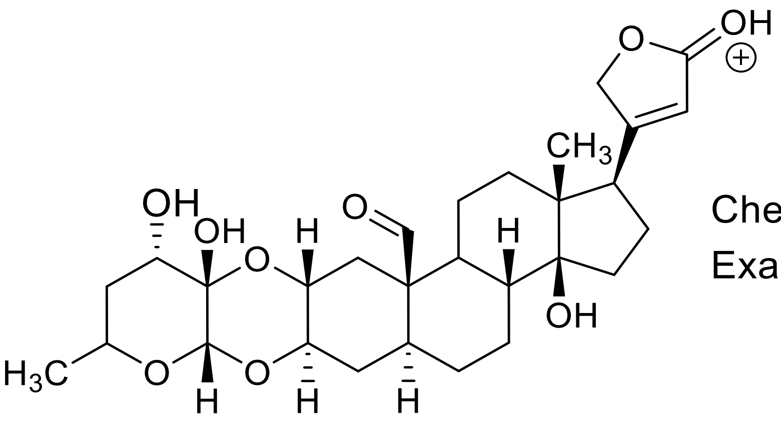

Chemical Formula: $C_{29}H_{41}O_9^+$
Exact Mass: 533.27

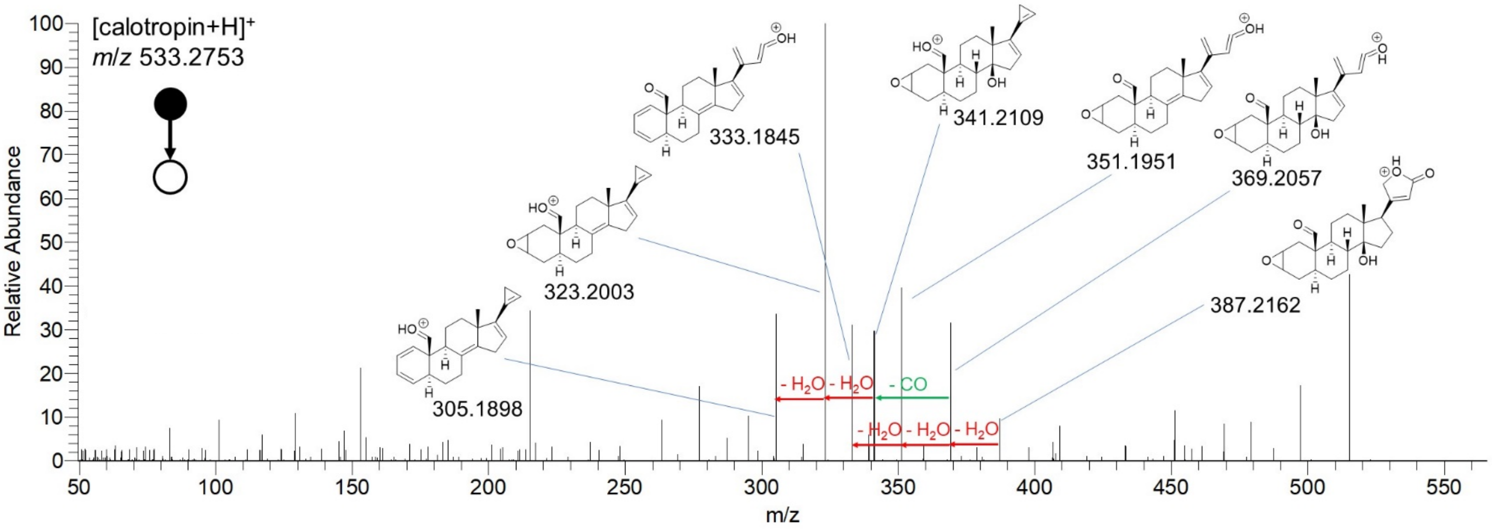

calactin

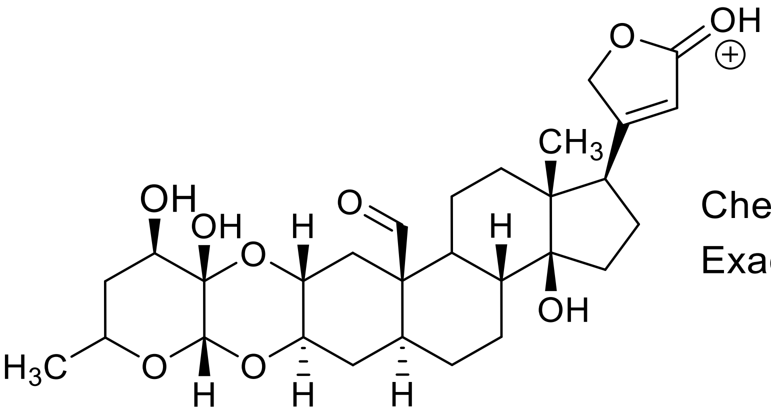
Chemical Formula: $C_{29}H_{41}O_9^+$
Exact Mass: 533.27

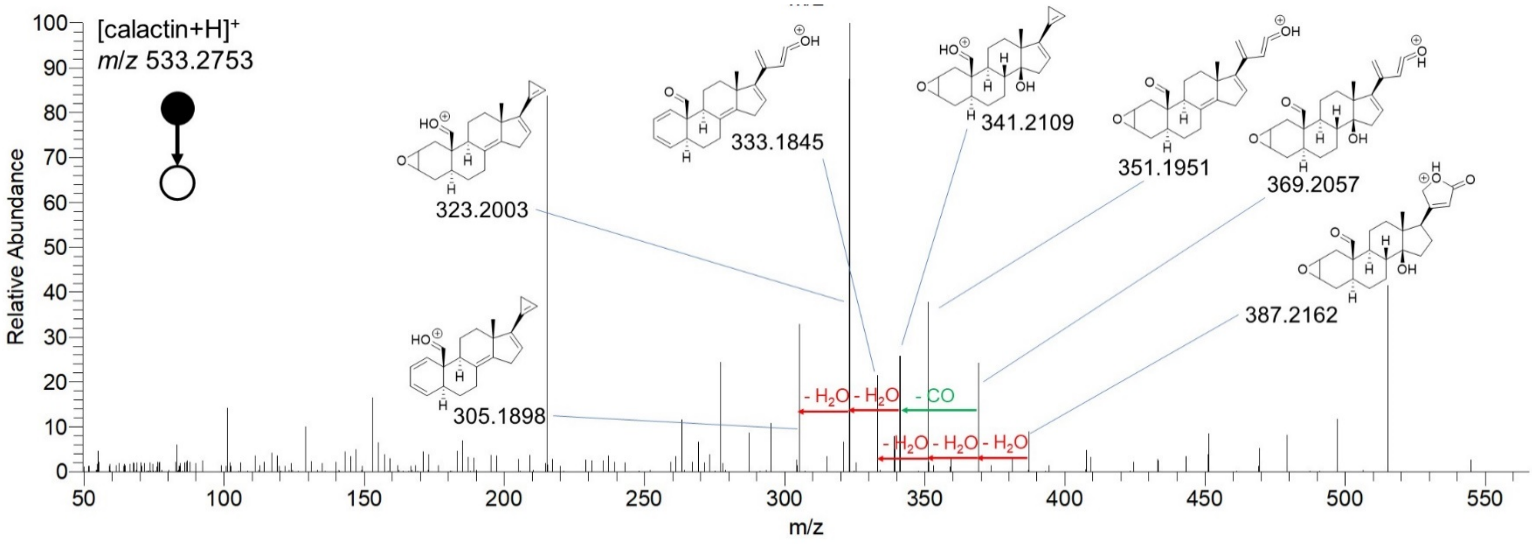
[calactin+H]+
m/z 533.2753
Relative Abundance
m/z
323.2003
333.1845
341.2109
351.1951
369.2057
387.2162
305.1898
- H2O
- CO

gofruside

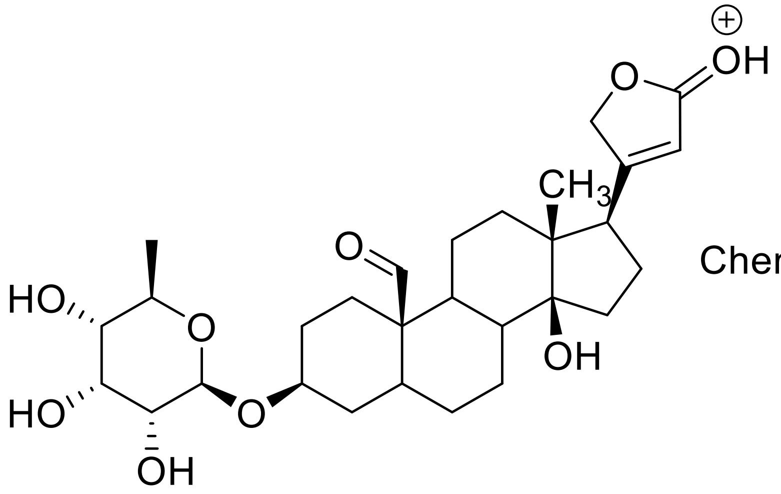

Chemical Formula: $C_{29}H_{43}O_9^+$
Exact Mass: 535.29

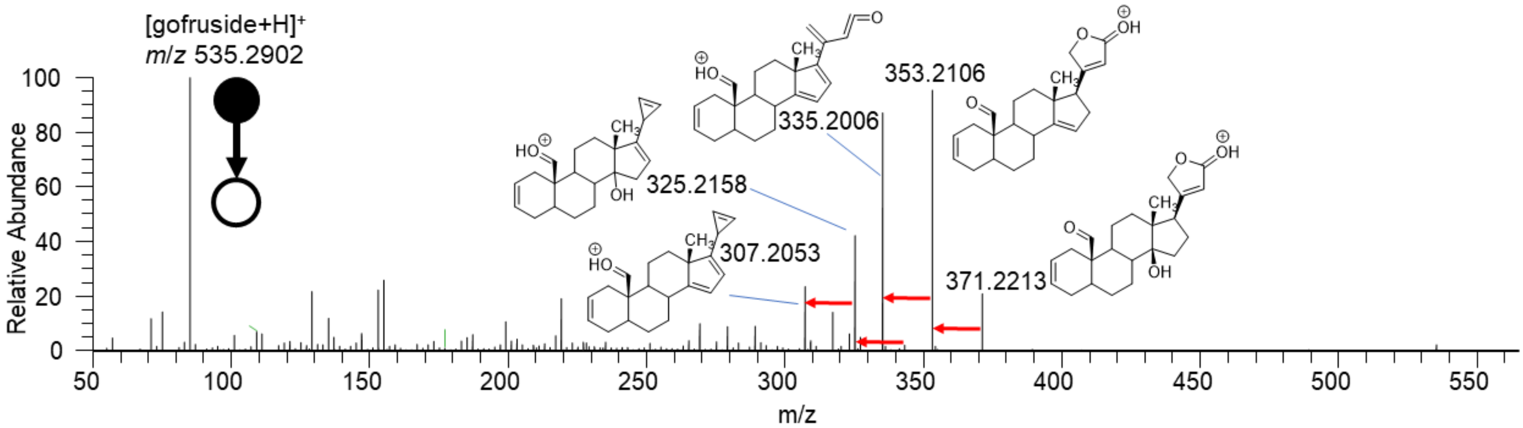

frugoside

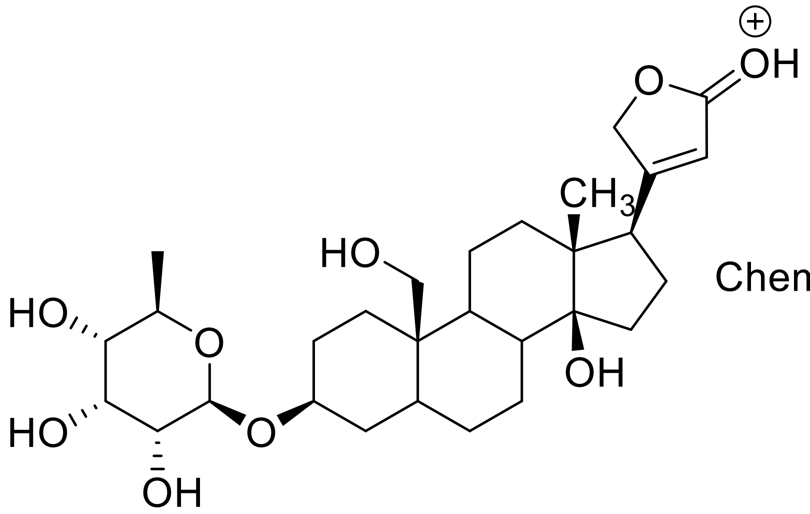

Chemical Formula: $C_{29}H_{45}O_9^+$
Exact Mass: 537.31

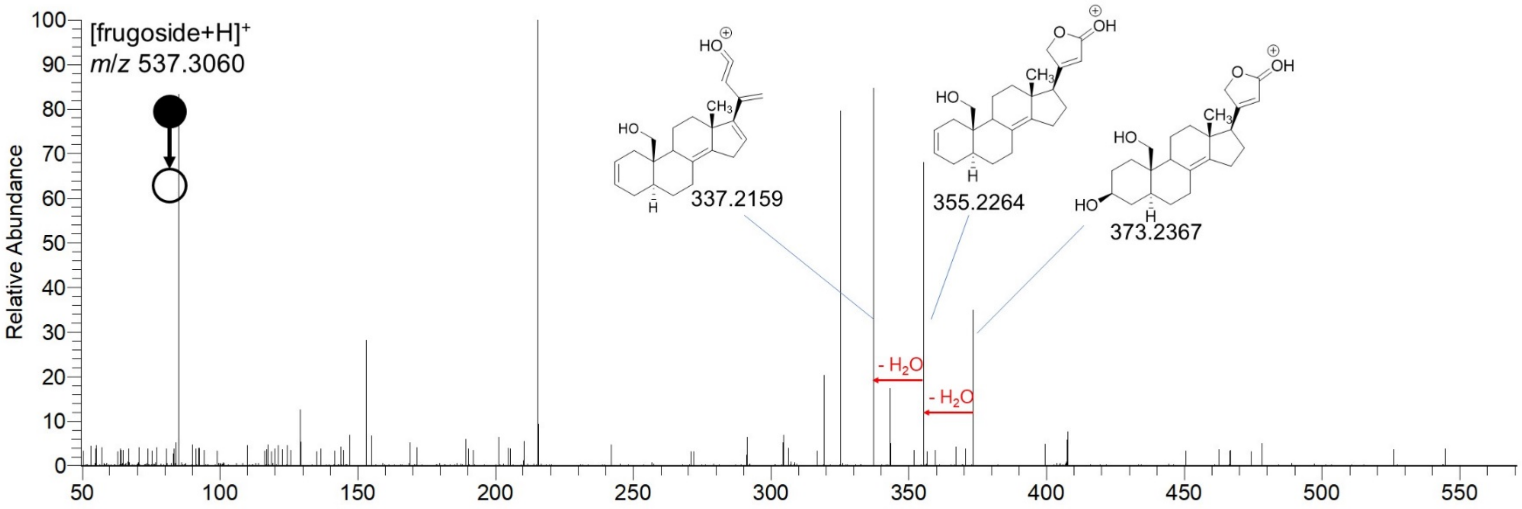

hydroxyuscharidin

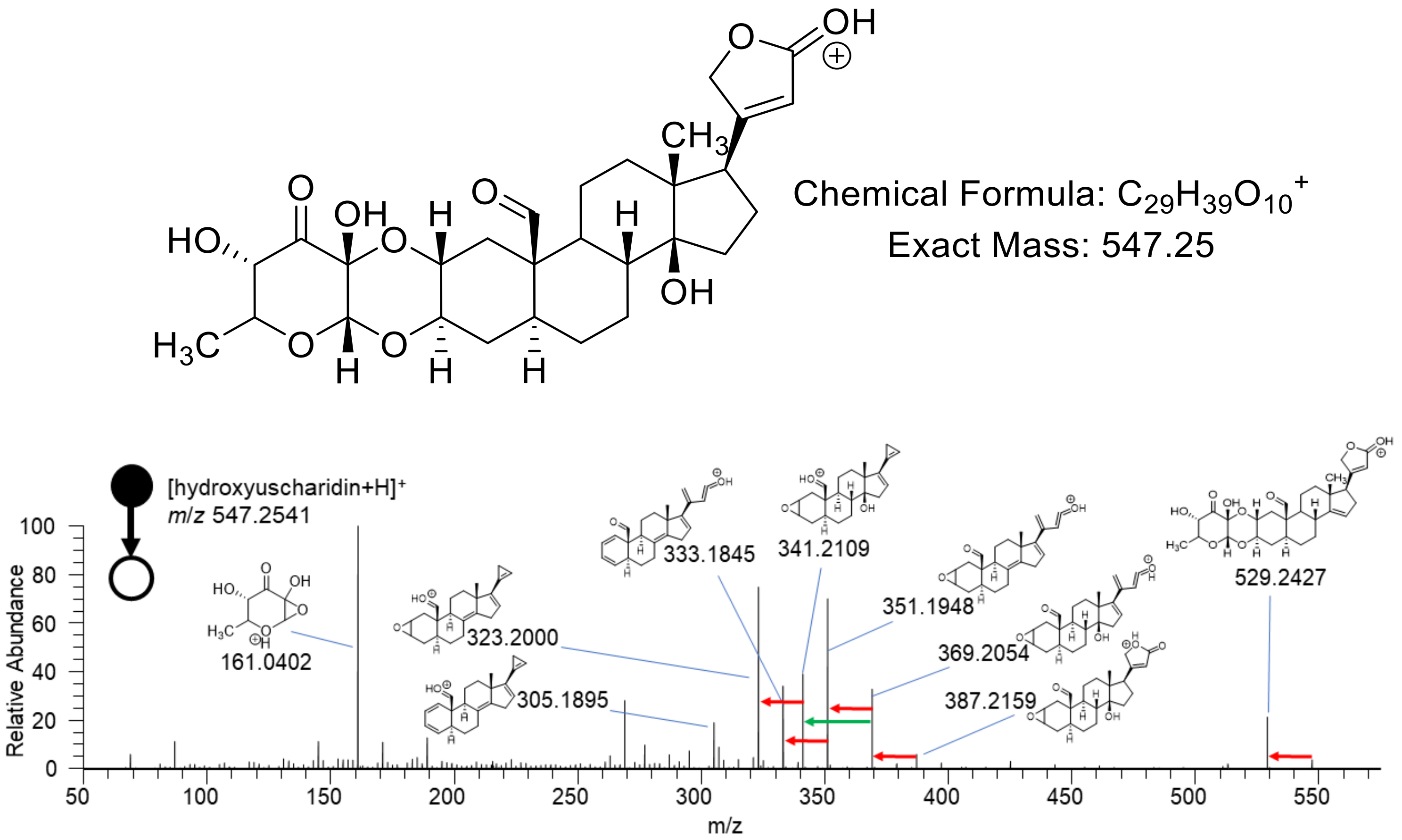

calotoxin

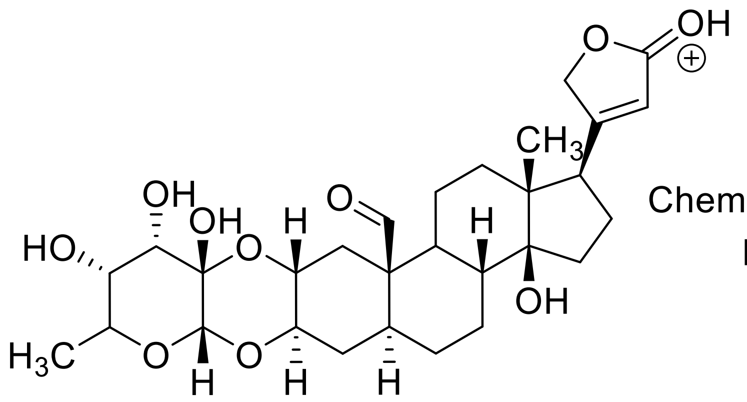
Chemical Formula: $C_{29}H_{41}O_{10}^{+}$
Exact Mass: 549.27

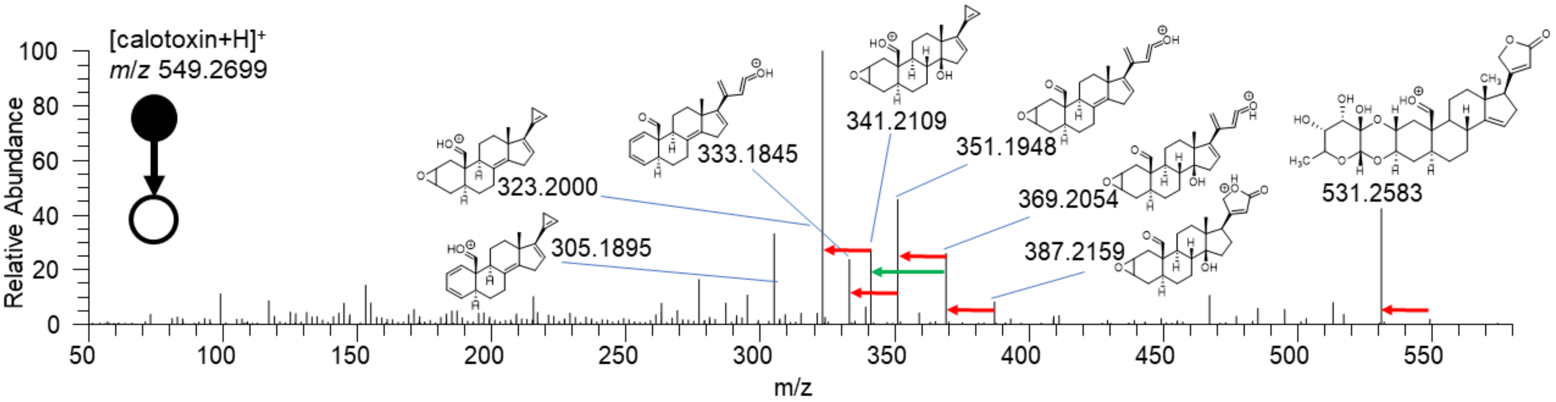
[calotoxin+H]+
m/z 549.2699
Relative Abundance
m/z
305.1895
323.2000
333.1845
341.2109
351.1948
369.2054
387.2159
531.2583

hydroxycalotropin

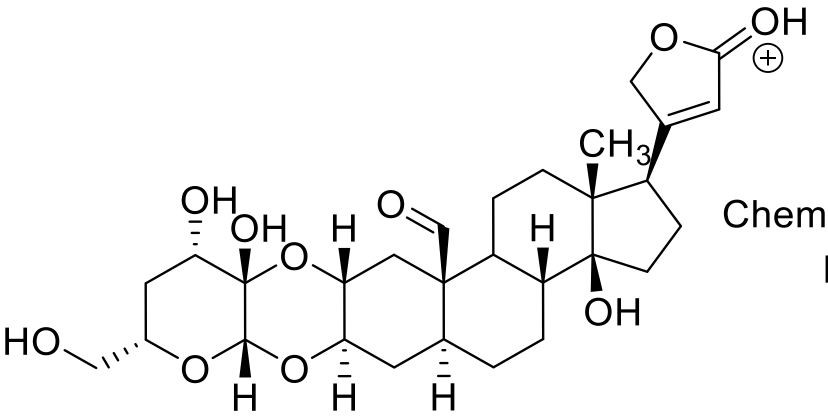

Chemical Formula: $C_{29}H_{41}O_{10}^{+}$

Exact Mass: 549.27

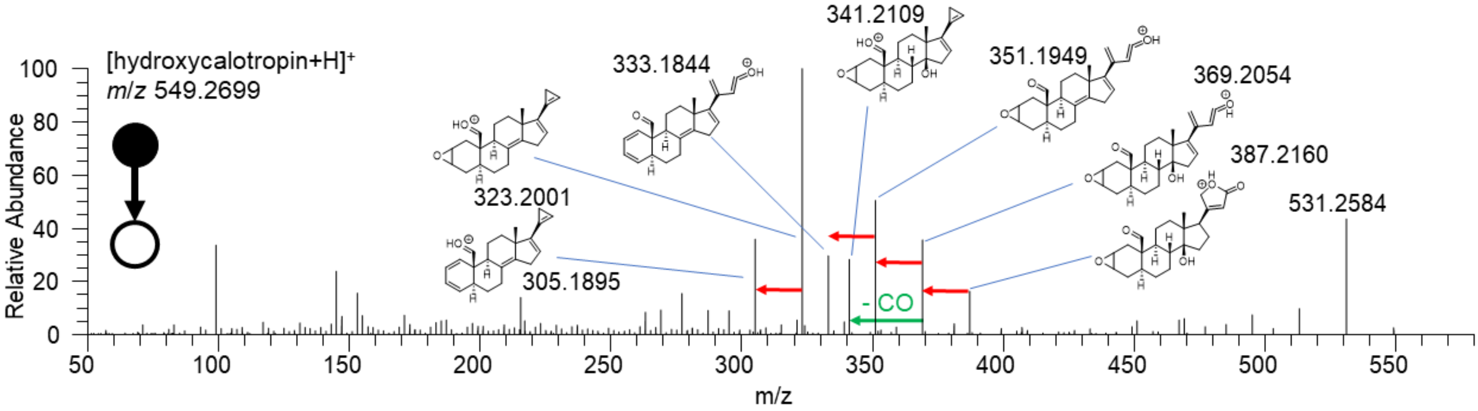

hydroxycalactin

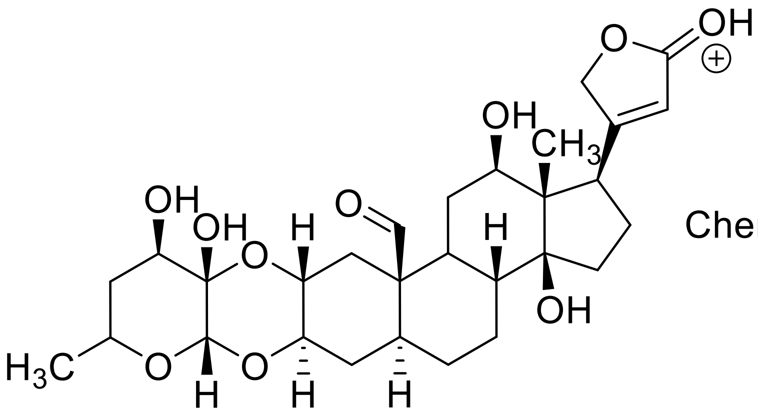

Chemical Formula: $C_{29}H_{41}O_{10}^{+}$

Exact Mass: 549.27

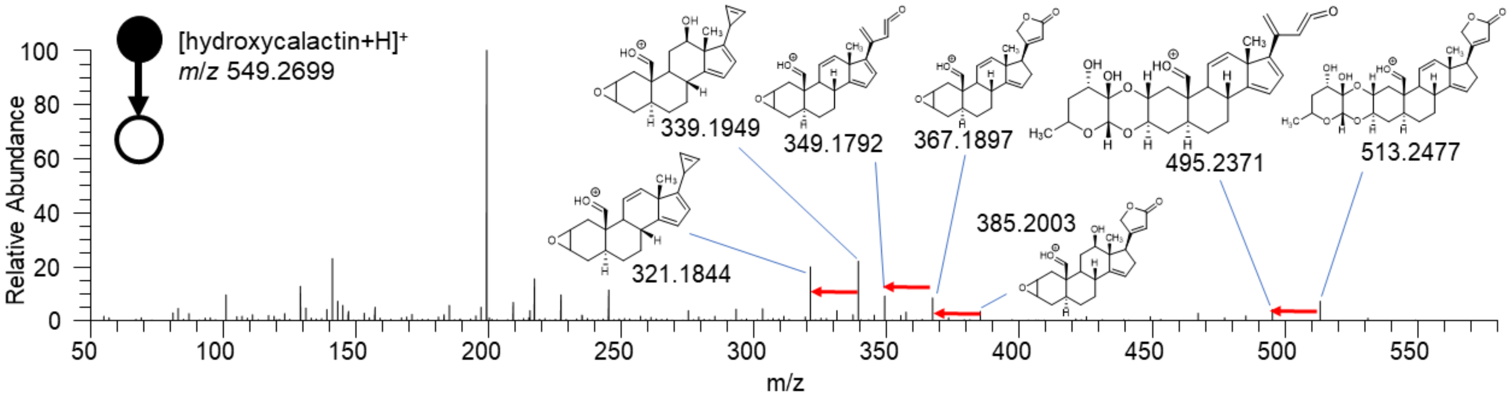

calactinic acid

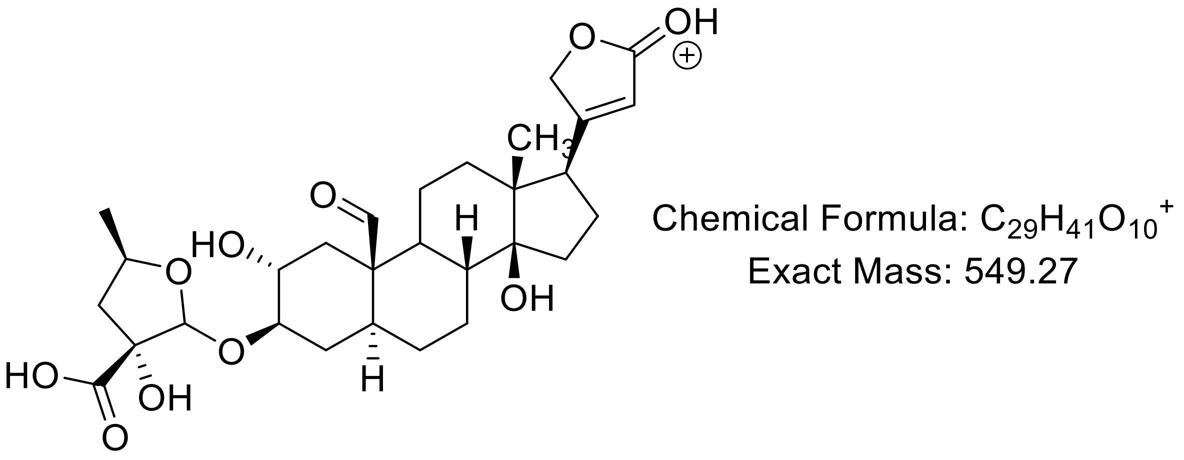

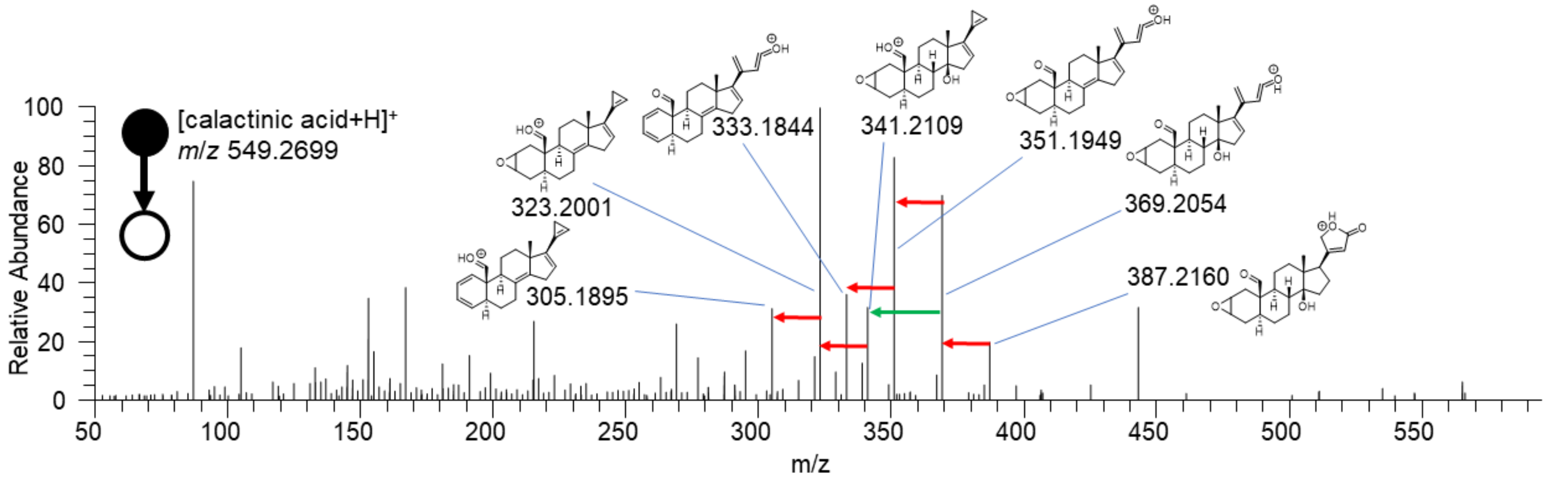

corglycon

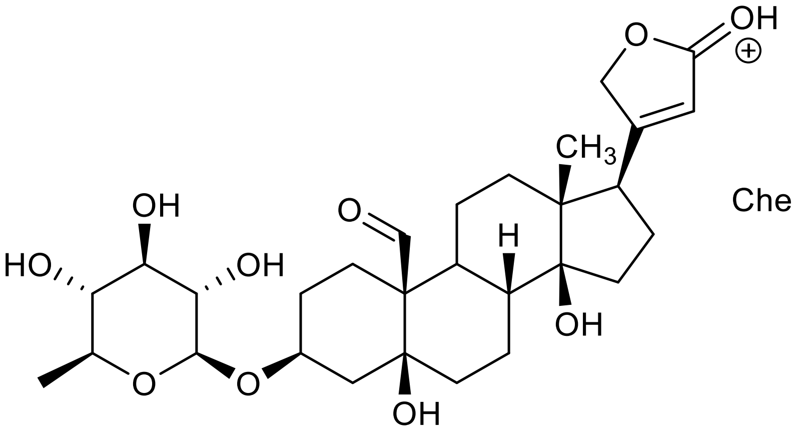

Chemical Formula: $C_{29}H_{43}O_{10}^{+}$
Exact Mass: 551.29

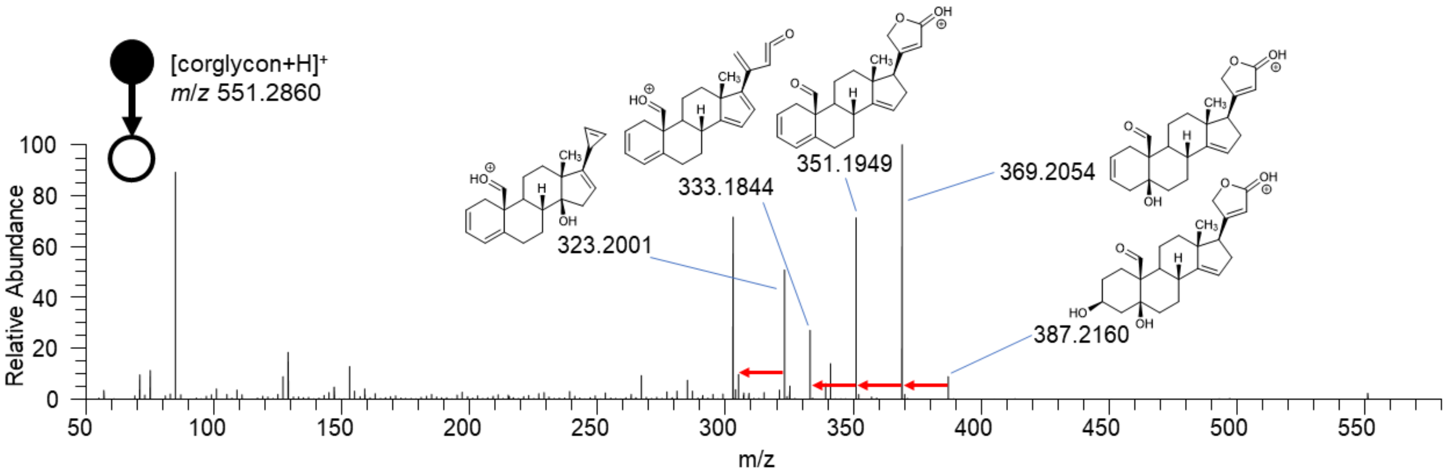

[corglycon+H]+
m/z 551.2860
Relative Abundance
m/z
323.2001
333.1844
351.1949
369.2054
387.2160

periplogenin-glycoside

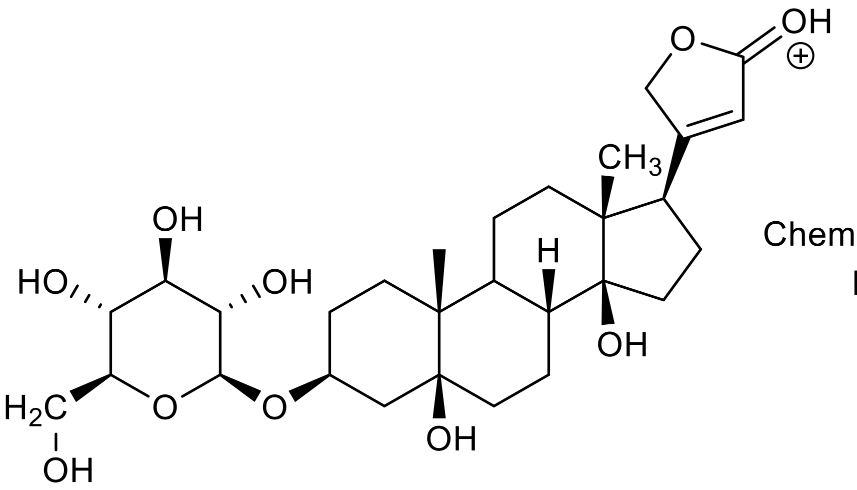

Chemical Formula: $C_{29}H_{45}O_{10}^{+}$
Exact Mass: 553.30

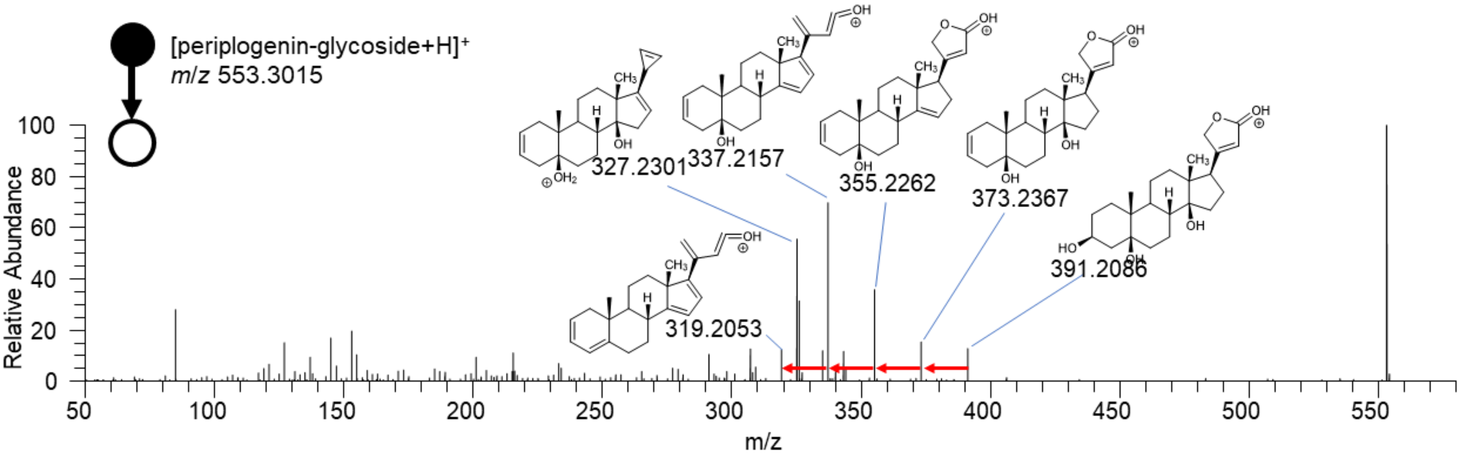

gomphoside-acetate

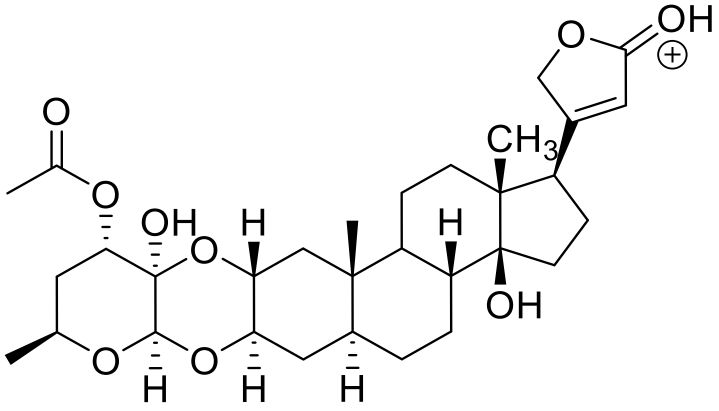

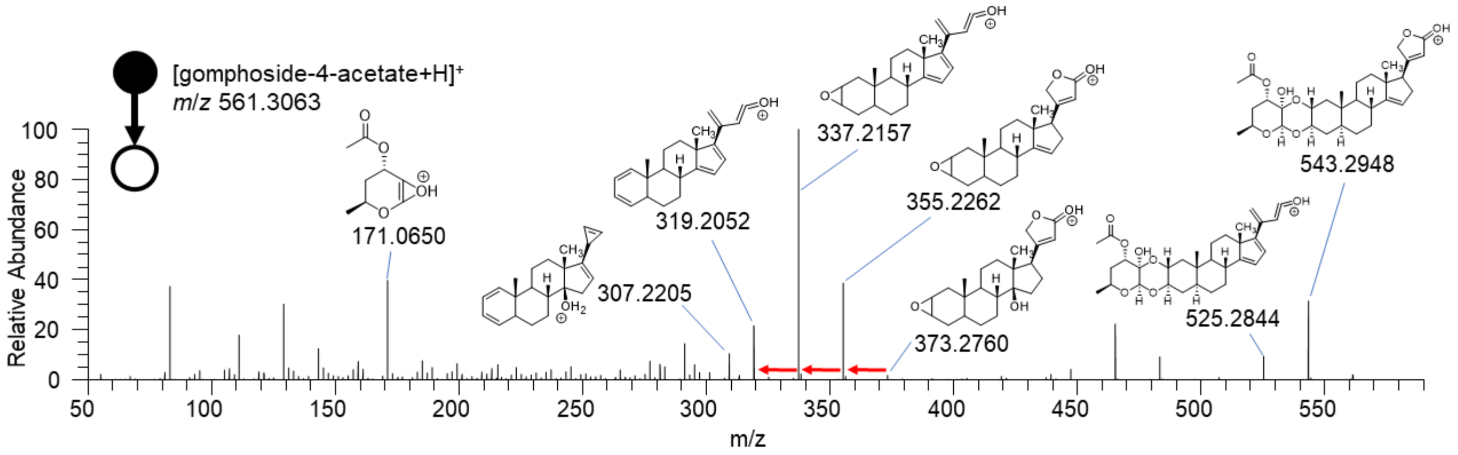

asclepin

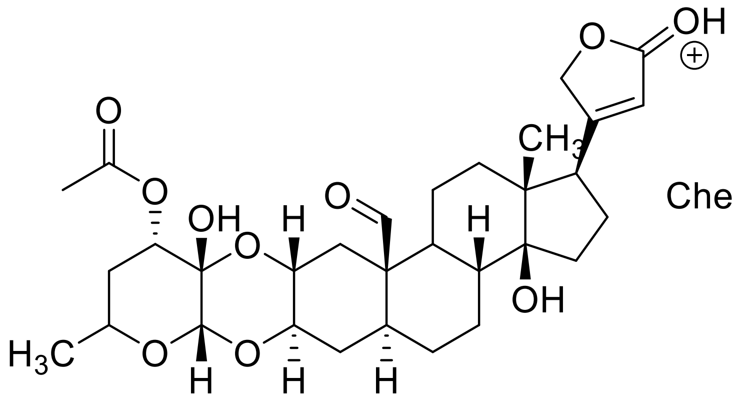

Chemical Formula: $C_{31}H_{43}O_{10}^{+}$
Exact Mass: 575.29

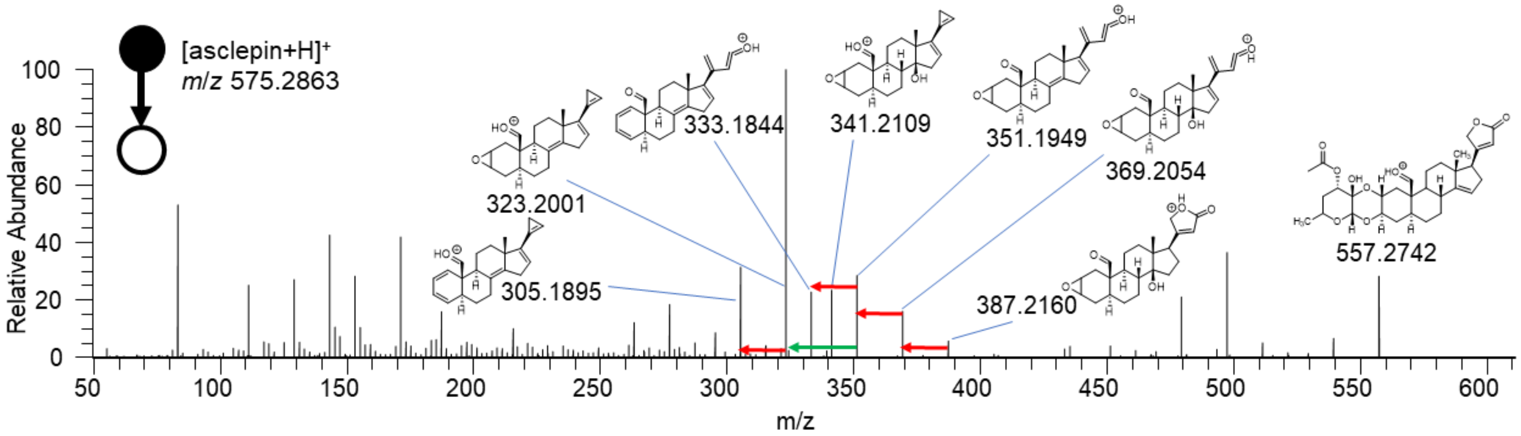

[asclepin+H]+
m/z 575.2863
Relative Abundance
m/z
323.2001
333.1844
341.2109
351.1949
369.2054
305.1895
387.2160
557.2742

hydroxycalactinic acid methylester

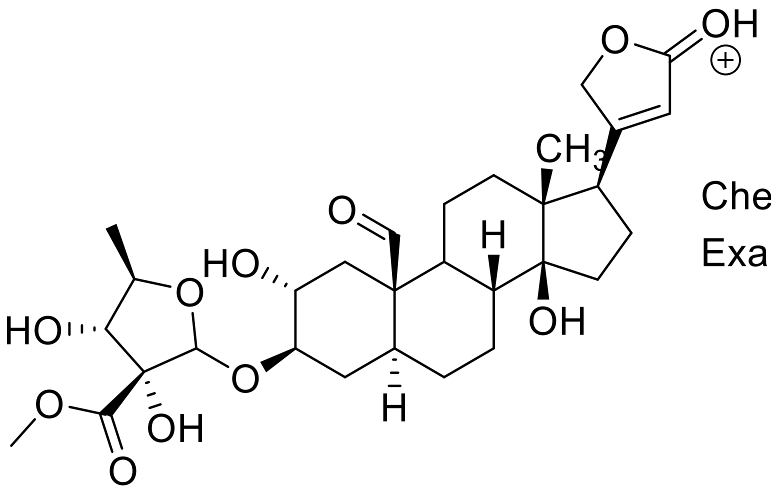

Chemical Formula: $C_{30}H_{43}O_{11}^{+}$

Exact Mass: 579.28

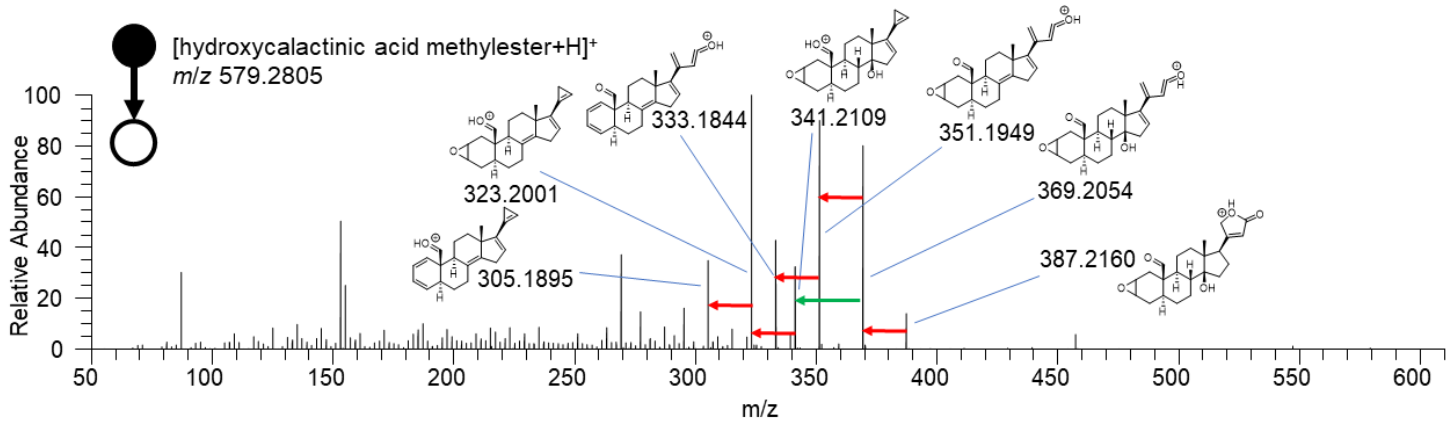

uscharin

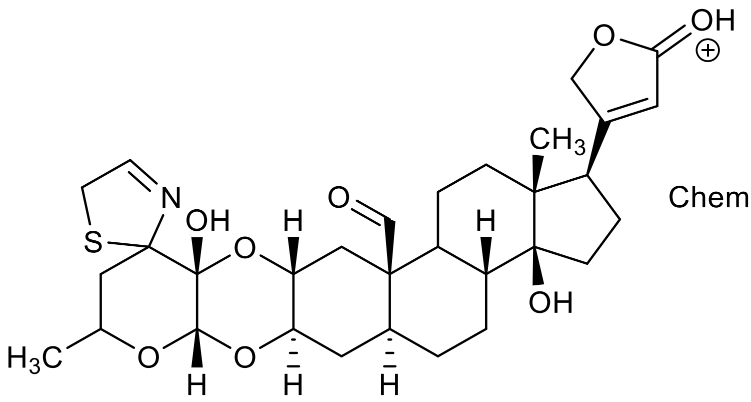
Chemical Formula: $C_{31}H_{42}NO_8S^+$
Exact Mass: 588.26

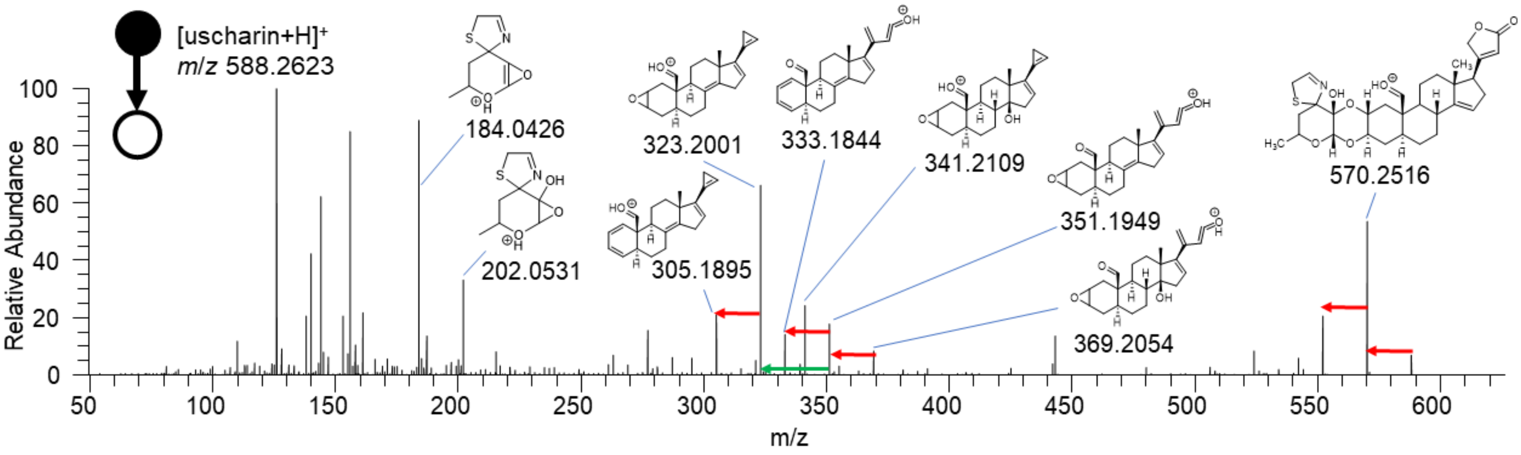
[uscharin+H]+
m/z 588.2623
184.0426
202.0531
305.1895
323.2001
333.1844
341.2109
351.1949
369.2054
570.2516
Relative Abundance
m/z

voruscharin

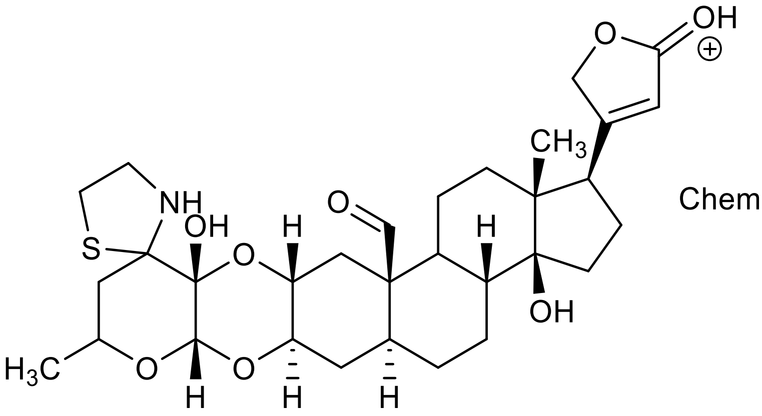

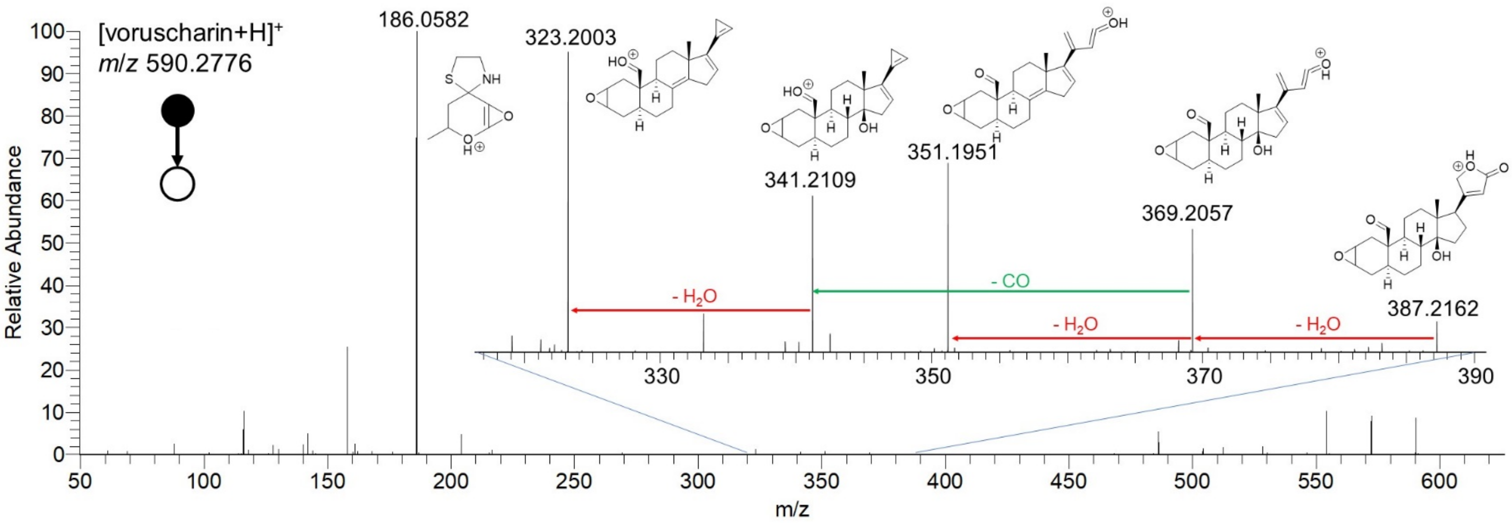

acetoxycalotropin

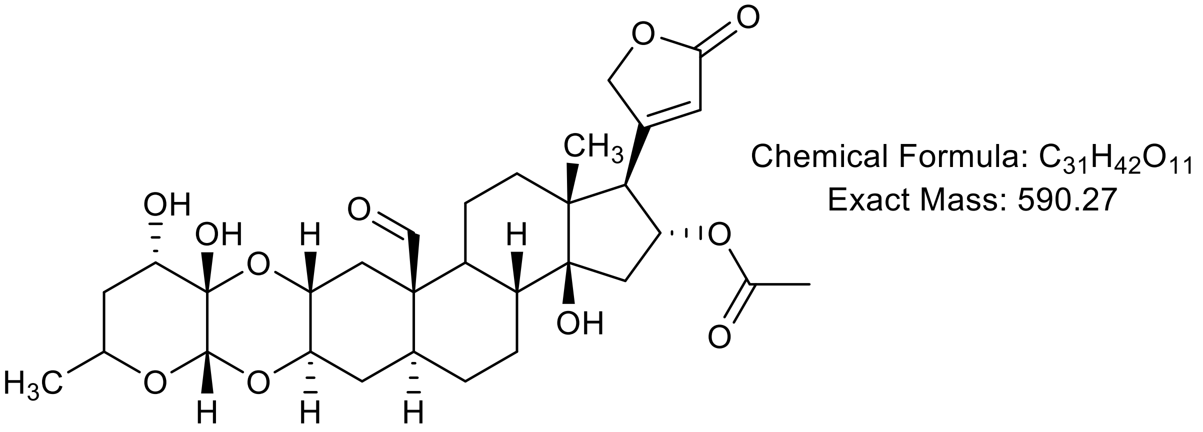

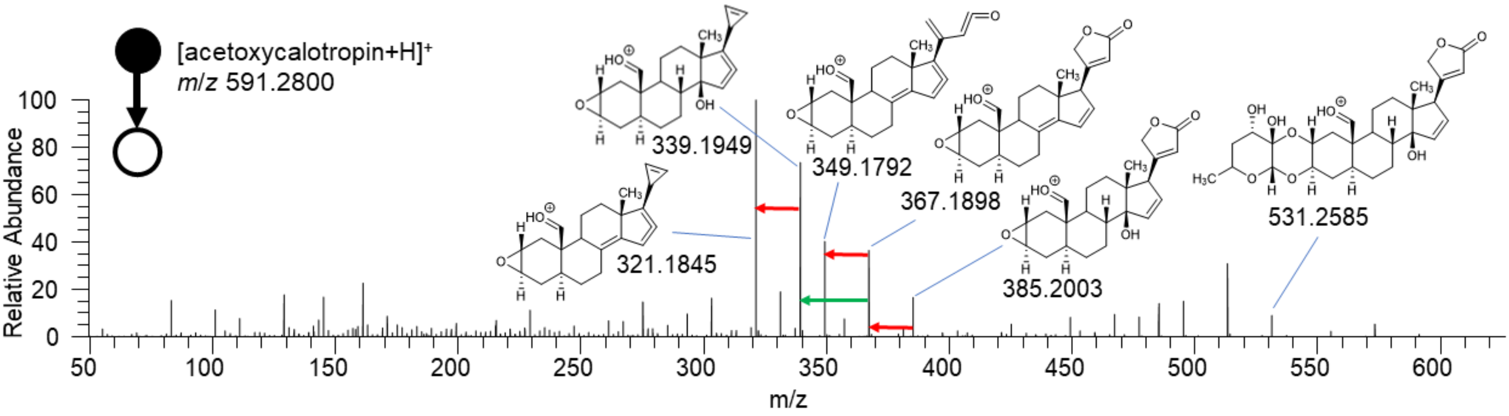

hydroxyasclepin

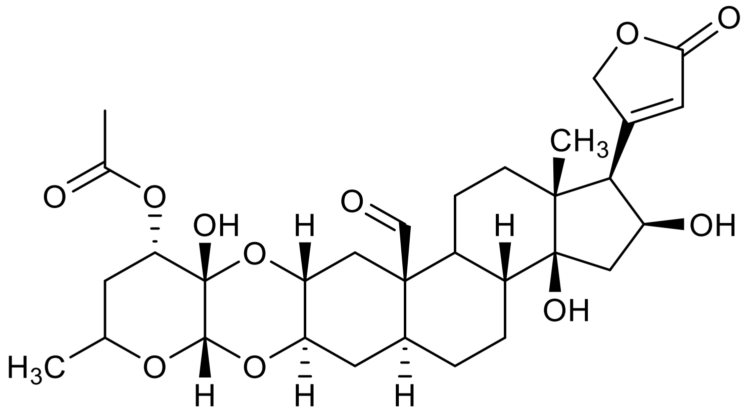

Chemical Formula: $C_{31}H_{42}O_{11}$
Exact Mass: 590.27

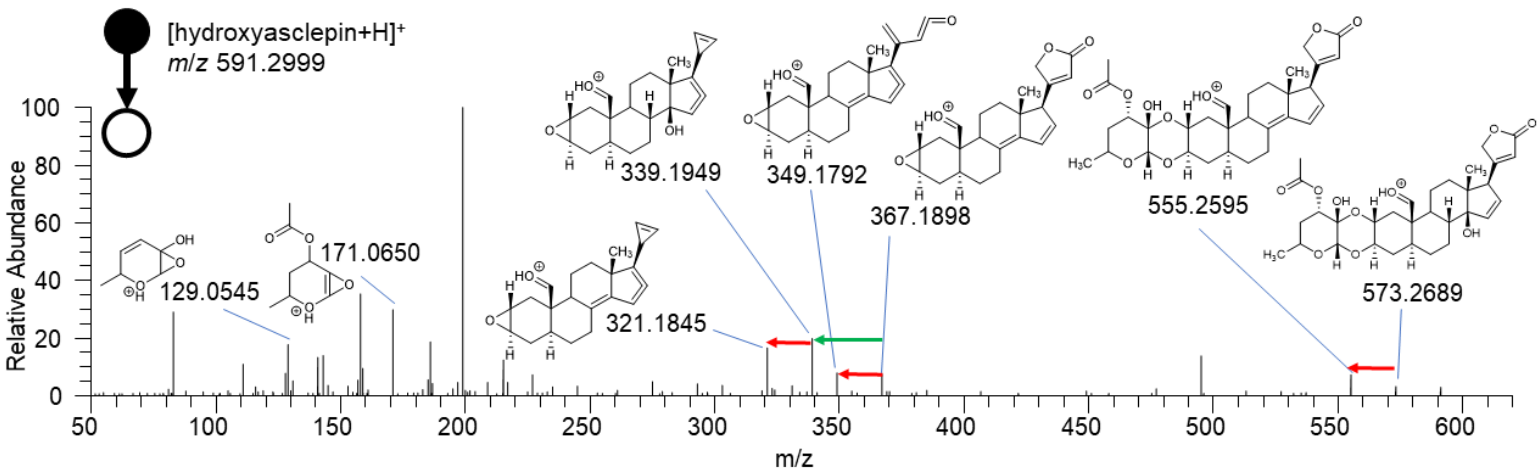

hydroxyuscharin

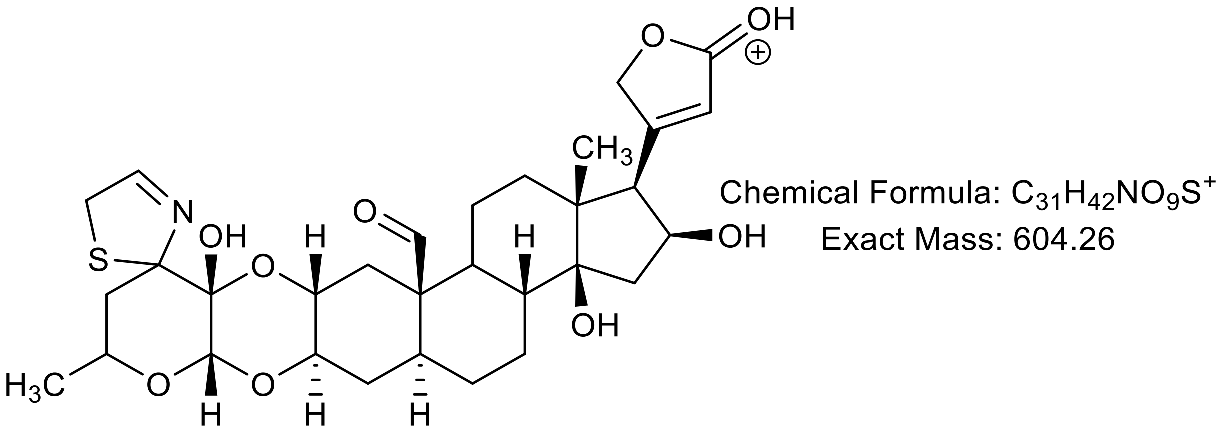

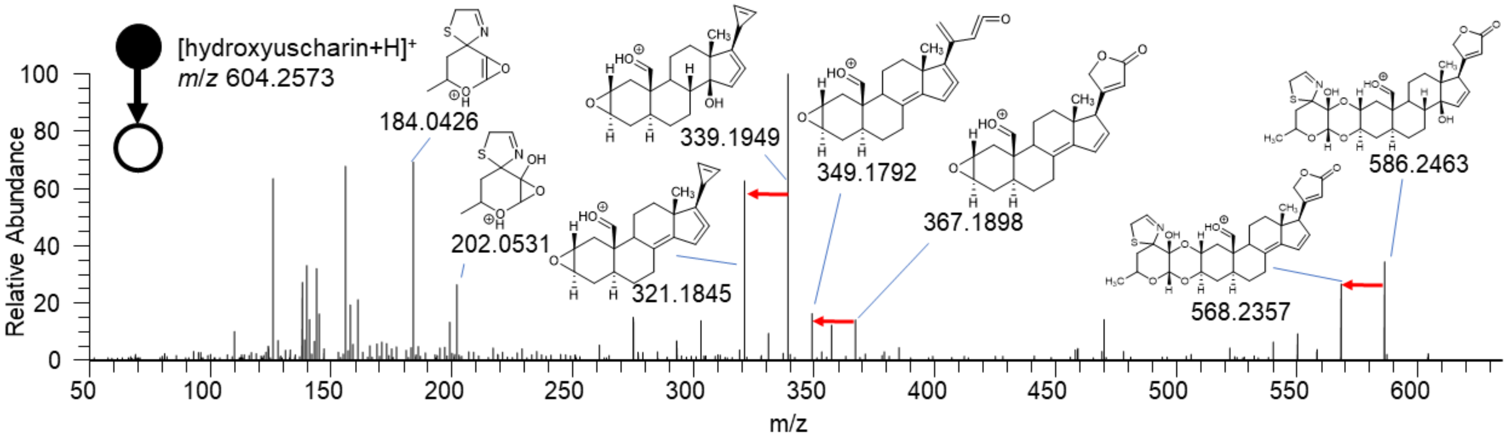

hydroxyvoruscharin

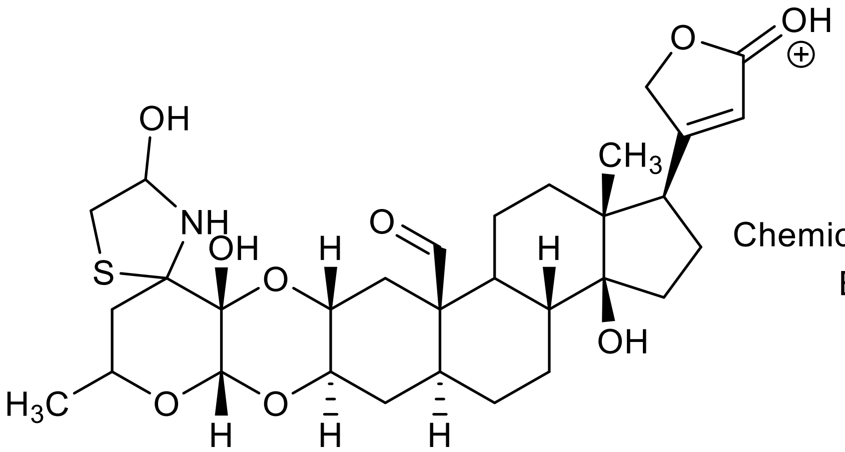

Chemical Formula: $C_{31}H_{44}NO_9S^+$

Exact Mass: 606.27

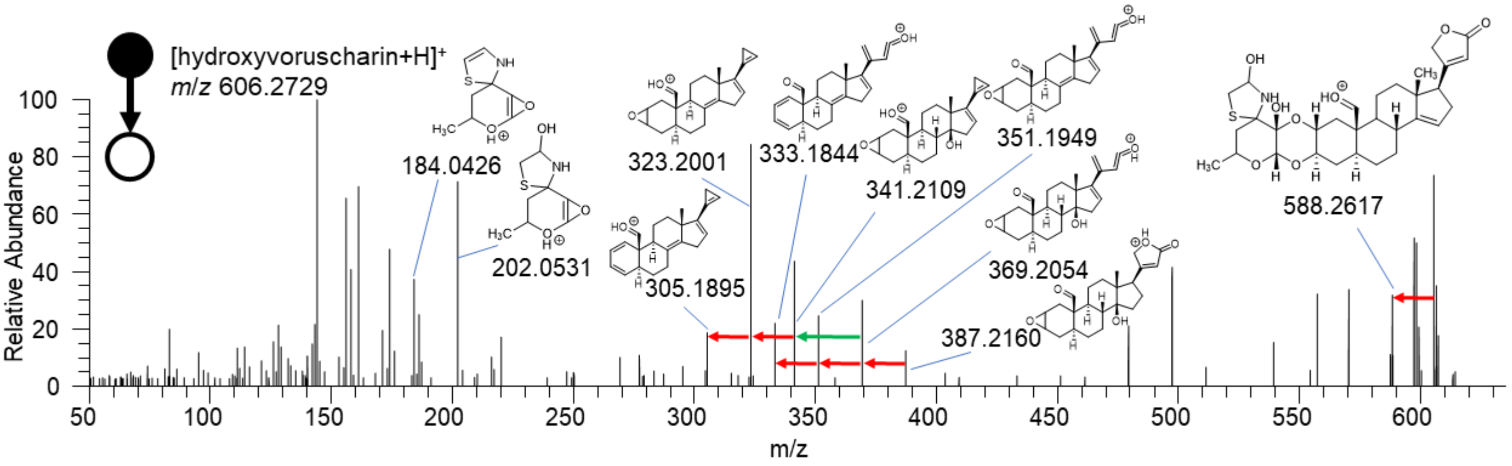

diacetylgofruside

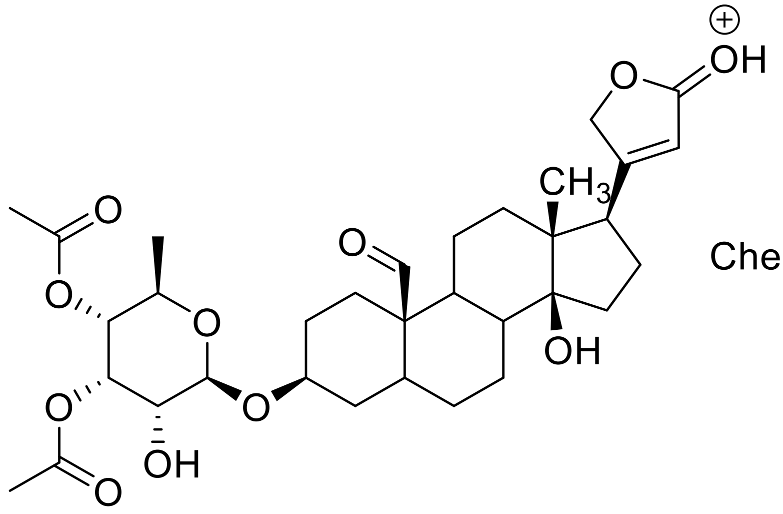

Chemical Formula: $C_{33}H_{47}O_{11}^{+}$
Exact Mass: 619.31

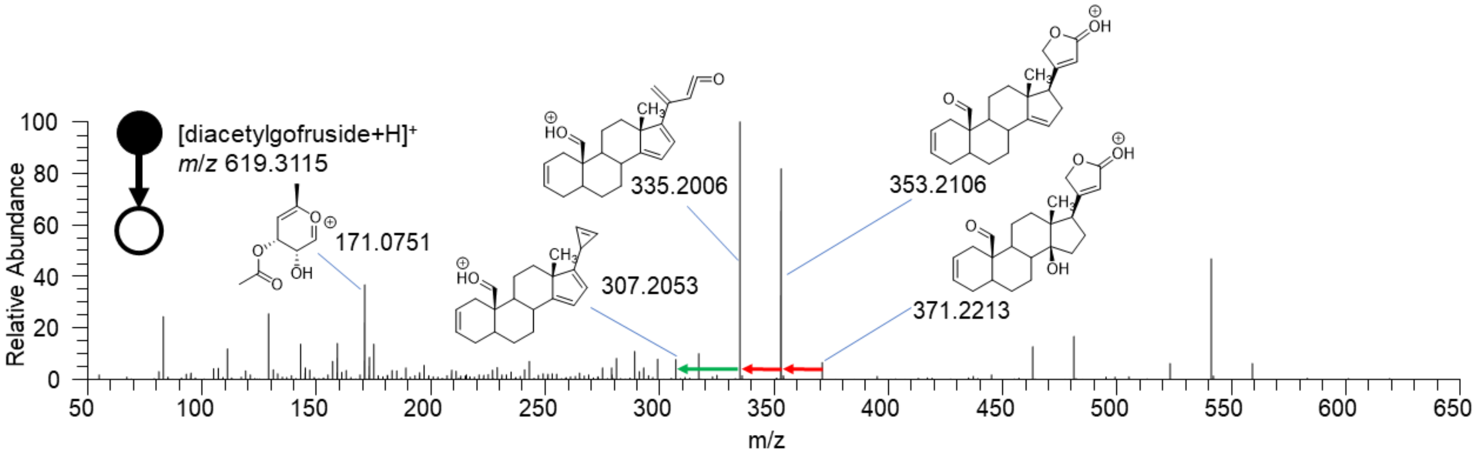

acetoxyasclepin

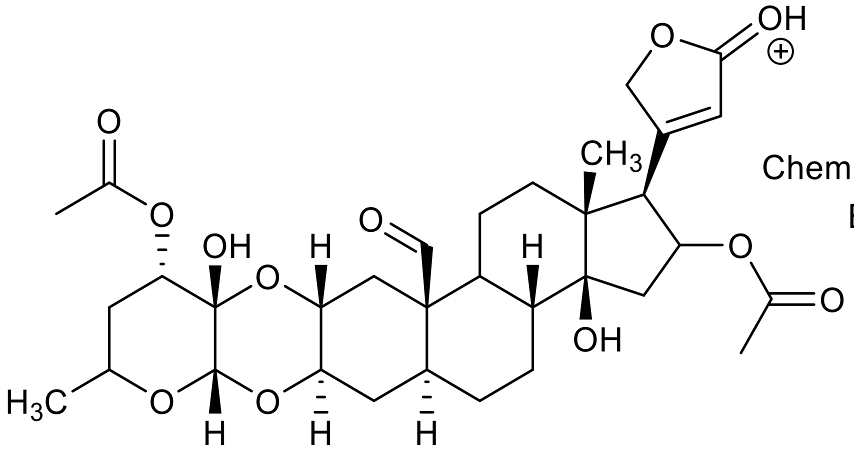

Chemical Formula: $C_{33}H_{45}O_{12}^{+}$
Exact Mass: 633.29

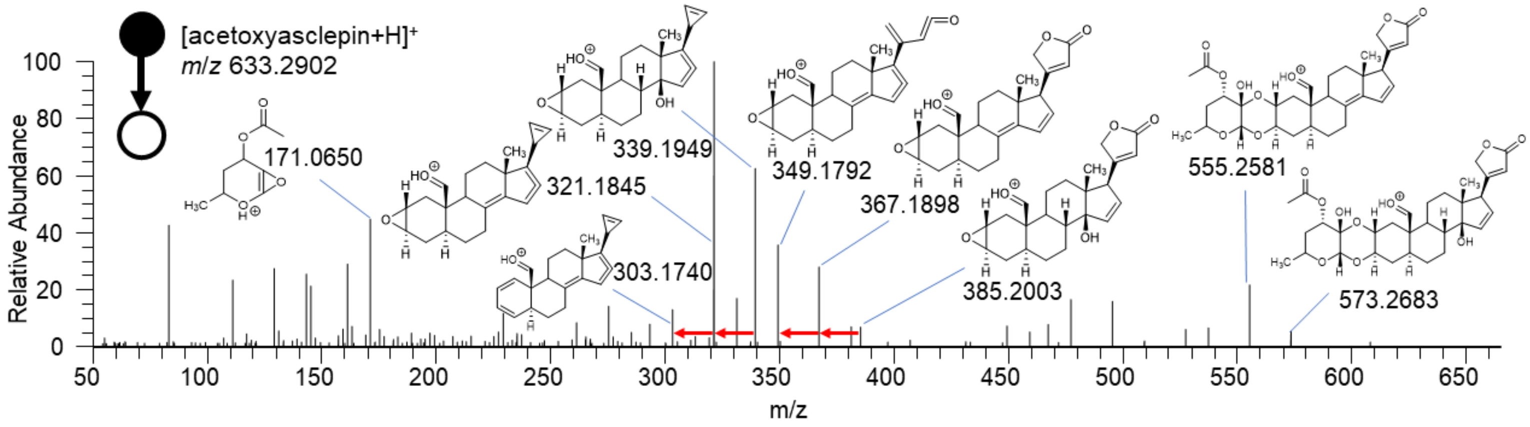

digitoxin bisdigitoxoside

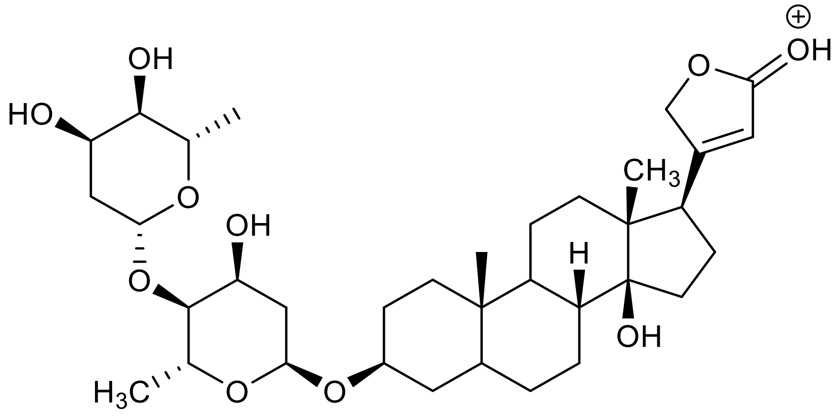

Chemical Formula: $C_{35}H_{55}O_{10}^{+}$
Exact Mass: 635.38

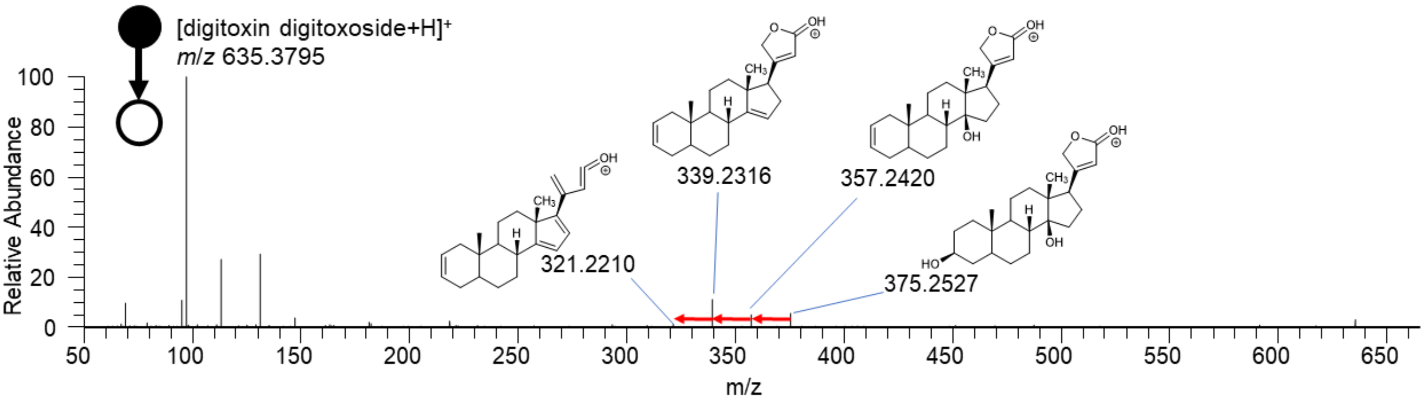

cannogenin-diglycoside

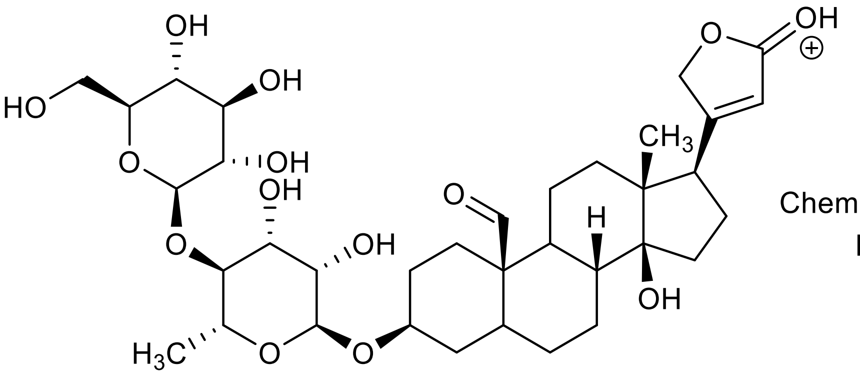

Chemical Formula: $C_{35}H_{53}O_{14}^{+}$
Exact Mass: 697.34

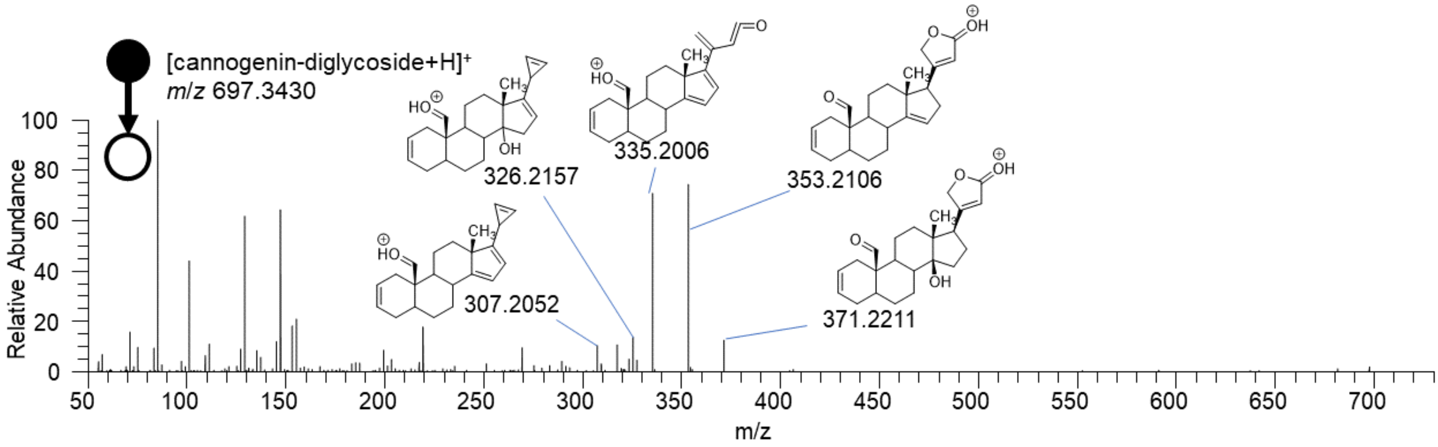

antiaroside B

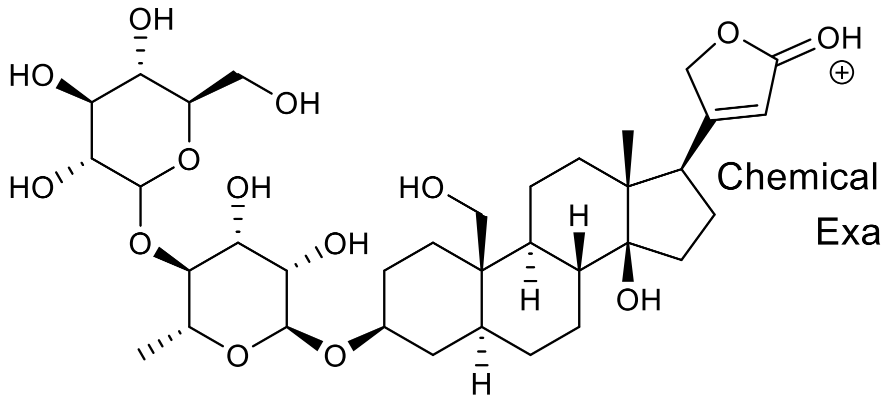

Chemical Formula: $C_{35}H_{55}O_{14}^{+}$
Exact Mass: 699.36

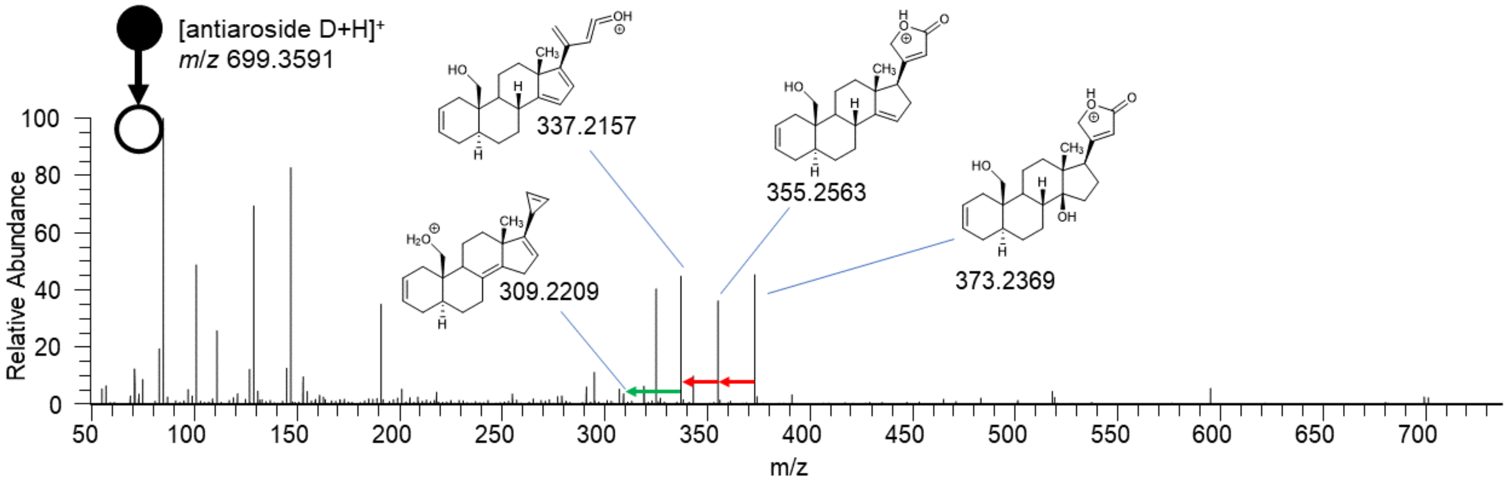

calotoxin-glycoside

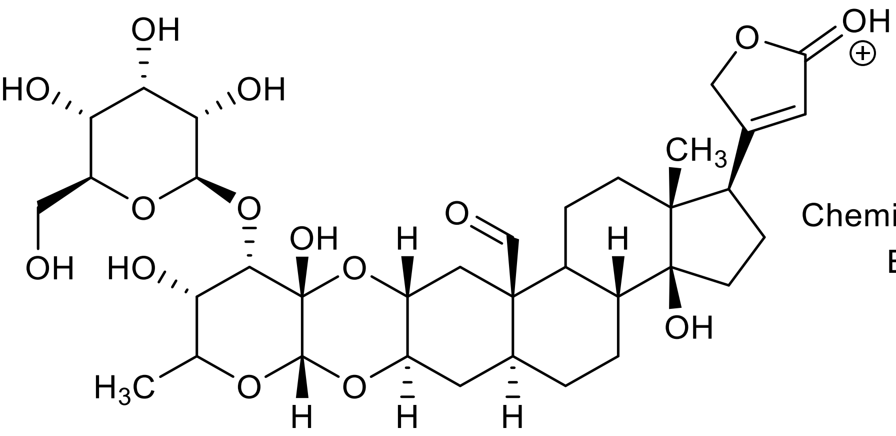

Chemical Formula: $C_{35}H_{51}O_{15}^{+}$
Exact Mass: 711.32

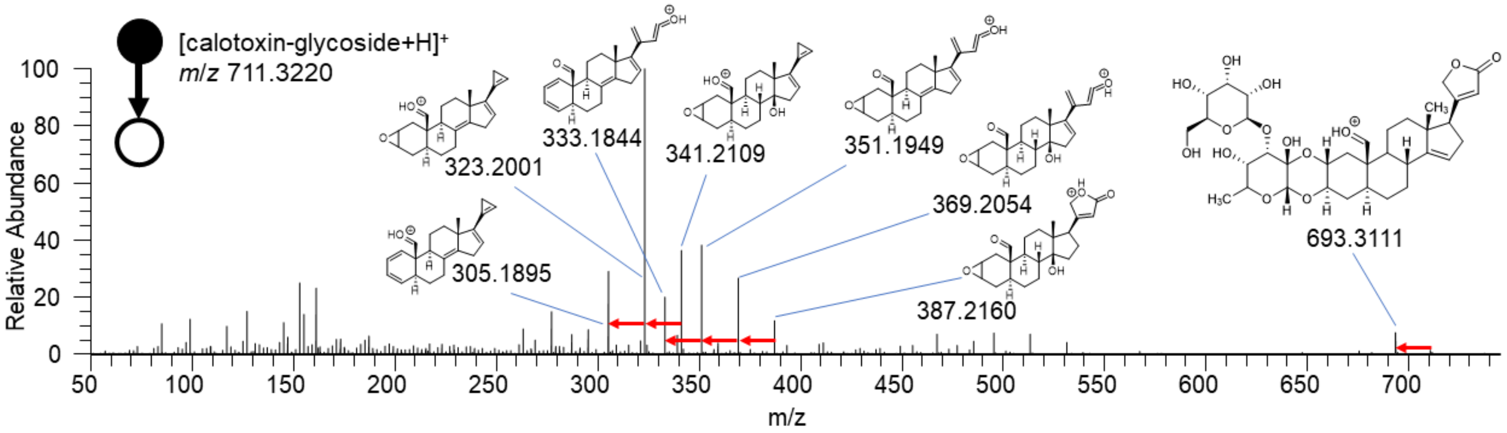

Supplement: Supplementary file 1 — ac2c02694_si_001.pdf [file ac2c02694_si_001.pdf]
